# Changes in life expectancy 1950–2010: contributions from age- and disease-specific mortality in selected countries

**DOI:** 10.1186/s12963-016-0089-x

**Published:** 2016-05-23

**Authors:** Jochen Klenk, Ulrich Keil, Andrea Jaensch, Marcus C. Christiansen, Gabriele Nagel

**Affiliations:** Institute of Epidemiology and Medical Biometry, Ulm University, Helmholtzstrasse 22, 89081 Ulm, Germany; Clinic for Geriatric Rehabilitation, Robert-Bosch Hospital, Auerbachstrasse 110, 70376 Stuttgart, Germany; Institute of Epidemiology and Social Medicine, University of Münster, Albert-Schweitzer-Campus 1, 48149 Münster, Germany; Institute of Insurance Science, Ulm University, Helmholtzstrasse 20, 89081 Ulm, Germany; Maxwell Institute for Mathematical Sciences, Edinburgh, UK; Heriot-Watt University, EH14 4AS Edinburgh, UK

**Keywords:** Life expectancy, Mortality, Gender, Age, Cause-specific, Decomposition

## Abstract

**Background:**

Changes of life expectancy over time serve as an interesting public health indicator for medical, social and economic developments within populations. The aim of this study was to quantify changes of life expectancy between 1950 and 2010 and relate these to main causes of death.

**Methods:**

Pollard’s actuarial method of decomposing life expectancy was applied to compare the contributions of different age- and disease-groups on life expectancy in 5-year intervals.

**Results:**

From the 1960 to 70s on, declines in cardiovascular disease (CVD) mortality play an increasing role in improving life expectancy in many developed countries. During the past decades gains in life expectancy in these countries were mainly observed in age groups ≥65 years. A further consistent pattern was that life expectancy increases were stronger in men than in women, although life expectancy is still higher in women. In Japan, an accelerated epidemiologic transition in causes of death was found, with the highest increases between 1950 and 1955. Short-term declines and subsequent gains in life expectancy were observed in Eastern Europe and the former states of the Union of Soviet Socialist Republics (USSR), reflecting the changes of the political system.

**Conclusions:**

Changes of life years estimated with the decomposing method can be directly interpreted and may therefore be useful in public health communication. The development within specific countries is highly sensitive to changes in the political, social and public health environment.

**Electronic supplementary material:**

The online version of this article (doi:10.1186/s12963-016-0089-x) contains supplementary material, which is available to authorized users.

## Introduction

Since 1840, life expectancy of the best performing country in each year has been increasing almost linearly by 2.5 years per decade [1]. Japan achieved the world’s highest life expectancy over the past 50 years, starting from a very low level at the end of World War II [1, 2], while Japan’s health expenditures are relatively low compared to other nations [2].

From a conceptual perspective, the development of mortality in populations follows similar patterns. The epidemiological transition theory states that when nations improve their economic situation, communicable diseases like infectious and parasitic diseases will be replaced by non-communicable diseases like cardiovascular diseases (CVD) and cancer [3, 4]. However, the speed of development is affected by various factors. The large variations can be attributed to different economic situations, nutritional and lifestyle factors, work-related and social factors, as well as public health strategies and medical care [5]. In order to set up sound public health programs for healthy aging, it is important to know which causes of death and which age groups harbor the greatest potential for improving life expectancy.

Since 1950, the World Health Organization (WHO) has been collecting data on mortality and causes of death. Life expectancy is a convenient and important summary measure of mortality, and more intuitive than mortality rates. The method suggested by Pollard estimates the absolute contribution of each cause of death and each age group expressed as life years to the change of life expectancy [6]. This measure can serve as an interesting indicator for social and economic developments within a population. An advantage of this method is that the results can be directly interpreted as changes of life years, and may therefore be used directly in public health communication.

The objectives of this study were to give a longitudinal overview of the contributions of age- and disease-specific mortality rates to the changes in life expectancy, as exemplified and presented for selected countries between 1950 and 2010.

## Material and methods

### Database

The mortality database of the World Health Organization (WHO) was used for the present analyses (date of extraction: December 2013) [7], comprising sex-, age- and disease-specific deaths, as well as corresponding population numbers registered in national civil registration systems from 123 countries for each calendar year between 1950 and 2011. During the reporting period some countries were divided (e.g., Yugoslavia) or unified (e.g., Germany). Since data were not continuously available for all countries or for the entire observation period, gaps in the timeline exist. In the current analysis only sovereign states with time intervals of continuous data reporting over at least 10 years were considered to produce more robust estimates (*n* = 77). Countries with reporting periods ending before 1990 were excluded as well as those with a total average population of less than one million to reduce random effects due to small numbers of death. Finally, data from 64 countries were analyzed. We further selected five countries with high life expectancy but varying health expenditures for in-depth analyses: Japan, Switzerland, Singapore, Germany and the US (Table 1). Overall, the data quality and completeness of the included death registers are very heterogeneous [8]. In 2003 more than 80 % had a completeness of 100 %, while 12 % had less than 90 % completeness, with a minimum of 54 % in China [8]. The proportion of deaths coded to ill-defined codes ranged from 3 % in Singapore and Finland to 26 % in Greece. In all of the five selected countries, however, the completeness was 100 %, with the proportion of deaths coded to ill-defined codes ranging from 3 to 14 %.

**Table 1:** Characteristics of selected countries

Country	Observation period	Total population^a^	Life expectancy women^a, b^	Life expectancy men^a,b^	Gross national income per capita (US$)^a, b^	Total health expenditure per capita (int $)^a, b, c^	Total health expenditure as % of gross domestic product^a, b^
**Japan**	**1950–2010**	**127,450,459**	**86.3**	**79.6**	**42,190**	**3,237**	**9.6**
Hong Kong SAR	1960–2010	7,024,200	86.0	80.1	33,620	NA	NA
France	1955–2005	65,023,142	85.3	78.2	43,790	4,039	11.7
Italy	1955–2000	59,277,417	85.0	79.8	37,690	3,162	9.4
Spain	1955–2010	46,576,897	84.7	78.7	32,130	3,026	9.6
**Switzerland**	**1955–2010**	**7,824,909**	**84.5**	**80.1**	**77,360**	**5,319**	**10.9**
Republic of Korea	1985–2010	49,410,366	84.1	77.2	21,320	2,069	7.3
Australia	1950–2000	22,031,800	84.0	79.5	46,490	3,761	8.9
**Singapore**	**1965–2010**	**5,076,700**	**84.0**	**79.2**	**44,790**	**2,773**	**4.1**
Israel	1975–2010	7,623,600	83.6	79.7	29,480	2,078	7.6
Sweden	1955–2010	9,378,126	83.5	79.5	53,810	3,762	9.5
Norway	1955–2010	4,889,252	83.2	78.9	86,830	5,475	10.0
Canada	1950–2005	34,005,274	83.2	78.7	44,450	4,468	11.4
Austria	1995–2010	8,389,771	83.2	77.7	49,180	4,517	11.6
Finland	1955–2010	5,363,352	83.2	76.7	49,320	3,297	9.0
Ireland	1950–2005	4,560,155	83.1	78.5	43,760	3,796	9.3
Greece	1965–2010	11,153,454	83.0	77.9	27,580	2,685	9.4
Belgium	1955–1995	10,920,272	83.0	77.6	47,200	4,058	10.5
Netherlands	1950–2010	16,615,394	82.7	78.8	53,320	5,063	12.1
New Zealand	1950–2005	4,367,800	82.7	78.8	28,980	3,034	10.2
Slovenia	1985–2010	2,048,583	82.7	76.3	24,540	2,452	8.9
Germany, Former Federal Republic	1955–1990	1990: 63,253,800	1989: 78.6	1989: 72.1	1990: 20,630	NA	NA
**Germany**	**1990–2010**	**81,776,930**	**82.6**	**77.5**	**44,780**	**4,426**	**11.5**
United Kingdom	1950–2010	62,766,365	82.4	78.5	40,470	3,223	9.6
Chile	1955–1990	17,150,760	82.1	76.2	10,730	1,309	7.1
Portugal	1955–2000	10,573,100	82.1	76.1	22,930	2,810	10.8
Puerto Rico	1970–1990	3,721,208	82.1	74.4	16,650	NA	NA
Costa Rica	1965–1995	4,669,685	81.7	77.0	6,910	1,167	9.7
Denmark	1955–2005	5,547,683	81.2	77.1	60,820	4,545	11.1
**United States of America**	**1950–2005**	**309,326,295**	**81.0**	**76.2**	**49,110**	**8,299**	**17.7**
Cuba	1970–1995	11,281,768	80.8	76.7	5,630	1,823	10.6
Czechoslovakia, Former	1955–1990	1991: 15,592,000	NA	NA	NA	NA	NA
Czech Republic	1990–2010	10,474,410	80.6	74.4	19,210	1,930	7.4
Slovakia	1995–2010	5,391,428	78.8	71.6	17,130	2,039	9.0
Poland	1960–2010	38,183,683	80.6	72.1	12,580	1,432	7.0
Estonia	1985–2010	1,331,475	80.5	70.6	14,390	1,300	6.3
Uruguay	1955–1990	3,371,982	80.2	73.2	10,110	1,423	8.7
Yugoslavia, Former	1965–1990	1990: 23,817,900	NA	NA	NA	NA	NA
Croatia	1985–2010	4,417,781	79.6	73.5	13,740	1,607	7.8
Serbia	2000–2010	7,291,436	76.6	71.4	5,850	1,191	10.7
Argentina	1980–1995	40,374,224	79.5	72.0	2005: 4,450	1,312	8.2
Mexico	1960–1995	117,886,404	79.2	74.3	8,730	1,003	6.3
Lithuania	1985–2010	3,097,282	78.8	68.0	12,260	1,387	7.0
Latvia	1980–2010	2,097,555	78.4	68.8	12,680	1,145	6.5
Hungary	1955–2010	10,000,023	78.1	70.5	13,050	1,701	8.0
Armenia	1985–2000	2,963,496	77.7	70.9	3,370	291	4.6
Romania	1970–2010	20,246,871	77.3	69.8	8,430	964	5.9
Venezuela	1955–1990	29,043,283	77.2	71.3	11,520	795	4.7
Bulgaria	1965–2010	7,395,599	77.2	70.0	6,630	1,088	7.6
TFYR Macedonia	1995–2010	2,102,216	77.0	72.5	4,570	778	7.0
Mauritius	1965–2005	1,280,924	76.7	69.5	7,780	813	5.4
Belarus	1985–1995	9,490,000	76.5	64.6	5,990	855	5.6
China,select urban and rural areas	1990–2000	1,337,705,000	76.2	73.6	4,240	441	5.0
Ukraine	1985–2005	45,870,700	75.5	65.3	2,990	599	7.8
Kuwait	1975–2000	2,991,580	75.1	73.2	42,920	2,286	2.8
Russian Federation	1980–2010	142,385,523	74.9	63.1	10,010	1,410	6.3
Azerbaijan	1985–2000	9,054,332	73.6	67.4	5,370	831	5.3
Kyrgyzstan	1985–2005	5,447,900	73.5	65.3	850	186	6.7
Trinidad and Tobago	1970–1990	1,328,095	73.3	66.1	15,800	1,494	5.2
Kazakhstan	1985–2010	16,321,581	73.3	63.5	7,440	871	4.3
Republic of Moldova	1985–2010	3,562,045	72.4	64.7	1,820	448	11.7
Uzbekistan	1985–2005	28,562,400	71.3	64.6	1,300	226	5.4
Tajikistan	1985–2000	7,627,326	70.4	63.8	730	125	6.0
Turkmenistan	1985–1995	5,041,995	69.3	60.9	4,070	196	2.1

Countries with high life expectancy but varying health expenditures who were selected for in-depth analyses are highlighted in bold text

^a^Data from the year 2010 unless otherwise noted

^b^Data from World Bank

^c^Health expenditure per capita in international $ considering country-specific purchasing power parity (PPP) rates

Disease-specific causes of death were reported according to the International Classification of Diseases (ICD). During the observation period four versions of the ICD were used (1962–1968: ICD 7, 1969–1978: ICD 8, 1979–1997: ICD 9 and 1998–2011: ICD 10), which were transferred to ICD 10. To handle coding imprecision, and to have a sufficient number of deaths even for small countries, only main disease groups were considered. The selected causes of mortality account for more than 90 % of all recorded deaths: infectious and parasitic diseases (ICD 10: A00-B99), malignant neoplasms (C00-C97), diseases of the circulatory system (I00-I99), diseases of the respiratory system (J00-J99), diseases of the digestive system (K00-K93), perinatal diseases (P00-P96) and external causes of mortality (V01-Y98). All other causes of death were combined as “other diseases”.

### Analysis

An actuarial method of decomposing life expectancy was used to estimate the contributions of different age groups and disease-specific causes of death to the changes in life expectancy [9]. This method proposed by Pollard was selected because it allows for simultaneously decomposing both different age groups and different causes of death. It is also nonparametric, avoiding strong model assumptions. Furthermore, the method has a simple and intuitive output, and refrains from splitting effects into different subtypes. For example, it is possible to calculate that the contribution of the reduction in cardiovascular mortality to the increased life expectancy in Japanese women between 2000 and 2005 was 0.47 years. Considering the cause-specific mortality difference in every single age group within a specific time interval (e.g., 1 year), the difference Δ in average life expectancy at birth between two points in time, denoted in the following by a superscript 1 or 2, is given by the formula:

$$ {\Delta}^{2-1}={e}_0^2-{e}_0^1={\displaystyle \sum_{i=1}^n{\displaystyle \sum_{x=0}^{\omega}\left({}_i{Q}_x^1{-}_i{Q}_x^2\right)}}\cdot {w}_x\;\mathrm{with}\;{w}_x=\frac{1}{2}\left({}_x{p}_0^2{e}_x^1{+}_x{p}_0^1{e}_x^2\right) $$

where *e*_*x*_^*1*^ and *e*_*x*_^*2*^ are the life expectancies at age x for specific points in time 1 and 2; n denotes the number of considered causes of death and ω the last included age interval; _*i*_*Q*_*x*_ is the mortality rate of the i-th cause of death at age interval x with the weight w_x_. _*x*_*p*_*0*_^*1*^ and _*x*_*p*_*0*_^*2*^ denoting the probability of living from birth to age x at time point 1 and 2 derived from the corresponding life tables. Since mortality rates in the WHO database were reported for 5-year age groups the equation had to be adapted accordingly. To adjust for short-term variability, a weighted moving average technique was applied to mortality rates and population numbers [10]. The width of the moving window was 3 years.

For every country the age- and disease-specific change in life expectancy was calculated for adjacent time intervals of 5 years starting in 1950 and ending in 2010 (1950–55, 1955–1960, …, 2005–2010) if continuous mortality and population data were available for the specific interval. The age-specific change in life expectancy was reported in five categories: 0–4, 5–14, 15–39, 40–64 and ≥65 years. As indicators for the epidemiological transition, time intervals where highlighted by different colors in Tables 2 and 3 if the highest contribution to life expectancy was due to cardiovascular diseases (green), the age group ≥65 years (blue) or both (red). This contribution had to be present for at least two subsequent time intervals with not more than one gap between the intervals. All calculations were performed using SAS 9.2.

**Table 2:**
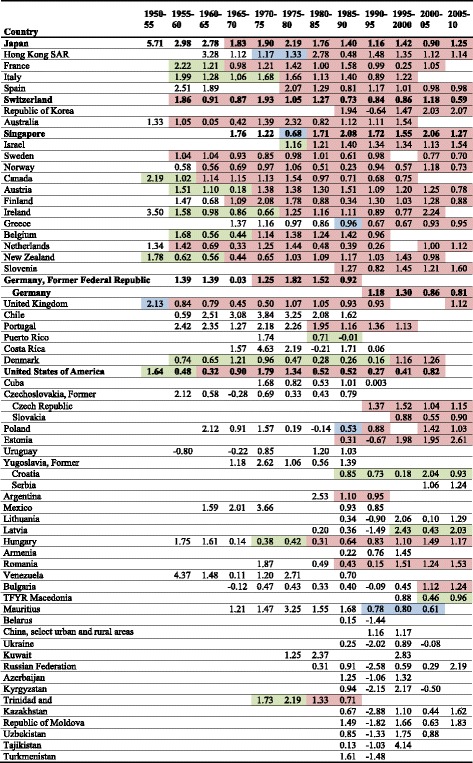
Overall change in life expectancy [years] by 5-year interval in women

Green: cardiovascular diseases are the most important cause contributor but age group 65 years and older is NOT the most important age contributor, blue: age group 65 years and older is the most important age contributor but cardiovascular diseases are NOT the most important cause contributor, red: cardiovascular diseases are the most important cause contributor AND age group 65 years and older is the most important age contributor

**Table 3:**
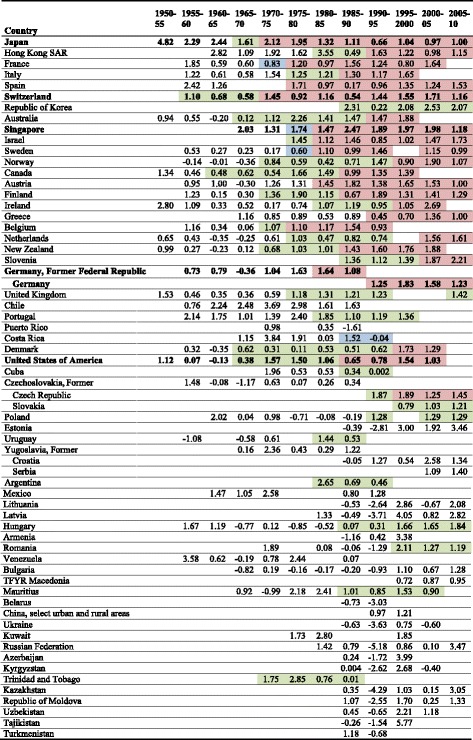
Overall change in life expectancy [years] by 5-year interval in men

Green: cardiovascular diseases are the most important cause contributor but age group 65 years and older is NOT the most important age contributor, blue: age group 65 years and older is the most important age contributor but cardiovascular diseases are NOT the most important cause contributor, red: cardiovascular diseases are the most important cause contributor AND age group 65 years and older is the most important age contributor

## Results

The characteristics of the selected countries referenced for 2010 and sorted by life expectancy are presented in Table 1. According to the classification of the United Nations, 36 countries were from Europe, 14 from Asia, nine from Latin America and the Caribbean, two from North America, two from Oceania and one from Africa. Life expectancy for women ranged from 69.3 years in Turkmenistan to 86.3 years in Japan and for men from 60.9 years in Turkmenistan to 80.1 years in Hong Kong and Switzerland. The lowest Gross National Income (GNI) per capita was reported in Tajikistan (US$730) and the highest in Norway (US$86,830). Total expenditures on health in relation to GNI varied considerably between countries. For example in Singapore, Japan, Switzerland, Germany and the US 4.1, 9.6, 10.9, 11.5 and 17.7 % were reported, respectively. In contrast, differences in life expectancy between these countries were less pronounced, with the US trailing behind.

In most of the investigated countries positive values of the relative change in life expectancy by 5-year intervals were observed in women (Table 2) and men (Table 3) indicating an increase in life expectancy. Extremely positive changes in life expectancy were observed in Japan between 1950 and 1955: for men and women the overall increase in life expectancy was 4.8 and 5.7 years, respectively. Tremendously negative changes were seen among men and women (relative decline −5.2 and −2.6 years, respectively) in the former Soviet Union between 1990 and 1995, while life expectancy increased afterwards. In Germany life expectancy increased since the 1970s between around 1–2 years per 5-year time interval in both men and women. Although in most of the developed countries female life expectancy is still higher than male life expectancy, the increase in life expectancy was sharper in men during the last decades. This, however, was not true for Japan.

**Fig. 1:**
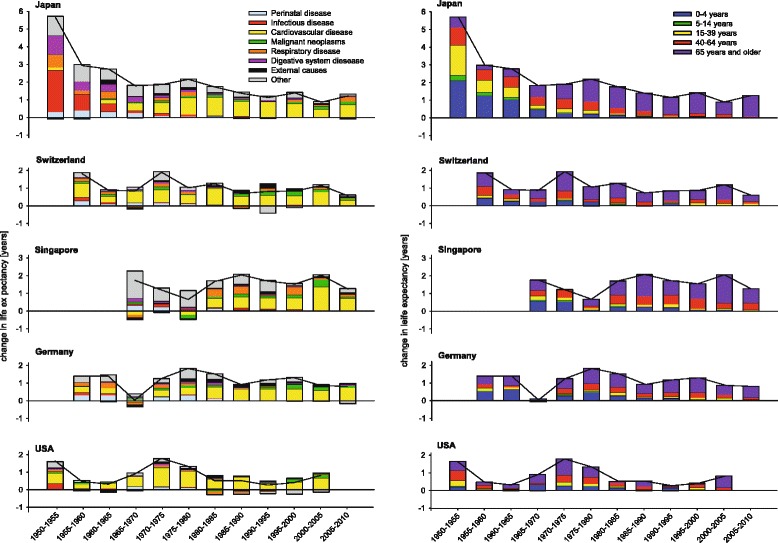
Five-year contribution of different age- and disease-groups to the changes in life expectancy in women between 1950 and 2010 in Japan, Switzerland, Singapore, Germany and the United States

**Fig. 2:**
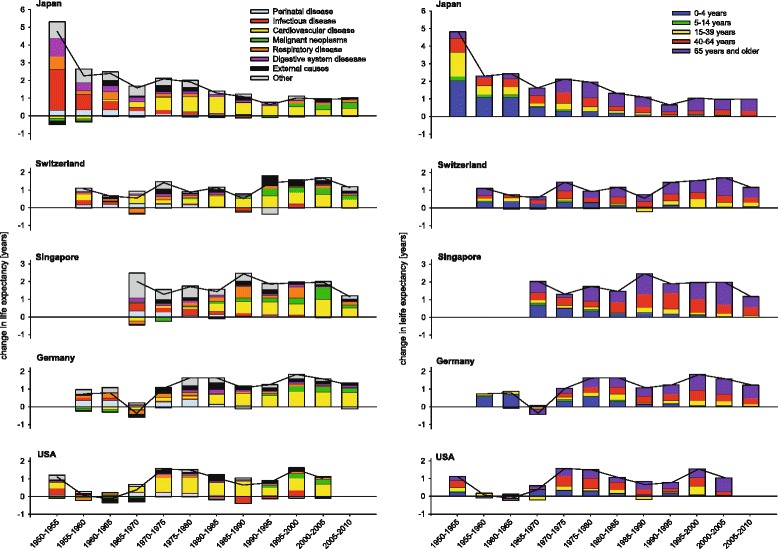
Five-year contribution of different age- and disease-groups to the changes in life expectancy in men between 1950 and 2010 in Japan, Switzerland, Singapore, Germany and the United States

As examples for different developments in life expectancies among countries with high life expectancy we present the cause- and age-group-specific data of Japan, Switzerland, Singapore, Germany and the US for women (Fig. 1) and men (Fig. 2) in order to explore differences. Particular patterns will be highlighted. In Japan after World War II the increase in life expectancy was exceptionally marked until 1965. Successively the contribution of the oldest age group increased, becoming dominant after 1965. Switzerland reached its comparably high life expectancy with a different more monotonic pattern. CVD was the most important contributor across the whole observation period. Even in the last period from 2005 to 2010 the relative contribution of CVD to the change in life expectancy was remarkable. In Japan, the United States, Germany, Singapore and Switzerland the contributions of CVD for women were 94, 78, 75, 61, 58 % and for men 67, 73, 59, 49, 48 %, respectively.

In Germany, a remarkable relapse in the development of life expectancy was observed in men and women between 1965 and 1970. During the fall of the Iron Curtain only a small effect on German life expectancy was seen. In contrast, a tremendous decrease in life expectancy was observed for Russian men and to a lesser extent for women between 1990 and 1995. Mortality increased mainly in persons aged 15–64 years, and was due to CVD and external causes. During the past decades this was counterbalanced by increases in life expectancy. In the US, the patterns of changes in life expectancy were heterogeneous, with highest increases in life expectancy in the 1970s mainly due to CVD. The patterns observed in Singapore suggest a delayed epidemiological transition, with increasing contributions of CVD since 1980. In the following 30 years the increase in life expectancy was particularly high with an increase of more than 10 life years in men and women. Starting in the 1990s, a decline in mortality due to malignant neoplasms led to a small but notable contribution to the increase in life expectancy.

Considering age- and disease-specific contributions to life expectancy for the investigated countries some basic patterns could be identified (see Tables 2 and 3, as well as Additional file 1: Tables S1–S4). In women (Table 2) declining cardiovascular disease death rates, often in combination with changes in the highest age group (red color), became the dominant cause for reductions in all-cause mortality in the 1950s and 60s for North America, most western European countries and for Japan, Australia and New Zealand. After the fall of the Iron Curtain in the 1990s, the same pattern was seen for most of the future members of the European Union (e.g., Hungary) but not for all (e.g., Poland).

The contribution of the age group 65 years and older to the change in life expectancy was dominant already in the 1950s in a few Western European countries and Australia. Corresponding to the cause of death specific development, this age group (≥65 years) gained importance in the following years for more and more countries. For men, the development of the age- and cause-specific contributions was similar, but in most countries was delayed for up to two decades (e.g., France). In the time period 1990–1995, directly after the fall of the Iron Curtain, a remarkably steep decrease of life expectancy was seen in the age groups “40–64 years” and “65 years and over” in all countries of the former USSR. The decrease was strongest in Russian men with a loss of 5.2 life years within this 5-year interval.

## Discussion

Starting in the early 1960s and 1970s CVD mortality declines played an increasing role in improving life expectancy in many Western European countries as well as in Japan, Singapore, Australia, New Zealand and the United States. In these countries gains in life expectancy during the past decades were mainly observed in age groups ≥65 years particularly in women. The development for men was delayed for up to two decades compared to women. In Japan, an accelerated epidemiologic transition in causes of death from infectious and respiratory to CVD and from young age-groups to older age groups was found. In Eastern Europe, Russia and some other former states of the Union of Soviet Socialist Republics, periods with declines in life expectancy particularly in men were observed.

Compared to population-based morbidity data, life expectancy at birth as a measure of mortality is a valid and important indicator of a population’s health status. It is easy to interpret and is intuitive for both layman and experts. The method of decomposing total life expectancy with respect to age- and disease-specific contributions is commonly used in demographical but rarely in public health and epidemiological research. However, this approach allows translating the immediate influence of changes in lifestyle as well as socio-economic and political living conditions on mortality rates into age- and disease-specific contributions to short-term changes in life expectancy.

Limitations of our study are related to data gaps concerning completeness and information of causes of death which may have affected some of the observed differences [8]. Causes of death have been coded using varying ICD versions [7–10]. Therefore, code conversion was necessary, which may have resulted in some conversion imprecision. Differences in the application of the classification of causes of death could have introduced bias as well. However, using only main disease-groups should have reduced bias to a minimum. The completeness and reporting quality differed between countries [8]. Many countries were excluded due to missing long-term data. In some of the analyzed countries and time intervals the proportion of deaths coded as ‘symptoms, signs and abnormal clinical and laboratory findings’ is high and could have introduced bias. Due to the structure of the WHO Mortality Database, it was not possible to redistribute these deaths to specific causes. This may underestimate the true contribution of a specific cause of death and increase the contribution of “others”.

Gender differences in life expectancy are present in all countries. For example, in Japan the absolute number was 6.7 years in 2010. The mortality rates at older ages decreased faster in women than in men [11, 12]. Gender differences in CVD mortality vary over time and geographically [13]. It has been suggested that the difference in life expectancy between men and women could be partly related to biological factors [14]. A greater difference is thought to be related to environmental factors [13]. Also gender-related factors such as risky behavior of men and differences in body size, hormonal factors and innate immunity could contribute to the observed differences in human mortality [15, 16]. The lower CVD mortality in women [17] can be explained by differences in diet and lifestyle factors [18].

The epidemiological transition provides the theoretical background for the massive improvement in life expectancy during the 20th century [4]. The stage of the epidemiological transition differed between countries. The transition from communicable to non-communicable diseases as the main causes of deaths marks a new phase in the mortality development. Today, most premature deaths are preventable, and a major part are attributed to non-communicable disease (NCD), mainly heart disease, stroke, cancer, diabetes and chronic respiratory diseases [19]. Since the 1980s several criticisms of the epidemiological transition have been brought forward, since it requires morbidity and mortality data over long time periods. These data are often lacking in middle- and low-income countries. Therefore, it is important to promote and establish systematic health reporting especially in those countries.

The developments in Japan since World War II illustrate the concept of epidemiological transition within a very short time period. Since 1986 Japan has ranked first for female life expectancy, with the worldwide highest life expectancy of 86.3 years in 2010 [20]. Substantial gains in longevity occurred between 1950 and 1965 by the reduction of infant and young adult mortality. The control of intestinal and respiratory infection, and the implementation of vaccination programs may have contributed to this development [2, 21]. When communicable diseases had been controlled in the seventies, life expectancy continued to rise due to declines in mortality from NCDs.

With regard to recent evaluations of the health care system, medical care as well as total expenditures on health in Japan seem to be average [2]. However, the interplay between general favorable risk factors, the country’s educational profile and broad prevention programs with universal coverage based on strong government action may explain why life expectancy in Japan increased so rapidly and keeps this leading position compared to other countries with much larger total expenditure on health care like the US [22]. Traditionally, the Japanese diet is rich in rice, salt, soy and fish intake, and low in fat intake. With regard to high salt consumption, especially in Northern Japan, public health programs were developed and hypertension was targeted and treated as a major public health problem [2].

Our observation that among women CVD mortality declines became the most prominent cause for reductions in all-cause mortality in 1950s and 1960s in many industrialized countries is consistent with other reports and clearly shows the effectiveness of prevention programs related to smoking behavior, alcohol consumption and physical activity as well as improved treatment [23–25]. However, some of these changes might have been due to infectious diseases and other sources of inflammation [26].

An example to the contrary is Russia’s extreme drop in life expectancy after 1990. The rise in mortality between 1990 and 1995, particularly in young-middle aged men, has been attributed to the economic and social instability during that period, which may have caused stress, anxiety and depression [27]. After 1995 life expectancy in Russia for both genders improved, with a sharp increase between 2005 and 2010. Germany after its unification showed the opposite development. After adapting to the living conditions of Western Germany, life expectancy of people in former Eastern Germany increased by 3.2 years within a period of just 7 years [28].

The small gains in life expectancy in the US in recent years may be partly due to the high prevalence of obesity [29]. In addition, social inequalities are likely to result in ineffective prevention and health-care interventions. The systems of countries like Japan and Sweden, which have less social inequality and more equal access to the medical system and treatment, are likely to be more effective at increasing life expectancy [30].

Global key actions to reduce the burden of NCD have been identified by the WHO [31]. The WHO has proposed a 25 % reduction of NCD mortality by 2025. Modifiable risk-factors for NCDs have been identified, and there is evidence at the individual and population level that large-scale prevention has the potential to lower the individual disease burden and to save lives [32]. The prevention of NCDs has been ranked a top global priority for economic development [31]. For countries with high health expenditures like the US, Germany and other countries of central Europe, the implementation of acceptable cost-effective multi-sectorial prevention programs on tobacco control and salt reduction, as well as the promotion of healthy diet and physical activity, are suitable to handle and contain health expenditures in aging populations [19]. For low- and middle-income countries, stepwise approaches to implement prevention activities have high priority to prevent pre-mature death and to maintain workforce [33, 34].

Taken together, gains in life expectancy, mainly due to reduction in CVD mortality, have characterized the past four decades in many industrialized countries, first present in women then in men. Overall, CVD is still the leading cause of death worldwide, and most deaths occur in low- and middle-income countries [30]. Expenditures on health varied considerably across countries. Although the absolute health expenditure in the US is four times the value of Singapore, life expectancy in the US lags behind Singapore by 3 years in women and men. The comparison of different health systems may provide further clues for the effective and successful allocation of resources and best approaches for healthy ageing. Well-balanced health politics considering primary and secondary prevention, as well as individually tailored evidence-based treatment, are requirements for maintaining or increasing life expectancy.

## Conclusions

In most countries life expectancy is increasing. Although the contribution of different causes of death as well as age groups changed over time, the overall trend of the life expectancy development seems to be quite stable. There is heterogeneity in the achievements to reduce mortality and improve longevity. For long-lasting improvements in life expectancy in countries with moderate health expenditures like Japan, the combination of various strategies is necessary to implement successful and cost-effective prevention programs and to improve social conditions.

## Additional file

Additional file 1: Table S1. Relative change in life expectancy [years] by 5-year interval in women according to cause of death*. **Table S2** Relative change in life expectancy [years] by 5-year interval in men according to cause of death*. **Table S3** Relative change in life expectancy [years] by 5-year interval in women according to age of death*. **Table S4** Relative change in life expectancy [years] by 5-year interval in men according to age of death*. (DOCX 481 kb)
