# Supplementary material for: Changes in life expectancy 1950–2010: contributions from age- and disease-specific mortality in selected countries
Source: Popul Health Metr. 2016 May 23;14:20. doi: 10.1186/s12963-016-0089-x (PMC4877984; doi:10.1186/s12963-016-0089-x)
Supplement: Additional file 1: Table S1. — Relative change in life expectancy [years] by 5-year interval in women according to cause of death*. Table S2 Relative change in life expectancy [years] by 5-year interval in men according to cause of death*. Table S3 Relative change in life expectancy [years] by 5-year interval in women according to age of death*. Table S4 Relative change in life expectancy [years] by 5-year interval in men according to age of death*. (DOCX 481 kb) [file 12963_2016_89_MOESM1_ESM.docx]

**Additional file 1**

**Table S1:** Relative change in life expectancy [years] by five-year interval in women according to cause of death*

| **Country** | **Cause of death** | **1950-55** | **1955-60** | **1960-65** | **1965-70** | **1970-75** | **1975-80** | **1980-85** | **1985-90** | **1990-95** | **1995-2000** | **2000-05** | **2005-10** |
| --- | --- | --- | --- | --- | --- | --- | --- | --- | --- | --- | --- | --- | --- |
| Argentina | Infectious diseases |  |  |  |  |  |  | 0.44 | 0.03 | 0.03 |  |  |  |
|  | Malignant neoplasms |  |  |  |  |  |  | 0.14 | 0.02 | 0.04 |  |  |  |
|  | Cardiovascular diseases |  |  |  |  |  |  | 0.40 | **0.78** | 0.82 |  |  |  |
|  | Respiratory diseases |  |  |  |  |  |  | 0.26 | 0.07 | -0.12 |  |  |  |
|  | Digestive system diseases |  |  |  |  |  |  | 0.12 | 0.11 | 0.05 |  |  |  |
|  | Pregnancy |  |  |  |  |  |  | 0.04 | 0.01 | 0.02 |  |  |  |
|  | Perinatal diseases |  |  |  |  |  |  | 0.14 | 0.07 | 0.12 |  |  |  |
|  | External causes |  |  |  |  |  |  | 0.24 | 0.00 | 0.04 |  |  |  |
|  | Other diseases |  |  |  |  |  |  | 0.77 | 0.00 | -0.06 |  |  |  |
|  | Total |  |  |  |  |  |  | 2.53 | 1.10 | 0.95 |  |  |  |
| Armenia | Infectious diseases |  |  |  |  |  |  |  | 0.07 | 0.06 | 0.08 |  |  |
|  | Malignant neoplasms |  |  |  |  |  |  |  | -0.15 | 0.31 | -0.16 |  |  |
|  | Cardiovascular diseases |  |  |  |  |  |  |  | -0.29 | 0.10 | 1.02 |  |  |
|  | Respiratory diseases |  |  |  |  |  |  |  | 0.82 | 0.20 | 0.22 |  |  |
|  | Digestive system diseases |  |  |  |  |  |  |  | 0.06 | 0.01 | 0.06 |  |  |
|  | Pregnancy |  |  |  |  |  |  |  | -0.01 | 0.02 | 0.00 |  |  |
|  | Perinatal diseases |  |  |  |  |  |  |  | -0.03 | 0.04 | -0.01 |  |  |
|  | External causes |  |  |  |  |  |  |  | -0.19 | 0.41 | 0.17 |  |  |
|  | Other diseases |  |  |  |  |  |  |  | -0.07 | -0.40 | 0.06 |  |  |
|  | Total |  |  |  |  |  |  |  | 0.22 | 0.76 | 1.45 |  |  |
| Australia | Infectious diseases | 0.29 | 0.10 | 0.06 | -0.06 | 0.06 | 0.04 | 0.01 | -0.01 | -0.01 | -0.02 |  |  |
|  | Malignant neoplasms | 0.00 | 0.07 | 0.01 | -0.08 | 0.04 | 0.08 | -0.14 | 0.06 | 0.10 | 0.26 |  |  |
|  | Cardiovascular diseases | 0.28 | **0.40** | -0.10 | 0.24 | 0.78 | 1.45 | 0.79 | 0.90 | 0.82 | 1.02 |  |  |
|  | Respiratory diseases | 0.14 | 0.05 | 0.03 | -0.02 | 0.11 | 0.18 | -0.02 | -0.02 | 0.03 | 0.12 |  |  |
|  | Digestive system diseases | 0.10 | 0.09 | 0.04 | 0.11 | 0.01 | 0.00 | 0.01 | 0.04 | 0.07 | 0.04 |  |  |
|  | Pregnancy | 0.06 | 0.02 | 0.03 | 0.01 | 0.02 | 0.00 | 0.00 | 0.00 | 0.00 | 0.00 |  |  |
|  | Perinatal diseases | 0.14 | 0.08 | 0.06 | 0.11 | 0.13 | 0.18 | 0.05 | 0.03 | 0.05 | 0.00 |  |  |
|  | External causes | -0.05 | -0.04 | -0.17 | 0.02 | 0.13 | 0.15 | 0.13 | 0.08 | 0.12 | -0.02 |  |  |
|  | Other diseases | 0.38 | 0.28 | 0.09 | 0.09 | 0.12 | 0.24 | -0.02 | 0.05 | -0.07 | 0.15 |  |  |
|  | Total | 1.33 | 1.05 | 0.05 | 0.42 | 1.39 | 2.32 | 0.82 | 1.12 | 1.11 | 1.54 |  |  |
| Austria | Infectious diseases |  | 0.23 | 0.10 | 0.07 | 0.06 | 0.03 | 0.02 | 0.00 | 0.00 | 0.00 | -0.03 | 0.00 |
|  | Malignant neoplasms |  | 0.05 | 0.03 | 0.10 | 0.13 | 0.15 | 0.09 | 0.13 | 0.17 | 0.24 | 0.18 | 0.13 |
|  | Cardiovascular diseases |  | **0.33** | 0.39 | -0.29 | 0.39 | 0.32 | 0.55 | 0.81 | 0.39 | 0.68 | 1.39 | 0.46 |
|  | Respiratory diseases |  | 0.19 | 0.19 | -0.09 | 0.23 | 0.20 | 0.11 | 0.09 | 0.08 | -0.04 | 0.01 | 0.07 |
|  | Digestive system diseases |  | 0.12 | 0.09 | 0.06 | 0.07 | 0.10 | 0.11 | 0.09 | 0.06 | 0.08 | 0.06 | 0.06 |
|  | Pregnancy |  | 0.02 | 0.04 | 0.02 | 0.02 | 0.01 | 0.00 | 0.00 | 0.00 | 0.00 | 0.00 | 0.00 |
|  | Perinatal diseases |  | 0.19 | 0.26 | 0.19 | 0.20 | 0.30 | 0.13 | 0.09 | 0.05 | 0.03 | -0.04 | 0.05 |
|  | External causes |  | 0.02 | 0.04 | -0.09 | 0.06 | 0.09 | 0.13 | 0.17 | 0.13 | 0.08 | 0.11 | 0.07 |
|  | Other diseases |  | 0.35 | -0.05 | 0.21 | 0.22 | 0.20 | 0.14 | 0.13 | 0.20 | 0.13 | -0.43 | -0.04 |
|  | Total |  | 1.51 | 1.10 | 0.18 | 1.38 | 1.38 | 1.30 | 1.51 | 1.09 | 1.20 | 1.25 | 0.78 |
| Azerbaijan | Infectious diseases |  |  |  |  |  |  |  | 0.14 | 0.02 | 0.20 |  |  |
|  | Malignant neoplasms |  |  |  |  |  |  |  | 0.35 | 0.10 | -0.06 |  |  |
|  | Cardiovascular diseases |  |  |  |  |  |  |  | 0.49 | -0.72 | -0.12 |  |  |
|  | Respiratory diseases |  |  |  |  |  |  |  | 0.25 | -0.02 | 0.84 |  |  |
|  | Digestive system diseases |  |  |  |  |  |  |  | 0.05 | -0.12 | -0.03 |  |  |
|  | Pregnancy |  |  |  |  |  |  |  | 0.04 | -0.02 | 0.02 |  |  |
|  | Perinatal diseases |  |  |  |  |  |  |  | 0.05 | -0.01 | 0.06 |  |  |
|  | External causes |  |  |  |  |  |  |  | 0.11 | -0.01 | 0.28 |  |  |
|  | Other diseases |  |  |  |  |  |  |  | -0.22 | -0.28 | 0.13 |  |  |
|  | Total |  |  |  |  |  |  |  | 1.25 | -1.06 | 1.32 |  |  |
| Belarus | Infectious diseases |  |  |  |  |  |  |  | 0.03 | 0.00 |  |  |  |
|  | Malignant neoplasms |  |  |  |  |  |  |  | -0.13 | 0.00 |  |  |  |
|  | Cardiovascular diseases |  |  |  |  |  |  |  | 0.87 | -0.50 |  |  |  |
|  | Respiratory diseases |  |  |  |  |  |  |  | 0.33 | 0.14 |  |  |  |
|  | Digestive system diseases |  |  |  |  |  |  |  | 0.01 | -0.02 |  |  |  |
|  | Pregnancy |  |  |  |  |  |  |  | 0.00 | 0.01 |  |  |  |
|  | Perinatal diseases |  |  |  |  |  |  |  | 0.02 | -0.06 |  |  |  |
|  | External causes |  |  |  |  |  |  |  | -0.13 | -0.31 |  |  |  |
|  | Other diseases |  |  |  |  |  |  |  | -0.85 | -0.70 |  |  |  |
|  | Total |  |  |  |  |  |  |  | 0.15 | -1.44 |  |  |  |

| **Country** | **Cause of death** | **1950-55** | **1955-60** | **1960-65** | **1965-70** | **1970-75** | **1975-80** | **1980-85** | **1985-90** | **1990-95** | **1995-2000** | **2000-05** | **2005-10** |
| --- | --- | --- | --- | --- | --- | --- | --- | --- | --- | --- | --- | --- | --- |
| Belgium | Infectious diseases |  | 0.21 | 0.10 | -0.04 | 0.08 | 0.02 | 0.02 | -0.02 | -0.04 |  |  |  |
|  | Malignant neoplasms |  | 0.02 | 0.03 | 0.09 | 0.08 | 0.02 | 0.09 | 0.14 | 0.09 |  |  |  |
|  | Cardiovascular diseases |  | **0.34** | -0.22 | 0.30 | 0.47 | 0.45 | 0.56 | 0.90 | 0.42 |  |  |  |
|  | Respiratory diseases |  | 0.25 | 0.09 | -0.04 | 0.11 | 0.13 | 0.00 | 0.02 | -0.06 |  |  |  |
|  | Digestive system diseases |  | 0.06 | 0.00 | 0.06 | 0.00 | 0.02 | 0.05 | 0.04 | 0.00 |  |  |  |
|  | Pregnancy |  | 0.03 | 0.02 | 0.01 | 0.01 | 0.00 | 0.00 | 0.00 | 0.00 |  |  |  |
|  | Perinatal diseases |  | 0.28 | 0.16 | 0.22 | 0.17 | 0.15 | 0.13 | 0.04 | 0.03 |  |  |  |
|  | External causes |  | -0.03 | -0.14 | -0.15 | 0.05 | -0.04 | 0.23 | 0.14 | 0.04 |  |  |  |
|  | Other diseases |  | 0.53 | 0.52 | -0.01 | 0.18 | 0.62 | 0.16 | 0.16 | 0.48 |  |  |  |
|  | Total |  | 1.68 | 0.56 | 0.44 | 1.14 | 1.38 | 1.24 | 1.42 | 0.96 |  |  |  |
| Bulgaria | Infectious diseases |  |  |  | 0.01 | 0.09 | 0.07 | 0.01 | 0.03 | -0.02 | 0.00 | 0.02 | 0.02 |
|  | Malignant neoplasms |  |  |  | 0.09 | 0.02 | -0.01 | -0.12 | 0.04 | -0.06 | 0.09 | -0.06 | 0.13 |
|  | Cardiovascular diseases |  |  |  | -0.47 | -0.09 | -0.01 | -0.22 | 0.11 | -0.09 | -0.01 | **0.60** | 0.75 |
|  | Respiratory diseases |  |  |  | -0.30 | 0.53 | 0.42 | 0.28 | 0.20 | 0.10 | 0.17 | 0.10 | 0.01 |
|  | Digestive system diseases |  |  |  | 0.08 | -0.01 | 0.00 | 0.00 | 0.02 | 0.00 | 0.05 | 0.00 | -0.01 |
|  | Pregnancy |  |  |  | 0.01 | 0.01 | 0.01 | 0.00 | 0.00 | 0.00 | 0.00 | 0.01 | 0.00 |
|  | Perinatal diseases |  |  |  | 0.47 | -0.03 | 0.05 | 0.07 | 0.03 | -0.02 | 0.01 | 0.02 | 0.02 |
|  | External causes |  |  |  | -0.05 | 0.02 | -0.07 | 0.01 | 0.02 | 0.01 | 0.08 | 0.10 | 0.12 |
|  | Other diseases |  |  |  | 0.05 | -0.07 | -0.03 | 0.29 | -0.06 | -0.01 | 0.07 | 0.34 | 0.18 |
|  | Total |  |  |  | -0.12 | 0.47 | 0.43 | 0.33 | 0.40 | -0.09 | 0.45 | 1.12 | 1.24 |
| Canada | Infectious diseases | 0.49 | 0.14 | 0.06 | -0.01 | 0.03 | 0.05 | -0.01 | -0.02 | -0.02 | -0.01 | -0.04 |  |
|  | Malignant neoplasms | 0.08 | 0.06 | 0.06 | 0.03 | 0.07 | 0.01 | -0.06 | 0.01 | 0.08 | 0.12 | 0.10 |  |
|  | Cardiovascular diseases | **0.50** | 0.30 | 0.62 | 0.70 | 0.60 | 0.89 | 0.81 | 0.73 | 0.49 | 0.58 | 0.59 |  |
|  | Respiratory diseases | 0.30 | 0.06 | 0.16 | -0.03 | 0.13 | 0.15 | -0.06 | -0.05 | -0.01 | 0.06 | 0.04 |  |
|  | Digestive system diseases | 0.13 | 0.02 | 0.05 | 0.09 | 0.01 | 0.00 | 0.07 | 0.04 | 0.04 | 0.02 | 0.00 |  |
|  | Pregnancy | 0.05 | 0.04 | 0.04 | 0.02 | 0.02 | 0.00 | 0.00 | 0.00 | 0.00 | 0.00 | 0.00 |  |
|  | Perinatal diseases | 0.20 | 0.08 | 0.09 | 0.25 | 0.19 | 0.15 | 0.07 | 0.04 | 0.00 | 0.01 | -0.04 |  |
|  | External causes | -0.03 | 0.04 | -0.11 | -0.08 | 0.00 | 0.16 | 0.23 | 0.08 | 0.11 | 0.05 | 0.00 |  |
|  | Other diseases | 0.47 | 0.30 | 0.18 | 0.17 | 0.07 | 0.13 | -0.08 | -0.11 | -0.01 | -0.08 | -0.01 |  |
|  | Total | 2.19 | 1.02 | 1.14 | 1.15 | 1.13 | 1.54 | 0.97 | 0.71 | 0.68 | 0.75 | 0.63 |  |
| Chile | Infectious diseases |  | 0.08 | 0.40 | -0.38 | 0.72 | 0.75 | 0.29 | 0.11 |  |  |  |  |
|  | Malignant neoplasms |  | -0.09 | -0.06 | 0.04 | 0.36 | 0.15 | 0.09 | 0.07 |  |  |  |  |
|  | Cardiovascular diseases |  | 0.05 | -0.13 | 0.09 | 1.19 | 0.05 | 0.29 | 0.57 |  |  |  |  |
|  | Respiratory diseases |  | -0.08 | 1.22 | 0.32 | 1.32 | 1.09 | 0.13 | 0.13 |  |  |  |  |
|  | Digestive system diseases |  | -0.32 | -0.12 | 1.21 | 0.28 | 0.08 | 0.24 | 0.10 |  |  |  |  |
|  | Pregnancy |  | 0.03 | 0.06 | 0.22 | 0.10 | 0.09 | 0.04 | 0.01 |  |  |  |  |
|  | Perinatal diseases |  | 0.26 | 0.51 | 1.32 | -0.15 | 0.48 | 0.32 | 0.09 |  |  |  |  |
|  | External causes |  | -0.07 | -0.04 | -0.09 | 0.15 | -0.03 | 0.08 | 0.08 |  |  |  |  |
|  | Other diseases |  | 0.73 | 0.67 | 0.36 | -0.14 | 0.58 | 0.60 | 0.46 |  |  |  |  |
|  | Total |  | 0.59 | 2.51 | 3.08 | 3.84 | 3.25 | 2.08 | 1.62 |  |  |  |  |
| China,select rural areas | Infectious diseases |  |  |  |  |  |  |  |  | 0.21 | 0.13 |  |  |
|  | Malignant neoplasms |  |  |  |  |  |  |  |  | 0.05 | -0.09 |  |  |
|  | Cardiovascular diseases |  |  |  |  |  |  |  |  | 0.37 | -0.25 |  |  |
|  | Respiratory diseases |  |  |  |  |  |  |  |  | 0.27 | 0.65 |  |  |
|  | Digestive system diseases |  |  |  |  |  |  |  |  | 0.14 | 0.14 |  |  |
|  | Pregnancy |  |  |  |  |  |  |  |  | 0.02 | 0.01 |  |  |
|  | Perinatal diseases |  |  |  |  |  |  |  |  | -0.13 | 0.15 |  |  |
|  | External causes |  |  |  |  |  |  |  |  | 0.15 | 0.29 |  |  |
|  | Other diseases |  |  |  |  |  |  |  |  | 0.09 | 0.02 |  |  |
|  | Total |  |  |  |  |  |  |  |  | 1.16 | 1.04 |  |  |
| China,select urban | Infectious diseases |  |  |  |  |  |  |  |  | 0.15 | 0.09 |  |  |
| and rural areas | Malignant neoplasms |  |  |  |  |  |  |  |  | 0.11 | -0.09 |  |  |
|  | Cardiovascular diseases |  |  |  |  |  |  |  |  | 0.34 | 0.08 |  |  |
|  | Respiratory diseases |  |  |  |  |  |  |  |  | 0.27 | 0.55 |  |  |
|  | Digestive system diseases |  |  |  |  |  |  |  |  | 0.13 | 0.10 |  |  |
|  | Pregnancy |  |  |  |  |  |  |  |  | 0.01 | 0.01 |  |  |
|  | Perinatal diseases |  |  |  |  |  |  |  |  | -0.04 | 0.14 |  |  |
|  | External causes |  |  |  |  |  |  |  |  | 0.16 | 0.19 |  |  |
|  | Other diseases |  |  |  |  |  |  |  |  | 0.03 | 0.12 |  |  |
|  | Total |  |  |  |  |  |  |  |  | 1.16 | 1.17 |  |  |
| China,select urban areas | Infectious diseases |  |  |  |  |  |  |  |  | 0.10 | 0.05 |  |  |
|  | Malignant neoplasms |  |  |  |  |  |  |  |  | 0.15 | -0.09 |  |  |
|  | Cardiovascular diseases |  |  |  |  |  |  |  |  | 0.33 | 0.32 |  |  |
|  | Respiratory diseases |  |  |  |  |  |  |  |  | 0.24 | 0.46 |  |  |
|  | Digestive system diseases |  |  |  |  |  |  |  |  | 0.12 | 0.07 |  |  |
|  | Pregnancy |  |  |  |  |  |  |  |  | 0.01 | 0.00 |  |  |
|  | Perinatal diseases |  |  |  |  |  |  |  |  | 0.07 | 0.12 |  |  |
|  | External causes |  |  |  |  |  |  |  |  | 0.16 | 0.11 |  |  |
|  | Other diseases |  |  |  |  |  |  |  |  | -0.03 | 0.22 |  |  |
|  | Total |  |  |  |  |  |  |  |  | 1.16 | 1.25 |  |  |

| **Country** | **Cause of death** | **1950-55** | **1955-60** | **1960-65** | **1965-70** | **1970-75** | **1975-80** | **1980-85** | **1985-90** | **1990-95** | **1995-2000** | **2000-05** | **2005-10** |
| --- | --- | --- | --- | --- | --- | --- | --- | --- | --- | --- | --- | --- | --- |
| Costa Rica | Infectious diseases |  |  |  | -1.24 | 1.72 | 0.70 | 0.10 | 0.07 | 0.06 |  |  |  |
|  | Malignant neoplasms |  |  |  | 0.29 | 0.12 | 0.13 | -0.34 | 0.43 | 0.01 |  |  |  |
|  | Cardiovascular diseases |  |  |  | -0.45 | 0.93 | 0.20 | -0.52 | 0.66 | -0.12 |  |  |  |
|  | Respiratory diseases |  |  |  | -0.26 | 0.92 | 0.40 | -0.15 | 0.18 | 0.05 |  |  |  |
|  | Digestive system diseases |  |  |  | 1.93 | 0.12 | 0.06 | 0.04 | 0.01 | -0.11 |  |  |  |
|  | Pregnancy |  |  |  | 0.14 | 0.13 | 0.05 | 0.00 | 0.02 | 0.00 |  |  |  |
|  | Perinatal diseases |  |  |  | 0.58 | 0.21 | 0.11 | -0.05 | 0.14 | 0.00 |  |  |  |
|  | External causes |  |  |  | 0.00 | -0.16 | 0.09 | 0.05 | 0.02 | -0.09 |  |  |  |
|  | Other diseases |  |  |  | 0.57 | 0.64 | 0.45 | 0.65 | 0.19 | 0.27 |  |  |  |
|  | Total |  |  |  | 1.57 | 4.63 | 2.19 | -0.21 | 1.71 | 0.06 |  |  |  |
| Cuba | Infectious diseases |  |  |  |  | 0.71 | 0.16 | 0.09 | 0.04 | -0.03 |  |  |  |
|  | Malignant neoplasms |  |  |  |  | 0.02 | 0.12 | -0.06 | 0.07 | 0.02 |  |  |  |
|  | Cardiovascular diseases |  |  |  |  | 0.21 | 0.30 | 0.18 | 0.57 | 0.07 |  |  |  |
|  | Respiratory diseases |  |  |  |  | 0.17 | 0.17 | 0.12 | 0.31 | -0.18 |  |  |  |
|  | Digestive system diseases |  |  |  |  | 0.10 | 0.00 | -0.02 | 0.03 | 0.02 |  |  |  |
|  | Pregnancy |  |  |  |  | 0.06 | 0.03 | 0.00 | 0.01 | 0.01 |  |  |  |
|  | Perinatal diseases |  |  |  |  | 0.19 | 0.16 | 0.19 | 0.14 | 0.04 |  |  |  |
|  | External causes |  |  |  |  | -0.08 | -0.17 | 0.04 | 0.00 | 0.08 |  |  |  |
|  | Other diseases |  |  |  |  | 0.31 | 0.05 | -0.01 | -0.16 | -0.02 |  |  |  |
|  | Total |  |  |  |  | 1.68 | 0.82 | 0.53 | 1.01 | 0.00 |  |  |  |
| Czechoslovakia, Former | Infectious diseases |  | 0.41 | 0.21 | 0.10 | 0.05 | 0.03 | 0.02 | 0.01 |  |  |  |  |
|  | Malignant neoplasms |  | 0.03 | -0.01 | 0.04 | 0.07 | -0.02 | -0.03 | -0.04 |  |  |  |  |
|  | Cardiovascular diseases |  | 0.51 | 0.21 | -0.49 | 0.22 | 0.03 | -0.08 | 0.35 |  |  |  |  |
|  | Respiratory diseases |  | 0.20 | 0.21 | -0.10 | 0.09 | 0.14 | 0.12 | 0.22 |  |  |  |  |
|  | Digestive system diseases |  | 0.14 | 0.03 | 0.01 | 0.01 | 0.02 | 0.07 | 0.00 |  |  |  |  |
|  | Pregnancy |  | 0.04 | 0.01 | 0.01 | 0.00 | 0.01 | 0.00 | 0.00 |  |  |  |  |
|  | Perinatal diseases |  | 0.26 | -0.12 | 0.04 | -0.02 | 0.15 | 0.13 | 0.07 |  |  |  |  |
|  | External causes |  | -0.03 | 0.00 | -0.09 | 0.06 | 0.07 | 0.03 | 0.02 |  |  |  |  |
|  | Other diseases |  | 0.57 | 0.04 | 0.19 | 0.21 | -0.08 | 0.18 | 0.17 |  |  |  |  |
|  | Total |  | 2.12 | 0.58 | -0.28 | 0.69 | 0.33 | 0.43 | 0.79 |  |  |  |  |
| Czech Republic | Infectious diseases |  |  |  |  |  |  |  |  | 0.01 | 0.01 | -0.01 | -0.07 |
|  | Malignant neoplasms |  |  |  |  |  |  |  |  | 0.11 | 0.14 | 0.23 | 0.35 |
|  | Cardiovascular diseases |  |  |  |  |  |  |  |  | **0.73** | 0.90 | 0.75 | 0.73 |
|  | Respiratory diseases |  |  |  |  |  |  |  |  | 0.05 | 0.03 | -0.01 | 0.00 |
|  | Digestive system diseases |  |  |  |  |  |  |  |  | 0.04 | 0.02 | -0.01 | 0.03 |
|  | Pregnancy |  |  |  |  |  |  |  |  | 0.01 | 0.00 | 0.00 | 0.00 |
|  | Perinatal diseases |  |  |  |  |  |  |  |  | 0.09 | 0.11 | 0.02 | 0.02 |
|  | External causes |  |  |  |  |  |  |  |  | 0.04 | 0.21 | 0.09 | 0.11 |
|  | Other diseases |  |  |  |  |  |  |  |  | 0.28 | 0.10 | -0.02 | 0.00 |
|  | Total |  |  |  |  |  |  |  |  | 1.37 | 1.52 | 1.04 | 1.15 |
| Slovakia | Infectious diseases |  |  |  |  |  |  |  |  |  | 0.00 | -0.01 | -0.03 |
|  | Malignant neoplasms |  |  |  |  |  |  |  |  |  | -0.04 | 0.17 | 0.09 |
|  | Cardiovascular diseases |  |  |  |  |  |  |  |  |  | **0.37** | 0.43 | 0.60 |
|  | Respiratory diseases |  |  |  |  |  |  |  |  |  | 0.30 | -0.05 | 0.04 |
|  | Digestive system diseases |  |  |  |  |  |  |  |  |  | -0.05 | -0.04 | 0.03 |
|  | Pregnancy |  |  |  |  |  |  |  |  |  | 0.00 | 0.00 | 0.17 |
|  | Perinatal diseases |  |  |  |  |  |  |  |  |  | 0.15 | 0.01 | 0.06 |
|  | External causes |  |  |  |  |  |  |  |  |  | 0.17 | 0.01 | 0.07 |
|  | Other diseases |  |  |  |  |  |  |  |  |  | -0.02 | 0.03 | 0.04 |
|  | Total |  |  |  |  |  |  |  |  |  | 0.88 | 0.55 | 0.90 |
| Denmark | Infectious diseases |  | 0.10 | 0.03 | -0.01 | 0.04 | 0.01 | 0.00 | -0.02 | -0.01 | 0.02 | -0.05 |  |
|  | Malignant neoplasms |  | 0.01 | 0.07 | 0.08 | 0.09 | -0.10 | -0.03 | -0.02 | 0.00 | 0.17 | 0.32 |  |
|  | Cardiovascular diseases |  | **0.41** | 0.03 | 0.79 | 0.35 | 0.53 | 0.34 | 0.30 | 0.51 | 0.63 | 0.61 |  |
|  | Respiratory diseases |  | -0.01 | 0.14 | -0.04 | -0.10 | 0.05 | 0.00 | -0.03 | -0.17 | 0.00 | 0.13 |  |
|  | Digestive system diseases |  | 0.11 | 0.07 | 0.14 | 0.06 | 0.01 | -0.03 | -0.03 | -0.09 | 0.00 | 0.03 |  |
|  | Pregnancy |  | 0.02 | 0.02 | 0.01 | 0.01 | 0.00 | 0.00 | 0.00 | 0.00 | 0.61 | 0.96 |  |
|  | Perinatal diseases |  | 0.10 | 0.15 | 0.20 | 0.17 | 0.13 | 0.01 | 0.01 | 0.01 | 0.02 | -0.01 |  |
|  | External causes |  | 0.05 | -0.06 | -0.10 | 0.17 | -0.09 | 0.03 | 0.16 | 0.18 | 0.10 | 0.16 |  |
|  | Other diseases |  | -0.05 | 0.20 | 0.13 | 0.17 | -0.07 | -0.06 | -0.09 | -0.26 | 0.22 | 0.08 |  |
|  | Total |  | 0.74 | 0.65 | 1.21 | 0.96 | 0.47 | 0.28 | 0.26 | 0.16 | 1.16 | 1.26 |  |
| Estonia | Infectious diseases |  |  |  |  |  |  |  | 0.03 | -0.04 | 0.01 | 0.02 | 0.01 |
|  | Malignant neoplasms |  |  |  |  |  |  |  | -0.13 | 0.02 | 0.06 | 0.20 | 0.17 |
|  | Cardiovascular diseases |  |  |  |  |  |  |  | **0.60** | 0.09 | 1.08 | 1.08 | 1.29 |
|  | Respiratory diseases |  |  |  |  |  |  |  | 0.06 | -0.03 | 0.08 | 0.07 | 0.02 |
|  | Digestive system diseases |  |  |  |  |  |  |  | -0.02 | -0.08 | -0.09 | 0.01 | 0.13 |
|  | Pregnancy |  |  |  |  |  |  |  | 0.01 | 0.01 | 0.01 | 0.00 | 0.55 |
|  | Perinatal diseases |  |  |  |  |  |  |  | 0.09 | -0.07 | 0.14 | 0.13 | 0.09 |
|  | External causes |  |  |  |  |  |  |  | -0.16 | -0.61 | 0.55 | 0.39 | 0.37 |
|  | Other diseases |  |  |  |  |  |  |  | -0.18 | 0.03 | 0.14 | 0.04 | 0.52 |
|  | Total |  |  |  |  |  |  |  | 0.31 | -0.67 | 1.98 | 1.95 | 2.61 |

| **Country** | **Cause of death** | **1950-55** | **1955-60** | **1960-65** | **1965-70** | **1970-75** | **1975-80** | **1980-85** | **1985-90** | **1990-95** | **1995-2000** | **2000-05** | **2005-10** |
| --- | --- | --- | --- | --- | --- | --- | --- | --- | --- | --- | --- | --- | --- |
| Finland | Infectious diseases |  | 0.41 | 0.21 | 0.04 | 0.02 | 0.03 | 0.02 | 0.02 | 0.01 | 0.00 | 0.03 | 0.00 |
|  | Malignant neoplasms |  | 0.07 | 0.11 | 0.09 | 0.13 | 0.06 | 0.08 | 0.07 | 0.16 | 0.07 | 0.15 | 0.07 |
|  | Cardiovascular diseases |  | 0.37 | -0.14 | **0.52** | 1.14 | 0.94 | 0.60 | 0.63 | 0.81 | 0.87 | 0.75 | 0.54 |
|  | Respiratory diseases |  | 0.20 | 0.22 | -0.12 | 0.20 | 0.20 | 0.09 | 0.02 | 0.03 | 0.04 | 0.33 | 0.09 |
|  | Digestive system diseases |  | 0.06 | 0.09 | 0.10 | 0.07 | 0.07 | 0.00 | -0.08 | 0.03 | 0.00 | -0.03 | 0.04 |
|  | Pregnancy |  | 0.06 | 0.04 | 0.03 | 0.00 | 0.00 | 0.00 | 0.00 | 0.00 | 0.00 | 0.00 | 0.00 |
|  | Perinatal diseases |  | 0.17 | 0.10 | 0.17 | 0.19 | 0.13 | 0.04 | -0.03 | 0.05 | 0.04 | -0.01 | 0.03 |
|  | External causes |  | -0.02 | -0.04 | -0.04 | 0.10 | 0.18 | 0.02 | -0.16 | 0.15 | 0.06 | -0.01 | 0.15 |
|  | Other diseases |  | 0.15 | 0.10 | 0.31 | 0.24 | 0.17 | 0.03 | -0.13 | 0.06 | -0.06 | 0.07 | -0.04 |
|  | Total |  | 1.47 | 0.68 | 1.09 | 2.08 | 1.78 | 0.88 | 0.34 | 1.30 | 1.03 | 1.28 | 0.88 |
| France | Infectious diseases |  | 0.23 | 0.14 | 0.03 | 0.01 | 0.08 | 0.02 | -0.02 | -0.04 | 0.05 | 0.01 |  |
|  | Malignant neoplasms |  | 0.02 | 0.05 | 0.14 | 0.02 | 0.11 | 0.07 | 0.10 | 0.09 | 0.03 | 0.10 |  |
|  | Cardiovascular diseases |  | **0.50** | 0.35 | 0.14 | 0.30 | 0.57 | 0.43 | 0.81 | 0.47 | 0.25 | 0.46 |  |
|  | Respiratory diseases |  | 0.27 | 0.26 | -0.03 | 0.18 | 0.15 | 0.02 | 0.05 | 0.02 | 0.05 | 0.11 |  |
|  | Digestive system diseases |  | 0.13 | -0.01 | 0.01 | 0.02 | 0.06 | 0.15 | 0.14 | 0.07 | 0.06 | 0.07 |  |
|  | Pregnancy |  | 0.01 | 0.02 | 0.01 | 0.01 | 0.01 | 0.00 | 0.00 | 0.00 | 0.00 | 0.00 |  |
|  | Perinatal diseases |  | 0.31 | 0.16 | 0.20 | 0.18 | 0.09 | 0.04 | 0.02 | 0.00 | -0.01 | 0.01 |  |
|  | External causes |  | -0.03 | -0.09 | -0.13 | 0.06 | 0.01 | 0.11 | 0.15 | 0.16 | 0.09 | 0.19 |  |
|  | Other diseases |  | 0.76 | 0.33 | 0.61 | 0.43 | 0.34 | 0.16 | 0.32 | 0.22 | -0.28 | 0.08 |  |
|  | Total |  | 2.22 | 1.21 | 0.98 | 1.21 | 1.42 | 1.00 | 1.58 | 0.99 | 0.25 | 1.05 |  |
| Germany, | Infectious diseases |  | 0.16 | 0.11 | -0.01 | 0.02 | 0.05 | 0.02 | -0.01 |  |  |  |  |
| Former Federal Republic | Malignant neoplasms |  | 0.02 | -0.01 | 0.09 | 0.10 | 0.14 | 0.10 | 0.03 |  |  |  |  |
|  | Cardiovascular diseases |  | 0.34 | 0.29 | -0.10 | **0.37** | 0.36 | 0.64 | 0.56 |  |  |  |  |
|  | Respiratory diseases |  | 0.19 | 0.31 | -0.13 | 0.19 | 0.14 | 0.02 | 0.06 |  |  |  |  |
|  | Digestive system diseases |  | 0.00 | 0.06 | 0.07 | 0.05 | 0.09 | 0.13 | 0.02 |  |  |  |  |
|  | Pregnancy |  | 0.03 | 0.04 | 0.03 | 0.02 | 0.01 | 0.01 | 0.00 |  |  |  |  |
|  | Perinatal diseases |  | 0.33 | 0.33 | 0.11 | 0.23 | 0.34 | 0.14 | 0.08 |  |  |  |  |
|  | External causes |  | 0.02 | -0.07 | -0.11 | 0.09 | 0.17 | 0.21 | 0.15 |  |  |  |  |
|  | Other diseases |  | 0.31 | 0.33 | 0.09 | 0.17 | 0.53 | 0.25 | 0.02 |  |  |  |  |
|  | Total |  | 1.39 | 1.39 | 0.03 | 1.25 | 1.82 | 1.52 | 0.92 |  |  |  |  |
| Germany | Infectious diseases |  |  |  |  |  |  |  |  | -0.01 | -0.02 | -0.01 | -0.02 |
|  | Malignant neoplasms |  |  |  |  |  |  |  |  | 0.11 | 0.25 | 0.21 | 0.12 |
|  | Cardiovascular diseases |  |  |  |  |  |  |  |  | **0.67** | 0.67 | 0.59 | 0.73 |
|  | Respiratory diseases |  |  |  |  |  |  |  |  | 0.05 | 0.04 | -0.03 | 0.00 |
|  | Digestive system diseases |  |  |  |  |  |  |  |  | 0.04 | 0.05 | 0.02 | 0.06 |
|  | Pregnancy |  |  |  |  |  |  |  |  | 0.00 | 0.00 | 0.00 | 0.00 |
|  | Perinatal diseases |  |  |  |  |  |  |  |  | 0.02 | 0.01 | 0.00 | 0.02 |
|  | External causes |  |  |  |  |  |  |  |  | 0.14 | 0.10 | 0.08 | 0.04 |
|  | Other diseases |  |  |  |  |  |  |  |  | 0.15 | 0.20 | 0.01 | -0.14 |
|  | Total |  |  |  |  |  |  |  |  | 1.18 | 1.30 | 0.86 | 0.81 |
| Greece | Infectious diseases |  |  |  | -0.09 | 0.16 | 0.18 | 0.04 | 0.02 | -0.02 | 0.02 | 0.00 | -0.02 |
|  | Malignant neoplasms |  |  |  | 0.01 | -0.11 | -0.03 | 0.05 | 0.06 | 0.00 | 0.06 | 0.08 | 0.07 |
|  | Cardiovascular diseases |  |  |  | 0.00 | 0.01 | -0.17 | -0.05 | 0.04 | **0.37** | 0.32 | 0.61 | 0.76 |
|  | Respiratory diseases |  |  |  | 0.00 | 0.34 | 0.35 | 0.17 | 0.08 | 0.02 | -0.09 | 0.00 | -0.04 |
|  | Digestive system diseases |  |  |  | 0.16 | 0.12 | 0.01 | 0.09 | 0.10 | 0.01 | 0.02 | 0.03 | 0.02 |
|  | Pregnancy |  |  |  | 0.02 | 0.01 | 0.01 | 0.01 | 0.00 | 0.00 | 0.00 | 0.00 | 0.00 |
|  | Perinatal diseases |  |  |  | 0.46 | 0.13 | 0.15 | 0.19 | 0.18 | 0.03 | 0.09 | 0.06 | 0.01 |
|  | External causes |  |  |  | 0.03 | -0.05 | 0.01 | 0.03 | 0.09 | 0.07 | 0.04 | 0.09 | 0.08 |
|  | Other diseases |  |  |  | 0.78 | 0.56 | 0.47 | 0.33 | 0.39 | 0.19 | 0.21 | 0.06 | 0.06 |
|  | Total |  |  |  | 1.37 | 1.16 | 0.97 | 0.86 | 0.96 | 0.67 | 0.67 | 0.93 | 0.95 |
| Hong Kong SAR | Infectious diseases |  |  | 0.79 | 0.40 | 0.30 | 0.07 | 0.06 | -0.10 | 0.01 | 0.19 | -0.02 | 0.04 |
|  | Malignant neoplasms |  |  | -0.24 | -0.01 | -0.02 | 0.07 | -0.02 | 0.07 | 0.20 | 0.14 | 0.31 | 0.20 |
|  | Cardiovascular diseases |  |  | -0.13 | 0.41 | 0.15 | 0.22 | **0.89** | 0.23 | 0.57 | 0.41 | 0.49 | 0.57 |
|  | Respiratory diseases |  |  | 1.29 | -0.33 | 0.15 | 0.34 | 0.53 | 0.01 | 0.09 | 0.49 | 0.22 | 0.03 |
|  | Digestive system diseases |  |  | 0.57 | 0.08 | 0.08 | 0.11 | 0.08 | -0.01 | 0.03 | 0.07 | 0.06 | 0.06 |
|  | Pregnancy |  |  | 0.03 | 0.05 | 0.01 | 0.01 | 0.00 | 0.00 | 0.00 | 0.00 | 0.00 | 0.00 |
|  | Perinatal diseases |  |  | 0.22 | 0.39 | 0.12 | 0.04 | 0.12 | 0.07 | 0.07 | 0.09 | -0.01 | 0.04 |
|  | External causes |  |  | 0.01 | 0.08 | -0.09 | 0.04 | 0.24 | 0.08 | 0.09 | 0.02 | 0.02 | 0.12 |
|  | Other diseases |  |  | 0.74 | 0.06 | 0.48 | 0.44 | 0.87 | 0.12 | 0.42 | -0.07 | 0.06 | 0.08 |
|  | Total |  |  | 3.28 | 1.12 | 1.17 | 1.33 | 2.78 | 0.48 | 1.48 | 1.35 | 1.12 | 1.14 |
| Hungary | Infectious diseases |  | 0.34 | 0.21 | 0.01 | 0.11 | 0.09 | 0.03 | 0.00 | 0.02 | 0.01 | 0.02 | -0.01 |
|  | Malignant neoplasms |  | -0.05 | 0.00 | -0.04 | -0.11 | 0.05 | -0.01 | -0.10 | -0.07 | 0.02 | 0.33 | 0.02 |
|  | Cardiovascular diseases |  | -0.03 | 0.01 | -0.15 | **0.33** | -0.07 | 0.12 | 0.41 | 0.43 | 0.51 | 0.74 | 0.86 |
|  | Respiratory diseases |  | 0.30 | 0.77 | -0.01 | 0.12 | -0.03 | 0.08 | 0.10 | 0.03 | 0.08 | -0.02 | -0.01 |
|  | Digestive system diseases |  | 0.19 | 0.16 | 0.11 | -0.06 | -0.10 | -0.06 | -0.10 | -0.11 | 0.13 | 0.16 | 0.15 |
|  | Pregnancy |  | 0.05 | 0.02 | 0.00 | 0.01 | 0.01 | 0.00 | 0.00 | 0.00 | 0.00 | 0.00 | 0.00 |
|  | Perinatal diseases |  | 0.19 | 0.04 | 0.08 | 0.01 | 0.51 | 0.08 | 0.15 | 0.20 | 0.07 | 0.12 | 0.01 |
|  | External causes |  | 0.07 | -0.07 | -0.11 | -0.06 | -0.12 | 0.02 | 0.00 | 0.22 | 0.17 | 0.17 | 0.14 |
|  | Other diseases |  | 0.70 | 0.47 | 0.26 | 0.03 | 0.07 | 0.04 | 0.17 | 0.11 | 0.09 | -0.03 | 0.01 |
|  | Total |  | 1.75 | 1.61 | 0.14 | 0.38 | 0.42 | 0.31 | 0.64 | 0.83 | 1.10 | 1.49 | 1.17 |

| **Country** | **Cause of death** | **1950-55** | **1955-60** | **1960-65** | **1965-70** | **1970-75** | **1975-80** | **1980-85** | **1985-90** | **1990-95** | **1995-2000** | **2000-05** | **2005-10** |
| --- | --- | --- | --- | --- | --- | --- | --- | --- | --- | --- | --- | --- | --- |
| Ireland | Infectious diseases | 1.44 | 0.35 | 0.19 | -0.01 | 0.08 | 0.04 | 0.06 | 0.01 | -0.02 | 0.00 | 0.04 |  |
|  | Malignant neoplasms | -0.05 | -0.01 | -0.05 | -0.17 | -0.09 | 0.16 | -0.03 | -0.03 | 0.19 | 0.11 | 0.22 |  |
|  | Cardiovascular diseases | 0.13 | **0.38** | 0.27 | 0.37 | 0.26 | 0.53 | 0.63 | 0.84 | 0.49 | 0.74 | 1.25 |  |
|  | Respiratory diseases | 0.70 | -0.02 | -0.04 | -0.17 | 0.18 | 0.23 | 0.14 | 0.10 | 0.02 | 0.01 | 0.41 |  |
|  | Digestive system diseases | 0.15 | 0.08 | 0.08 | 0.10 | -0.01 | -0.01 | 0.03 | 0.04 | -0.02 | -0.02 | 0.05 |  |
|  | Pregnancy | 0.06 | 0.06 | 0.03 | 0.00 | 0.03 | 0.01 | 0.00 | 0.00 | 0.00 | 0.00 | 0.00 |  |
|  | Perinatal diseases | 0.25 | 0.18 | 0.16 | 0.31 | 0.07 | 0.18 | 0.05 | 0.03 | 0.01 | 0.00 | 0.03 |  |
|  | External causes | 0.02 | 0.00 | -0.04 | -0.14 | -0.03 | 0.01 | 0.14 | 0.04 | 0.06 | -0.01 | 0.10 |  |
|  | Other diseases | 0.80 | 0.56 | 0.39 | 0.57 | 0.17 | 0.10 | 0.15 | 0.06 | 0.15 | -0.06 | 0.12 |  |
|  | Total | 3.50 | 1.58 | 0.98 | 0.86 | 0.66 | 1.25 | 1.16 | 1.11 | 0.89 | 0.77 | 2.24 |  |
| Israel | Infectious diseases |  |  |  |  |  | 0.14 | 0.00 | 0.06 | 0.05 | -0.09 | 0.01 | -0.01 |
|  | Malignant neoplasms |  |  |  |  |  | 0.08 | 0.23 | -0.04 | -0.09 | 0.24 | 0.22 | 0.19 |
|  | Cardiovascular diseases |  |  |  |  |  | **0.58** | 0.99 | 0.81 | 0.52 | 1.43 | 0.57 | 0.72 |
|  | Respiratory diseases |  |  |  |  |  | 0.07 | 0.03 | 0.08 | 0.27 | -0.14 | 0.05 | 0.12 |
|  | Digestive system diseases |  |  |  |  |  | 0.01 | 0.04 | 0.03 | -0.01 | 0.05 | 0.02 | 0.04 |
|  | Pregnancy |  |  |  |  |  | 0.02 | 0.00 | 0.00 | 0.00 | 0.00 | 0.00 | 0.00 |
|  | Perinatal diseases |  |  |  |  |  | 0.13 | 0.12 | 0.04 | 0.08 | 0.05 | 0.04 | 0.00 |
|  | External causes |  |  |  |  |  | 0.19 | -0.08 | 0.12 | 0.14 | 0.12 | 0.04 | 0.11 |
|  | Other diseases |  |  |  |  |  | -0.06 | -0.11 | 0.31 | 0.38 | -0.32 | 0.17 | 0.36 |
|  | Total |  |  |  |  |  | 1.16 | 1.21 | 1.40 | 1.34 | 1.34 | 1.13 | 1.54 |
| Italy | Infectious diseases |  | 0.24 | 0.20 | -0.07 | 0.19 | 0.10 | 0.02 | -0.02 | -0.08 | 0.08 |  |  |
|  | Malignant neoplasms |  | -0.05 | -0.03 | 0.00 | 0.07 | 0.05 | -0.01 | 0.05 | 0.13 | 0.19 |  |  |
|  | Cardiovascular diseases |  | **0.53** | 0.20 | 0.45 | 0.39 | 0.77 | 0.65 | 0.90 | 0.43 | 0.63 |  |  |
|  | Respiratory diseases |  | 0.29 | 0.30 | -0.07 | 0.45 | 0.24 | 0.13 | 0.13 | 0.07 | 0.01 |  |  |
|  | Digestive system diseases |  | 0.19 | 0.19 | 0.23 | 0.05 | 0.07 | 0.05 | 0.08 | 0.06 | 0.08 |  |  |
|  | Pregnancy |  | 0.01 | 0.03 | 0.03 | 0.03 | 0.02 | 0.00 | 0.00 | 0.00 | 0.00 |  |  |
|  | Perinatal diseases |  | 0.29 | 0.19 | 0.33 | 0.22 | 0.25 | 0.16 | 0.09 | 0.10 | 0.06 |  |  |
|  | External causes |  | -0.02 | -0.02 | -0.04 | -0.01 | 0.02 | 0.10 | 0.05 | 0.05 | 0.07 |  |  |
|  | Other diseases |  | 0.51 | 0.23 | 0.20 | 0.29 | 0.15 | 0.03 | 0.11 | 0.12 | 0.10 |  |  |
|  | Total |  | 1.99 | 1.28 | 1.06 | 1.68 | 1.66 | 1.13 | 1.40 | 0.89 | 1.22 |  |  |
| Japan | Infectious diseases | 2.36 | 0.91 | 0.45 | 0.13 | 0.16 | 0.11 | 0.03 | 0.02 | -0.04 | 0.00 | 0.00 | 0.02 |
|  | Malignant neoplasms | -0.01 | -0.04 | 0.05 | 0.08 | 0.12 | 0.09 | 0.12 | 0.11 | 0.02 | 0.09 | 0.18 | 0.19 |
|  | Cardiovascular diseases | 0.15 | -0.02 | 0.23 | **0.41** | 0.59 | 0.96 | 1.05 | 0.85 | 0.90 | 0.79 | 0.47 | 0.71 |
|  | Respiratory diseases | 0.75 | 0.22 | 0.44 | -0.02 | 0.15 | 0.22 | 0.01 | -0.05 | 0.01 | 0.14 | 0.08 | 0.27 |
|  | Digestive system diseases | 1.05 | 0.51 | 0.40 | 0.30 | 0.15 | 0.13 | 0.09 | 0.08 | 0.07 | 0.08 | 0.03 | 0.05 |
|  | Pregnancy | 0.06 | 0.06 | 0.04 | 0.03 | 0.02 | 0.01 | 0.01 | 0.01 | 0.00 | 0.00 | 0.00 | 0.00 |
|  | Perinatal diseases | 0.33 | 0.42 | 0.34 | 0.27 | 0.11 | 0.08 | 0.09 | 0.06 | 0.02 | 0.02 | 0.01 | 0.01 |
|  | External causes | -0.06 | -0.01 | 0.26 | 0.02 | 0.13 | 0.14 | 0.05 | 0.06 | -0.01 | 0.03 | 0.05 | -0.08 |
|  | Other diseases | 1.08 | 0.94 | 0.57 | 0.60 | 0.47 | 0.45 | 0.30 | 0.25 | 0.19 | 0.27 | 0.07 | 0.08 |
|  | Total | 5.71 | 2.98 | 2.78 | 1.83 | 1.90 | 2.19 | 1.76 | 1.40 | 1.16 | 1.42 | 0.90 | 1.25 |
| Kazakhstan | Infectious diseases |  |  |  |  |  |  |  | 0.18 | -0.16 | 0.19 | 0.14 | 0.11 |
|  | Malignant neoplasms |  |  |  |  |  |  |  | 0.00 | 0.11 | 0.05 | 0.16 | 0.14 |
|  | Cardiovascular diseases |  |  |  |  |  |  |  | 0.05 | -1.65 | 0.07 | -0.10 | 1.69 |
|  | Respiratory diseases |  |  |  |  |  |  |  | 0.64 | -0.27 | 0.56 | 0.34 | 0.13 |
|  | Digestive system diseases |  |  |  |  |  |  |  | 0.05 | -0.10 | -0.03 | -0.11 | 0.03 |
|  | Pregnancy |  |  |  |  |  |  |  | 0.00 | 0.01 | 0.02 | 0.01 | 0.00 |
|  | Perinatal diseases |  |  |  |  |  |  |  | -0.08 | 0.03 | 0.08 | -0.04 | -0.23 |
|  | External causes |  |  |  |  |  |  |  | -0.07 | -0.32 | 0.11 | -0.05 | 0.32 |
|  | Other diseases |  |  |  |  |  |  |  | -0.10 | -0.52 | 0.04 | 0.10 | -0.59 |
|  | Total |  |  |  |  |  |  |  | 0.67 | -2.88 | 1.10 | 0.44 | 1.62 |
| Kuwait | Infectious diseases |  |  |  |  |  | 0.51 | 0.53 |  |  | 0.19 |  |  |
|  | Malignant neoplasms |  |  |  |  |  | -0.16 | 0.12 |  |  | 0.22 |  |  |
|  | Cardiovascular diseases |  |  |  |  |  | -0.15 | 0.87 |  |  | 1.14 |  |  |
|  | Respiratory diseases |  |  |  |  |  | 0.31 | 0.41 |  |  | 0.24 |  |  |
|  | Digestive system diseases |  |  |  |  |  | 0.02 | 0.12 |  |  | -0.01 |  |  |
|  | Pregnancy |  |  |  |  |  | 0.01 | 0.01 |  |  | 0.00 |  |  |
|  | Perinatal diseases |  |  |  |  |  | 0.05 | 0.02 |  |  | -0.01 |  |  |
|  | External causes |  |  |  |  |  | 0.11 | 0.13 |  |  | 0.13 |  |  |
|  | Other diseases |  |  |  |  |  | 0.55 | 0.16 |  |  | 0.91 |  |  |
|  | Total |  |  |  |  |  | 1.25 | 2.37 |  |  | 2.83 |  |  |
| Kyrgyzstan | Infectious diseases |  |  |  |  |  |  |  | 0.50 | 0.05 | 0.04 | 0.20 |  |
|  | Malignant neoplasms |  |  |  |  |  |  |  | 0.04 | 0.09 | 0.04 | -0.01 |  |
|  | Cardiovascular diseases |  |  |  |  |  |  |  | 0.16 | -1.11 | 0.27 | -0.40 |  |
|  | Respiratory diseases |  |  |  |  |  |  |  | 1.12 | 0.11 | 0.69 | 0.39 |  |
|  | Digestive system diseases |  |  |  |  |  |  |  | -0.02 | -0.14 | -0.02 | -0.08 |  |
|  | Pregnancy |  |  |  |  |  |  |  | 0.01 | 0.04 | 0.00 | -0.02 |  |
|  | Perinatal diseases |  |  |  |  |  |  |  | -0.09 | 0.05 | -0.04 | -0.67 |  |
|  | External causes |  |  |  |  |  |  |  | -0.26 | -0.01 | 0.32 | -0.02 |  |
|  | Other diseases |  |  |  |  |  |  |  | -0.52 | -1.24 | 0.86 | 0.10 |  |
|  | Total |  |  |  |  |  |  |  | 0.94 | -2.15 | 2.17 | -0.50 |  |

| **Country** | **Cause of death** | **1950-55** | **1955-60** | **1960-65** | **1965-70** | **1970-75** | **1975-80** | **1980-85** | **1985-90** | **1990-95** | **1995-2000** | **2000-05** | **2005-10** |
| --- | --- | --- | --- | --- | --- | --- | --- | --- | --- | --- | --- | --- | --- |
| Latvia | Infectious diseases |  |  |  |  |  |  | 0.07 | 0.06 | -0.07 | 0.05 | 0.02 | 0.02 |
|  | Malignant neoplasms |  |  |  |  |  |  | -0.12 | 0.02 | 0.05 | 0.06 | 0.00 | -0.04 |
|  | Cardiovascular diseases |  |  |  |  |  |  | -0.07 | 0.70 | -0.42 | **1.19** | 0.24 | 1.28 |
|  | Respiratory diseases |  |  |  |  |  |  | 0.07 | 0.15 | -0.06 | 0.13 | 0.02 | 0.04 |
|  | Digestive system diseases |  |  |  |  |  |  | -0.02 | -0.02 | -0.10 | 0.00 | -0.06 | 0.03 |
|  | Pregnancy |  |  |  |  |  |  | 0.00 | -0.01 | 0.02 | 0.00 | 0.01 | -0.01 |
|  | Perinatal diseases |  |  |  |  |  |  | -0.04 | -0.07 | -0.25 | 0.27 | 0.06 | 0.04 |
|  | External causes |  |  |  |  |  |  | 0.24 | -0.14 | -0.39 | 0.37 | 0.23 | 0.51 |
|  | Other diseases |  |  |  |  |  |  | 0.09 | -0.34 | -0.27 | 0.36 | -0.10 | 0.12 |
|  | Total |  |  |  |  |  |  | 0.20 | 0.36 | -1.49 | 2.43 | 0.43 | 2.03 |
| Lithuania | Infectious diseases |  |  |  |  |  |  |  | 0.03 | -0.04 | 0.03 | 0.01 | 0.00 |
|  | Malignant neoplasms |  |  |  |  |  |  |  | -0.01 | -0.07 | 0.07 | 0.14 | 0.15 |
|  | Cardiovascular diseases |  |  |  |  |  |  |  | 0.35 | -0.14 | 0.97 | 0.02 | 0.52 |
|  | Respiratory diseases |  |  |  |  |  |  |  | 0.22 | -0.02 | 0.10 | 0.01 | 0.05 |
|  | Digestive system diseases |  |  |  |  |  |  |  | 0.00 | -0.06 | -0.06 | -0.17 | -0.02 |
|  | Pregnancy |  |  |  |  |  |  |  | 0.00 | 0.01 | 0.00 | 0.00 | 0.26 |
|  | Perinatal diseases |  |  |  |  |  |  |  | -0.07 | 0.00 | 0.16 | -0.01 | 0.06 |
|  | External causes |  |  |  |  |  |  |  | -0.08 | -0.38 | 0.28 | 0.07 | 0.38 |
|  | Other diseases |  |  |  |  |  |  |  | -0.10 | -0.20 | 0.52 | 0.02 | 0.14 |
|  | Total |  |  |  |  |  |  |  | 0.34 | -0.90 | 2.06 | 0.10 | 1.29 |
| Mauritius | Infectious diseases |  |  |  | -2.16 | 0.73 | 0.89 | 0.62 | 0.19 | 0.03 | 0.12 | -0.06 |  |
|  | Malignant neoplasms |  |  |  | 0.01 | 0.00 | -0.01 | -0.02 | 0.01 | -0.08 | 0.03 | -0.01 |  |
|  | Cardiovascular diseases |  |  |  | -0.28 | 0.06 | -0.51 | -0.38 | 0.95 | 0.19 | -0.12 | 1.41 |  |
|  | Respiratory diseases |  |  |  | -0.44 | 0.38 | 0.79 | 0.21 | 0.33 | 0.14 | 0.06 | 0.20 |  |
|  | Digestive system diseases |  |  |  | 1.72 | 0.05 | 0.05 | 0.06 | 0.05 | 0.03 | -0.03 | 0.00 |  |
|  | Pregnancy |  |  |  | 0.04 | 0.04 | 0.06 | 0.03 | 0.05 | 0.00 | 0.03 | 0.01 |  |
|  | Perinatal diseases |  |  |  | 0.51 | 0.15 | 0.13 | -0.09 | 0.16 | 0.06 | 0.16 | 0.13 |  |
|  | External causes |  |  |  | -0.03 | -0.08 | -0.05 | 0.00 | 0.03 | 0.19 | 0.00 | 0.09 |  |
|  | Other diseases |  |  |  | 1.85 | 0.12 | 1.92 | 1.11 | -0.10 | 0.23 | 0.56 | -1.17 |  |
|  | Total |  |  |  | 1.21 | 1.47 | 3.25 | 1.55 | 1.68 | 0.78 | 0.80 | 0.61 |  |
| Mexico | Infectious diseases |  |  | 0.71 | -1.98 | 1.33 |  |  | 0.35 | 0.55 |  |  |  |
|  | Malignant neoplasms |  |  | -0.05 | 0.07 | -0.02 |  |  | -0.01 | 0.02 |  |  |  |
|  | Cardiovascular diseases |  |  | 0.60 | -0.50 | -0.24 |  |  | 0.07 | -0.11 |  |  |  |
|  | Respiratory diseases |  |  | 0.29 | -0.06 | 1.04 |  |  | 0.19 | 0.12 |  |  |  |
|  | Digestive system diseases |  |  | 1.08 | 1.91 | 0.25 |  |  | 0.13 | -0.02 |  |  |  |
|  | Pregnancy |  |  | 0.06 | 0.05 | 0.12 |  |  | 0.03 | 0.01 |  |  |  |
|  | Perinatal diseases |  |  | 0.13 | 0.88 | 0.13 |  |  | -0.10 | 0.08 |  |  |  |
|  | External causes |  |  | -0.01 | 0.01 | -0.17 |  |  | 0.14 | 0.11 |  |  |  |
|  | Other diseases |  |  | -1.23 | 1.63 | 1.22 |  |  | 0.12 | 0.09 |  |  |  |
|  | Total |  |  | 1.59 | 2.01 | 3.66 |  |  | 0.93 | 0.85 |  |  |  |
| Netherlands | Infectious diseases | 0.39 | 0.14 | 0.06 | -0.04 | 0.03 | 0.04 | 0.00 | -0.02 | -0.03 |  | 0.01 | 0.01 |
|  | Malignant neoplasms | 0.10 | 0.10 | -0.01 | 0.05 | 0.17 | 0.08 | 0.05 | -0.02 | 0.05 |  | 0.03 | 0.07 |
|  | Cardiovascular diseases | -0.03 | **0.57** | 0.34 | 0.18 | 0.53 | 0.63 | 0.39 | 0.55 | 0.20 |  | 0.57 | 0.58 |
|  | Respiratory diseases | 0.25 | 0.09 | 0.19 | -0.13 | 0.14 | 0.11 | -0.08 | -0.01 | -0.08 |  | 0.05 | 0.08 |
|  | Digestive system diseases | 0.06 | 0.04 | 0.03 | 0.06 | 0.05 | 0.00 | 0.03 | 0.01 | 0.00 |  | 0.02 | 0.08 |
|  | Pregnancy | 0.04 | 0.03 | 0.02 | 0.02 | 0.01 | 0.00 | 0.00 | 0.00 | 0.00 |  | 0.00 | 0.00 |
|  | Perinatal diseases | 0.17 | 0.09 | 0.00 | 0.13 | 0.07 | 0.11 | 0.04 | 0.01 | 0.02 |  | 0.03 | 0.02 |
|  | External causes | -0.05 | -0.02 | -0.11 | -0.13 | 0.12 | 0.15 | 0.12 | 0.07 | 0.08 |  | 0.03 | 0.02 |
|  | Other diseases | 0.40 | 0.38 | 0.16 | 0.19 | 0.12 | 0.32 | -0.06 | -0.19 | 0.01 |  | 0.26 | 0.24 |
|  | Total | 1.34 | 1.42 | 0.69 | 0.33 | 1.25 | 1.44 | 0.48 | 0.39 | 0.26 |  | 1.00 | 1.12 |
| New Zealand | Infectious diseases | 0.54 | 0.19 | 0.08 | -0.04 | 0.04 | 0.09 | 0.00 | 0.00 | 0.00 | 0.02 | 0.02 |  |
|  | Malignant neoplasms | 0.08 | 0.03 | -0.02 | -0.03 | -0.15 | 0.01 | 0.02 | -0.12 | 0.15 | 0.31 | 0.20 |  |
|  | Cardiovascular diseases | **0.78** | 0.25 | 0.12 | 0.32 | 0.42 | 0.61 | 0.78 | 0.83 | 0.66 | 0.64 | 0.62 |  |
|  | Respiratory diseases | -0.01 | -0.08 | -0.04 | -0.03 | 0.13 | 0.10 | 0.03 | 0.20 | 0.06 | 0.29 | 0.09 |  |
|  | Digestive system diseases | 0.04 | 0.08 | 0.12 | 0.11 | -0.04 | 0.06 | -0.02 | 0.01 | 0.06 | 0.07 | 0.01 |  |
|  | Pregnancy | 0.03 | 0.02 | 0.03 | 0.01 | 0.02 | 0.01 | 0.00 | 0.00 | 0.01 | 0.00 | 0.00 |  |
|  | Perinatal diseases | 0.18 | 0.00 | 0.16 | 0.09 | 0.17 | 0.09 | 0.07 | 0.01 | 0.01 | -0.02 | 0.01 |  |
|  | External causes | -0.13 | 0.04 | -0.09 | -0.07 | -0.04 | 0.08 | 0.15 | 0.09 | 0.09 | 0.08 | 0.05 |  |
|  | Other diseases | 0.28 | 0.09 | 0.20 | 0.07 | 0.09 | -0.01 | 0.05 | 0.13 | -0.02 | 0.05 | -0.02 |  |
|  | Total | 1.78 | 0.62 | 0.56 | 0.44 | 0.65 | 1.03 | 1.09 | 1.17 | 1.03 | 1.43 | 0.98 |  |
| Norway | Infectious diseases |  | 0.14 | 0.06 | -0.02 | 0.01 | 0.01 | 0.03 | 0.00 | -0.01 | -0.01 | -0.01 | -0.03 |
|  | Malignant neoplasms |  | 0.16 | 0.10 | -0.08 | 0.09 | 0.02 | -0.04 | 0.04 | -0.11 | 0.19 | 0.15 | 0.16 |
|  | Cardiovascular diseases |  | -0.01 | **0.16** | 0.35 | 0.47 | 0.58 | 0.45 | 0.27 | 0.59 | 0.46 | 0.82 | 0.60 |
|  | Respiratory diseases |  | -0.02 | 0.12 | -0.12 | 0.08 | 0.19 | 0.06 | 0.00 | 0.06 | 0.06 | 0.15 | 0.00 |
|  | Digestive system diseases |  | 0.04 | 0.02 | 0.11 | 0.01 | 0.00 | 0.00 | 0.02 | 0.03 | -0.01 | 0.04 | 0.02 |
|  | Pregnancy |  | 0.03 | 0.01 | 0.01 | 0.01 | 0.00 | 0.01 | 0.00 | 0.00 | 0.00 | 0.55 | 0.00 |
|  | Perinatal diseases |  | 0.05 | 0.03 | 0.12 | 0.17 | 0.13 | 0.00 | -0.04 | 0.09 | 0.00 | 0.00 | 0.04 |
|  | External causes |  | 0.00 | -0.06 | -0.01 | -0.03 | 0.04 | 0.00 | 0.00 | 0.17 | -0.02 | -0.04 | 0.05 |
|  | Other diseases |  | 0.17 | 0.11 | 0.32 | 0.17 | 0.10 | 0.00 | -0.04 | 0.12 | -0.10 | 0.08 | -0.11 |
|  | Total |  | 0.58 | 0.56 | 0.69 | 0.97 | 1.06 | 0.51 | 0.23 | 0.94 | 0.57 | 1.18 | 0.73 |

| **Country** | **Cause of death** | **1950-55** | **1955-60** | **1960-65** | **1965-70** | **1970-75** | **1975-80** | **1980-85** | **1985-90** | **1990-95** | **1995-2000** | **2000-05** | **2005-10** |
| --- | --- | --- | --- | --- | --- | --- | --- | --- | --- | --- | --- | --- | --- |
| Poland | Infectious diseases |  |  | 0.34 | 0.17 | 0.21 | 0.03 | 0.07 | 0.03 | 0.03 |  | 0.01 | -0.01 |
|  | Malignant neoplasms |  |  | -0.36 | 0.03 | 0.05 | -0.05 | -0.06 | -0.02 | 0.02 |  | 0.06 | 0.18 |
|  | Cardiovascular diseases |  |  | -0.25 | -0.18 | 0.06 | -0.25 | -0.41 | 0.11 | **0.59** |  | 0.85 | 0.63 |
|  | Respiratory diseases |  |  | 0.44 | -0.10 | 0.48 | 0.22 | 0.03 | 0.13 | 0.08 |  | 0.05 | -0.01 |
|  | Digestive system diseases |  |  | 0.17 | 0.21 | 0.05 | 0.04 | 0.03 | 0.03 | 0.02 |  | 0.00 | 0.03 |
|  | Pregnancy |  |  | 0.02 | 0.01 | 0.01 | 0.00 | 0.00 | 0.00 | 0.00 |  | 0.00 | 0.00 |
|  | Perinatal diseases |  |  | 0.21 | 0.31 | 0.13 | 0.07 | 0.01 | 0.07 | 0.00 |  | 0.04 | 0.04 |
|  | External causes |  |  | -0.08 | -0.07 | -0.01 | -0.01 | 0.02 | -0.02 | 0.07 |  | 0.09 | 0.10 |
|  | Other diseases |  |  | 1.63 | 0.51 | 0.57 | 0.14 | 0.16 | 0.19 | 0.06 |  | 0.32 | 0.08 |
|  | Total |  |  | 2.12 | 0.91 | 1.57 | 0.19 | -0.14 | 0.53 | 0.88 |  | 1.42 | 1.03 |
| Portugal | Infectious diseases |  | 0.43 | 0.46 | 0.02 | 0.20 | 0.19 | 0.24 | 0.02 | -0.06 | -0.05 |  |  |
|  | Malignant neoplasms |  | -0.04 | -0.09 | -0.07 | 0.07 | 0.02 | 0.01 | 0.00 | 0.10 | 0.10 |  |  |
|  | Cardiovascular diseases |  | 0.49 | 0.01 | -0.18 | 0.02 | 0.54 | **0.71** | 0.48 | 0.69 | 0.74 |  |  |
|  | Respiratory diseases |  | -0.01 | 0.16 | 0.02 | 0.73 | 0.60 | 0.31 | 0.14 | 0.05 | -0.08 |  |  |
|  | Digestive system diseases |  | 0.38 | 0.90 | 0.96 | 0.45 | 0.32 | 0.03 | 0.07 | 0.05 | 0.07 |  |  |
|  | Pregnancy |  | 0.02 | 0.05 | 0.02 | 0.03 | 0.03 | 0.01 | 0.00 | 0.00 | 0.00 |  |  |
|  | Perinatal diseases |  | 0.10 | 0.61 | 0.23 | 0.48 | 0.13 | 0.09 | 0.20 | 0.15 | 0.06 |  |  |
|  | External causes |  | 0.09 | 0.01 | -0.01 | -0.09 | -0.08 | 0.06 | 0.07 | 0.16 | 0.15 |  |  |
|  | Other diseases |  | 0.94 | 0.25 | 0.27 | 0.29 | 0.52 | 0.49 | 0.17 | 0.23 | 0.12 |  |  |
|  | Total |  | 2.42 | 2.35 | 1.27 | 2.18 | 2.26 | 1.95 | 1.16 | 1.36 | 1.13 |  |  |
| Puerto Rico | Infectious diseases |  |  |  |  | 0.24 |  | 0.00 | -0.07 |  |  |  |  |
|  | Malignant neoplasms |  |  |  |  | 0.23 |  | 0.00 | 0.05 |  |  |  |  |
|  | Cardiovascular diseases |  |  |  |  | -0.02 |  | **0.71** | 0.49 |  |  |  |  |
|  | Respiratory diseases |  |  |  |  | 0.19 |  | -0.11 | 0.07 |  |  |  |  |
|  | Digestive system diseases |  |  |  |  | 0.09 |  | 0.08 | 0.05 |  |  |  |  |
|  | Pregnancy |  |  |  |  | 0.02 |  | 0.00 | -0.01 |  |  |  |  |
|  | Perinatal diseases |  |  |  |  | 0.17 |  | 0.14 | 0.10 |  |  |  |  |
|  | External causes |  |  |  |  | 0.15 |  | -0.10 | 0.04 |  |  |  |  |
|  | Other diseases |  |  |  |  | 0.67 |  | -0.01 | -0.74 |  |  |  |  |
|  | Total |  |  |  |  | 1.74 |  | 0.71 | -0.01 |  |  |  |  |
| Republic of Korea | Infectious diseases |  |  |  |  |  |  |  | 0.17 | 0.04 | 0.00 | 0.04 | -0.02 |
|  | Malignant neoplasms |  |  |  |  |  |  |  | -0.07 | -0.22 | 0.11 | 0.10 | 0.21 |
|  | Cardiovascular diseases |  |  |  |  |  |  |  | **0.68** | 0.02 | 0.96 | 0.63 | 0.65 |
|  | Respiratory diseases |  |  |  |  |  |  |  | 0.16 | -0.05 | -0.02 | 0.18 | -0.03 |
|  | Digestive system diseases |  |  |  |  |  |  |  | 0.21 | 0.01 | 0.15 | 0.15 | 0.02 |
|  | Pregnancy |  |  |  |  |  |  |  | 0.00 | 0.00 | 0.00 | 0.00 | 0.00 |
|  | Perinatal diseases |  |  |  |  |  |  |  | -0.01 | 0.00 | -0.15 | 0.01 | 0.05 |
|  | External causes |  |  |  |  |  |  |  | -0.15 | -0.07 | 0.23 | 0.06 | 0.04 |
|  | Other diseases |  |  |  |  |  |  |  | 0.94 | -0.36 | 0.19 | 0.86 | 1.15 |
|  | Total |  |  |  |  |  |  |  | 1.94 | -0.64 | 1.47 | 2.03 | 2.07 |
| Republic of Moldova | Infectious diseases |  |  |  |  |  |  |  | 0.21 | -0.02 | 0.03 | 0.01 | 0.03 |
|  | Malignant neoplasms |  |  |  |  |  |  |  | -0.21 | 0.01 | 0.09 | -0.03 | 0.02 |
|  | Cardiovascular diseases |  |  |  |  |  |  |  | 1.35 | -0.83 | -0.10 | 0.16 | 1.02 |
|  | Respiratory diseases |  |  |  |  |  |  |  | 0.49 | -0.09 | 0.16 | 0.15 | 0.20 |
|  | Digestive system diseases |  |  |  |  |  |  |  | 0.32 | -0.13 | 0.24 | -0.17 | 0.27 |
|  | Pregnancy |  |  |  |  |  |  |  | 0.01 | 0.02 | 0.01 | 0.01 | 0.00 |
|  | Perinatal diseases |  |  |  |  |  |  |  | 0.04 | -0.05 | 0.10 | 0.09 | -0.03 |
|  | External causes |  |  |  |  |  |  |  | 0.14 | 0.05 | 0.25 | 0.04 | 0.20 |
|  | Other diseases |  |  |  |  |  |  |  | -0.86 | -0.77 | 0.89 | 0.36 | 0.13 |
|  | Total |  |  |  |  |  |  |  | 1.49 | -1.82 | 1.66 | 0.63 | 1.83 |
| Romania | Infectious diseases |  |  |  |  | 0.44 |  | 0.06 | -0.05 | 0.01 | 0.02 | 0.08 | 0.05 |
|  | Malignant neoplasms |  |  |  |  | 0.05 |  | 0.01 | -0.02 | -0.10 | -0.04 | 0.00 | 0.10 |
|  | Cardiovascular diseases |  |  |  |  | 0.39 |  | 0.08 | **0.33** | -0.11 | 0.74 | 0.67 | 0.99 |
|  | Respiratory diseases |  |  |  |  | 0.68 |  | 0.33 | 0.23 | 0.25 | 0.31 | 0.24 | 0.13 |
|  | Digestive system diseases |  |  |  |  | 0.04 |  | -0.05 | 0.00 | -0.08 | 0.09 | 0.08 | -0.05 |
|  | Pregnancy |  |  |  |  | -0.01 |  | 0.00 | 0.05 | 0.06 | 0.01 | 0.01 | 0.00 |
|  | Perinatal diseases |  |  |  |  | 0.20 |  | 0.10 | -0.03 | -0.07 | -0.02 | 0.01 | 0.15 |
|  | External causes |  |  |  |  | 0.03 |  | 0.03 | -0.09 | 0.06 | 0.20 | 0.10 | 0.12 |
|  | Other diseases |  |  |  |  | 0.04 |  | -0.08 | 0.01 | 0.13 | 0.19 | 0.06 | 0.05 |
|  | Total |  |  |  |  | 1.87 |  | 0.49 | 0.43 | 0.15 | 1.51 | 1.24 | 1.53 |
| Russian Federation | Infectious diseases |  |  |  |  |  |  | 0.07 | 0.11 | -0.03 | 0.00 | -0.02 | 0.00 |
|  | Malignant neoplasms |  |  |  |  |  |  | -0.02 | -0.05 | -0.01 | 0.06 | 0.09 | 0.04 |
|  | Cardiovascular diseases |  |  |  |  |  |  | -0.09 | 0.64 | -1.10 | 0.10 | 0.00 | 1.27 |
|  | Respiratory diseases |  |  |  |  |  |  | 0.28 | 0.34 | -0.01 | 0.10 | 0.09 | 0.07 |
|  | Digestive system diseases |  |  |  |  |  |  | 0.01 | 0.03 | -0.15 | 0.01 | -0.22 | 0.04 |
|  | Pregnancy |  |  |  |  |  |  | 0.01 | 0.01 | 0.01 | 0.01 | 0.01 | 0.00 |
|  | Perinatal diseases |  |  |  |  |  |  | -0.12 | -0.03 | -0.01 | 0.07 | 0.11 | 0.07 |
|  | External causes |  |  |  |  |  |  | 0.34 | -0.14 | -0.79 | 0.14 | 0.10 | 0.51 |
|  | Other diseases |  |  |  |  |  |  | -0.19 | 0.00 | -0.48 | 0.11 | 0.15 | 0.20 |
|  | Total |  |  |  |  |  |  | 0.31 | 0.91 | -2.58 | 0.59 | 0.29 | 2.19 |

| **Country** | **Cause of death** | **1950-55** | **1955-60** | **1960-65** | **1965-70** | **1970-75** | **1975-80** | **1980-85** | **1985-90** | **1990-95** | **1995-2000** | **2000-05** | **2005-10** |
| --- | --- | --- | --- | --- | --- | --- | --- | --- | --- | --- | --- | --- | --- |
| Singapore | Infectious diseases |  |  |  | 0.09 | 0.17 | 0.14 | 0.07 | 0.10 | 0.07 | 0.09 | 0.02 | 0.07 |
|  | Malignant neoplasms |  |  |  | 0.12 | -0.08 | -0.21 | 0.09 | 0.17 | 0.12 | 0.17 | 0.41 | 0.09 |
|  | Cardiovascular diseases |  |  |  | -0.24 | 0.03 | -0.21 | **0.52** | 0.63 | 0.61 | 0.65 | 1.35 | 0.65 |
|  | Respiratory diseases |  |  |  | -0.16 | 0.02 | -0.03 | 0.43 | 0.41 | 0.06 | 0.46 | 0.10 | 0.14 |
|  | Digestive system diseases |  |  |  | 0.20 | 0.07 | 0.08 | 0.05 | 0.07 | 0.04 | 0.05 | 0.02 | 0.03 |
|  | Pregnancy |  |  |  | 0.04 | 0.02 | 0.02 | 0.00 | 0.00 | 0.00 | 0.00 | 0.00 | 0.00 |
|  | Perinatal diseases |  |  |  | 0.33 | 0.26 | -0.02 | 0.15 | 0.09 | 0.07 | 0.02 | 0.01 | 0.00 |
|  | External causes |  |  |  | -0.10 | 0.05 | 0.04 | 0.02 | 0.07 | 0.17 | 0.03 | 0.05 | 0.08 |
|  | Other diseases |  |  |  | 1.47 | 0.68 | 0.88 | 0.39 | 0.53 | 0.58 | 0.09 | 0.08 | 0.21 |
|  | Total |  |  |  | 1.76 | 1.22 | 0.68 | 1.71 | 2.08 | 1.72 | 1.55 | 2.06 | 1.27 |
| Slovenia | Infectious diseases |  |  |  |  |  |  |  | -0.01 | 0.03 | 0.02 | -0.03 | 0.05 |
|  | Malignant neoplasms |  |  |  |  |  |  |  | 0.03 | -0.07 | 0.15 | 0.13 | 0.03 |
|  | Cardiovascular diseases |  |  |  |  |  |  |  | **0.87** | 0.72 | 0.92 | 0.56 | 0.74 |
|  | Respiratory diseases |  |  |  |  |  |  |  | -0.06 | 0.01 | -0.02 | 0.13 | 0.15 |
|  | Digestive system diseases |  |  |  |  |  |  |  | 0.14 | 0.03 | 0.05 | 0.15 | 0.07 |
|  | Pregnancy |  |  |  |  |  |  |  | 0.01 | 0.00 | 0.95 | 1.24 | 0.68 |
|  | Perinatal diseases |  |  |  |  |  |  |  | 0.18 | 0.07 | 0.00 | -0.01 | 0.03 |
|  | External causes |  |  |  |  |  |  |  | 0.09 | 0.01 | 0.28 | 0.08 | 0.11 |
|  | Other diseases |  |  |  |  |  |  |  | 0.04 | 0.02 | 0.05 | 0.22 | 0.41 |
|  | Total |  |  |  |  |  |  |  | 1.27 | 0.82 | 1.45 | 1.21 | 1.60 |
| Spain | Infectious diseases |  | 0.37 | 0.29 |  |  | 0.09 | 0.08 | -0.05 | -0.11 | 0.13 | 0.01 | 0.04 |
|  | Malignant neoplasms |  | -0.15 | -0.05 |  |  | 0.15 | 0.07 | -0.02 | 0.04 | 0.15 | 0.15 | 0.07 |
|  | Cardiovascular diseases |  | 0.37 | 0.32 |  |  | **0.77** | 0.74 | 0.73 | 0.80 | 0.73 | 0.55 | 0.52 |
|  | Respiratory diseases |  | 0.34 | 0.39 |  |  | 0.47 | 0.18 | 0.07 | 0.11 | -0.09 | 0.09 | 0.10 |
|  | Digestive system diseases |  | 0.38 | 0.19 |  |  | 0.09 | 0.05 | 0.03 | 0.07 | 0.05 | 0.04 | 0.06 |
|  | Pregnancy |  | 0.01 | 0.03 |  |  | 0.02 | 0.01 | 0.00 | 0.00 | 0.00 | 0.00 | 0.00 |
|  | Perinatal diseases |  | 0.27 | 0.22 |  |  | 0.04 | 0.11 | 0.04 | 0.06 | 0.01 | 0.01 | 0.01 |
|  | External causes |  | 0.00 | 0.01 |  |  | 0.01 | 0.04 | -0.05 | 0.12 | 0.04 | 0.04 | 0.11 |
|  | Other diseases |  | 0.93 | 0.49 |  |  | 0.41 | 0.01 | 0.04 | 0.08 | -0.01 | 0.09 | 0.07 |
|  | Total |  | 2.51 | 1.89 |  |  | 2.07 | 1.29 | 0.81 | 1.17 | 1.01 | 0.98 | 0.98 |
| Sweden | Infectious diseases |  | 0.08 | 0.05 | -0.01 | 0.01 | 0.01 | 0.00 | 0.02 | -0.01 |  | -0.01 | -0.06 |
|  | Malignant neoplasms |  | -0.01 | 0.12 | -0.03 | -0.04 | 0.15 | 0.20 | 0.05 | 0.09 |  | 0.05 | 0.21 |
|  | Cardiovascular diseases |  | **0.50** | 0.55 | 0.49 | 0.49 | 0.39 | 0.53 | 0.67 | 0.59 |  | 0.69 | 0.41 |
|  | Respiratory diseases |  | 0.09 | 0.07 | 0.06 | 0.19 | -0.06 | -0.06 | 0.09 | 0.02 |  | 0.08 | 0.05 |
|  | Digestive system diseases |  | 0.08 | 0.00 | 0.10 | 0.03 | 0.10 | 0.10 | -0.04 | 0.03 |  | 0.01 | 0.02 |
|  | Pregnancy |  | 0.02 | 0.01 | 0.01 | 0.01 | 0.00 | 0.00 | 0.00 | 0.00 |  | 0.00 | 0.00 |
|  | Perinatal diseases |  | 0.04 | 0.12 | 0.15 | 0.13 | 0.11 | 0.02 | 0.02 | 0.02 |  | 0.01 | 0.02 |
|  | External causes |  | -0.02 | -0.09 | -0.09 | 0.00 | 0.12 | 0.16 | 0.03 | 0.13 |  | -0.07 | 0.11 |
|  | Other diseases |  | 0.26 | 0.21 | 0.25 | 0.03 | 0.15 | 0.06 | -0.22 | 0.12 |  | 0.00 | -0.07 |
|  | Total |  | 1.04 | 1.04 | 0.93 | 0.85 | 0.98 | 1.01 | 0.61 | 0.98 |  | 0.77 | 0.70 |
| Switzerland | Infectious diseases |  | 0.21 | 0.09 | 0.02 | 0.05 | 0.04 | 0.02 | -0.07 | -0.04 | 0.09 | 0.04 | 0.00 |
|  | Malignant neoplasms |  | 0.12 | 0.10 | 0.03 | 0.18 | 0.03 | 0.03 | 0.15 | 0.27 | 0.23 | 0.16 | 0.10 |
|  | Cardiovascular diseases |  | **0.79** | 0.33 | 0.64 | 0.69 | 0.47 | 0.89 | 0.55 | 0.60 | 0.51 | 0.75 | 0.33 |
|  | Respiratory diseases |  | 0.18 | 0.15 | -0.13 | 0.18 | 0.14 | 0.05 | -0.07 | 0.12 | -0.04 | 0.12 | 0.03 |
|  | Digestive system diseases |  | 0.05 | 0.04 | 0.09 | 0.09 | 0.08 | 0.05 | 0.05 | -0.02 | -0.01 | 0.02 | 0.01 |
|  | Pregnancy |  | 0.03 | 0.01 | 0.02 | 0.02 | 0.00 | 0.00 | 0.00 | 0.00 | 0.00 | 0.00 | 0.00 |
|  | Perinatal diseases |  | 0.30 | 0.14 | 0.17 | 0.18 | 0.12 | 0.06 | 0.01 | 0.01 | -0.01 | -0.01 | -0.02 |
|  | External causes |  | -0.02 | 0.02 | -0.05 | 0.09 | 0.01 | 0.07 | 0.08 | 0.26 | 0.14 | 0.04 | 0.10 |
|  | Other diseases |  | 0.21 | 0.03 | 0.07 | 0.45 | 0.16 | 0.10 | 0.03 | -0.36 | -0.05 | 0.06 | 0.05 |
|  | Total |  | 1.86 | 0.91 | 0.87 | 1.93 | 1.05 | 1.27 | 0.73 | 0.84 | 0.86 | 1.18 | 0.59 |
| TFYR Macedonia | Infectious diseases |  |  |  |  |  |  |  |  |  | 0.15 | 0.06 | 0.06 |
|  | Malignant neoplasms |  |  |  |  |  |  |  |  |  | -0.08 | 0.08 | -0.06 |
|  | Cardiovascular diseases |  |  |  |  |  |  |  |  |  | 0.09 | **0.13** | 0.59 |
|  | Respiratory diseases |  |  |  |  |  |  |  |  |  | 0.20 | 0.00 | 0.02 |
|  | Digestive system diseases |  |  |  |  |  |  |  |  |  | -0.01 | 0.01 | 0.01 |
|  | Pregnancy |  |  |  |  |  |  |  |  |  | 0.00 | 0.00 | 0.03 |
|  | Perinatal diseases |  |  |  |  |  |  |  |  |  | 0.21 | -0.05 | 0.05 |
|  | External causes |  |  |  |  |  |  |  |  |  | 0.02 | 0.05 | 0.03 |
|  | Other diseases |  |  |  |  |  |  |  |  |  | 0.29 | 0.18 | 0.27 |
|  | Total |  |  |  |  |  |  |  |  |  | 0.88 | 0.46 | 0.96 |
| Tajikistan | Infectious diseases |  |  |  |  |  |  |  | -0.18 | 0.28 | 1.08 |  |  |
|  | Malignant neoplasms |  |  |  |  |  |  |  | 0.01 | 0.45 | -0.03 |  |  |
|  | Cardiovascular diseases |  |  |  |  |  |  |  | -0.50 | -1.39 | 0.57 |  |  |
|  | Respiratory diseases |  |  |  |  |  |  |  | 0.71 | 0.02 | 1.70 |  |  |
|  | Digestive system diseases |  |  |  |  |  |  |  | 0.47 | -0.07 | 0.04 |  |  |
|  | Pregnancy |  |  |  |  |  |  |  | 0.05 | 0.01 | 0.06 |  |  |
|  | Perinatal diseases |  |  |  |  |  |  |  | -0.06 | 0.05 | 0.18 |  |  |
|  | External causes |  |  |  |  |  |  |  | -0.05 | 0.17 | 0.17 |  |  |
|  | Other diseases |  |  |  |  |  |  |  | -0.31 | -0.54 | 0.37 |  |  |
|  | Total |  |  |  |  |  |  |  | 0.13 | -1.03 | 4.14 |  |  |

| **Country** | **Cause of death** | **1950-55** | **1955-60** | **1960-65** | **1965-70** | **1970-75** | **1975-80** | **1980-85** | **1985-90** | **1990-95** | **1995-2000** | **2000-05** | **2005-10** |
| --- | --- | --- | --- | --- | --- | --- | --- | --- | --- | --- | --- | --- | --- |
| Trinidad and Tobago | Infectious diseases |  |  |  |  | 0.18 | 0.82 | 0.40 | 0.01 |  |  |  |  |
|  | Malignant neoplasms |  |  |  |  | 0.05 | 0.06 | -0.11 | 0.12 |  |  |  |  |
|  | Cardiovascular diseases |  |  |  |  | **0.73** | -0.01 | 1.16 | 0.28 |  |  |  |  |
|  | Respiratory diseases |  |  |  |  | 0.40 | 0.35 | 0.25 | 0.05 |  |  |  |  |
|  | Digestive system diseases |  |  |  |  | -0.01 | -0.01 | 0.01 | 0.13 |  |  |  |  |
|  | Pregnancy |  |  |  |  | 0.04 | 0.08 | 0.04 | 0.00 |  |  |  |  |
|  | Perinatal diseases |  |  |  |  | 0.33 | 0.12 | -0.02 | 0.11 |  |  |  |  |
|  | External causes |  |  |  |  | 0.02 | 0.00 | 0.02 | 0.06 |  |  |  |  |
|  | Other diseases |  |  |  |  | -0.01 | 0.78 | -0.45 | -0.05 |  |  |  |  |
|  | Total |  |  |  |  | 1.73 | 2.19 | 1.33 | 0.71 |  |  |  |  |
| Turkmenistan | Infectious diseases |  |  |  |  |  |  |  | 0.24 | 0.01 |  |  |  |
|  | Malignant neoplasms |  |  |  |  |  |  |  | 0.22 | 0.14 |  |  |  |
|  | Cardiovascular diseases |  |  |  |  |  |  |  | -0.29 | -1.67 |  |  |  |
|  | Respiratory diseases |  |  |  |  |  |  |  | 1.79 | -0.15 |  |  |  |
|  | Digestive system diseases |  |  |  |  |  |  |  | -0.16 | 0.04 |  |  |  |
|  | Pregnancy |  |  |  |  |  |  |  | 0.03 | 0.02 |  |  |  |
|  | Perinatal diseases |  |  |  |  |  |  |  | -0.09 | 0.14 |  |  |  |
|  | External causes |  |  |  |  |  |  |  | -0.01 | 0.12 |  |  |  |
|  | Other diseases |  |  |  |  |  |  |  | -0.12 | -0.13 |  |  |  |
|  | Total |  |  |  |  |  |  |  | 1.61 | -1.48 |  |  |  |
| Ukraine | Infectious diseases |  |  |  |  |  |  |  | 0.05 | -0.06 | 0.01 | -0.13 |  |
|  | Malignant neoplasms |  |  |  |  |  |  |  | -0.11 | -0.01 | 0.09 | 0.09 |  |
|  | Cardiovascular diseases |  |  |  |  |  |  |  | 0.92 | -1.30 | 0.08 | -0.05 |  |
|  | Respiratory diseases |  |  |  |  |  |  |  | 0.22 | -0.01 | 0.14 | 0.12 |  |
|  | Digestive system diseases |  |  |  |  |  |  |  | 0.00 | -0.09 | 0.00 | -0.23 |  |
|  | Pregnancy |  |  |  |  |  |  |  | 0.01 | 0.01 | 0.01 | 0.00 |  |
|  | Perinatal diseases |  |  |  |  |  |  |  | 0.01 | 0.00 | 0.02 | 0.03 |  |
|  | External causes |  |  |  |  |  |  |  | -0.15 | -0.29 | 0.11 | 0.05 |  |
|  | Other diseases |  |  |  |  |  |  |  | -0.69 | -0.26 | 0.44 | 0.04 |  |
|  | Total |  |  |  |  |  |  |  | 0.25 | -2.02 | 0.89 | -0.08 |  |
| United Kingdom | Infectious diseases | 0.60 | 0.17 | 0.05 | -0.02 | 0.02 | 0.05 | 0.01 | 0.00 | -0.02 |  |  | 0.03 |
|  | Malignant neoplasms | 0.05 | 0.02 | -0.01 | -0.07 | -0.03 | -0.02 | -0.07 | 0.06 | 0.23 |  |  | 0.17 |
|  | Cardiovascular diseases | 0.49 | **0.37** | 0.44 | 0.31 | 0.31 | 0.56 | 0.63 | 0.66 | 0.63 |  |  | 0.70 |
|  | Respiratory diseases | 0.50 | -0.05 | 0.07 | -0.19 | 0.10 | 0.14 | 0.46 | 0.01 | -0.26 |  |  | 0.14 |
|  | Digestive system diseases | 0.10 | 0.05 | 0.02 | 0.08 | -0.01 | -0.01 | 0.01 | 0.01 | -0.01 |  |  | 0.04 |
|  | Pregnancy | 0.02 | 0.02 | 0.01 | 0.01 | 0.01 | 0.00 | 0.00 | 0.00 | 0.00 |  |  | 0.00 |
|  | Perinatal diseases | 0.13 | 0.10 | 0.13 | 0.13 | 0.09 | 0.13 | 0.07 | 0.01 | 0.01 |  |  | 0.02 |
|  | External causes | -0.02 | -0.02 | -0.05 | 0.03 | 0.04 | 0.07 | 0.10 | 0.08 | 0.07 |  |  | 0.03 |
|  | Other diseases | 0.26 | 0.18 | 0.13 | 0.16 | -0.03 | 0.14 | -0.16 | 0.11 | 0.27 |  |  | 0.00 |
|  | Total | 2.13 | 0.84 | 0.79 | 0.45 | 0.50 | 1.07 | 1.05 | 0.93 | 0.93 |  |  | 1.12 |
| United States of America | Infectious diseases | 0.29 | 0.08 | 0.04 | -0.01 | 0.01 | 0.03 | -0.04 | -0.08 | -0.08 | 0.03 | -0.01 |  |
|  | Malignant neoplasms | 0.09 | 0.10 | 0.03 | 0.02 | 0.04 | 0.00 | -0.08 | -0.03 | 0.05 | 0.17 | 0.21 |  |
|  | Cardiovascular diseases | **0.61** | 0.25 | 0.30 | 0.55 | 1.05 | 0.91 | 0.54 | 0.69 | 0.31 | 0.42 | 0.68 |  |
|  | Respiratory diseases | 0.10 | -0.06 | 0.00 | 0.01 | 0.14 | 0.05 | -0.14 | -0.12 | -0.05 | -0.01 | 0.06 |  |
|  | Digestive system diseases | 0.06 | 0.01 | -0.01 | 0.05 | 0.08 | 0.04 | 0.05 | 0.03 | 0.03 | -0.01 | 0.02 |  |
|  | Pregnancy | 0.04 | 0.01 | 0.02 | 0.02 | 0.01 | 0.00 | 0.00 | 0.00 | 0.00 | 0.00 | -0.01 |  |
|  | Perinatal diseases | 0.07 | 0.00 | 0.07 | 0.20 | 0.18 | 0.14 | 0.09 | 0.05 | 0.06 | 0.00 | 0.00 |  |
|  | External causes | 0.09 | 0.03 | -0.12 | -0.07 | 0.14 | 0.10 | 0.12 | 0.02 | 0.05 | 0.03 | -0.06 |  |
|  | Other diseases | 0.31 | 0.06 | -0.01 | 0.12 | 0.13 | 0.08 | -0.02 | -0.04 | -0.09 | -0.23 | -0.07 |  |
|  | Total | 1.64 | 0.48 | 0.32 | 0.90 | 1.79 | 1.34 | 0.52 | 0.52 | 0.27 | 0.41 | 0.82 |  |
| Uruguay | Infectious diseases |  | 0.10 |  | -0.32 | 0.02 |  | 0.12 | 0.19 |  |  |  |  |
|  | Malignant neoplasms |  | -0.10 |  | -0.06 | 0.19 |  | 0.12 | 0.00 |  |  |  |  |
|  | Cardiovascular diseases |  | -0.36 |  | -0.30 | 0.40 |  | 0.26 | 0.42 |  |  |  |  |
|  | Respiratory diseases |  | -0.06 |  | -0.22 | 0.19 |  | 0.09 | -0.04 |  |  |  |  |
|  | Digestive system diseases |  | 0.00 |  | 0.32 | 0.04 |  | 0.04 | 0.02 |  |  |  |  |
|  | Pregnancy |  | 0.04 |  | 0.02 | 0.01 |  | 0.03 | 0.02 |  |  |  |  |
|  | Perinatal diseases |  | -0.45 |  | 0.65 | -0.13 |  | 0.28 | 0.25 |  |  |  |  |
|  | External causes |  | 0.09 |  | -0.04 | 0.01 |  | 0.10 | -0.14 |  |  |  |  |
|  | Other diseases |  | -0.06 |  | -0.27 | 0.12 |  | 0.16 | 0.31 |  |  |  |  |
|  | Total |  | -0.80 |  | -0.22 | 0.85 |  | 1.20 | 1.03 |  |  |  |  |
| Uzbekistan | Infectious diseases |  |  |  |  |  |  |  | 0.46 | 0.22 | 0.29 | 0.14 |  |
|  | Malignant neoplasms |  |  |  |  |  |  |  | 0.07 | 0.20 | 0.11 | 0.04 |  |
|  | Cardiovascular diseases |  |  |  |  |  |  |  | -0.22 | -1.71 | 0.32 | 0.21 |  |
|  | Respiratory diseases |  |  |  |  |  |  |  | 0.61 | 0.22 | 0.63 | 0.52 |  |
|  | Digestive system diseases |  |  |  |  |  |  |  | -0.11 | -0.22 | 0.08 | -0.03 |  |
|  | Pregnancy |  |  |  |  |  |  |  | 0.05 | 0.05 | -0.01 | 0.00 |  |
|  | Perinatal diseases |  |  |  |  |  |  |  | -0.01 | 0.17 | -0.02 | 0.03 |  |
|  | External causes |  |  |  |  |  |  |  | 0.01 | 0.19 | 0.05 | 0.11 |  |
|  | Other diseases |  |  |  |  |  |  |  | 0.00 | -0.45 | 0.31 | -0.14 |  |
|  | Total |  |  |  |  |  |  |  | 0.85 | -1.33 | 1.75 | 0.88 |  |

| **Country** | **Cause of death** | **1950-55** | **1955-60** | **1960-65** | **1965-70** | **1970-75** | **1975-80** | **1980-85** | **1985-90** | **1990-95** | **1995-2000** | **2000-05** | **2005-10** |
| --- | --- | --- | --- | --- | --- | --- | --- | --- | --- | --- | --- | --- | --- |
| Venezuela | Infectious diseases |  | 0.55 | 0.07 | -0.74 | 0.42 | 0.52 |  | -0.09 |  |  |  |  |
|  | Malignant neoplasms |  | 0.02 | -0.01 | 0.03 | 0.07 | 0.26 |  | -0.12 |  |  |  |  |
|  | Cardiovascular diseases |  | -0.14 | -0.05 | -0.24 | -0.28 | 0.14 |  | -0.35 |  |  |  |  |
|  | Respiratory diseases |  | 0.06 | -0.01 | -0.42 | 0.09 | 0.51 |  | -0.05 |  |  |  |  |
|  | Digestive system diseases |  | 0.52 | 0.25 | 0.72 | 0.06 | 0.00 |  | -0.01 |  |  |  |  |
|  | Pregnancy |  | 0.03 | 0.04 | 0.07 | 0.06 | 0.05 |  | 0.00 |  |  |  |  |
|  | Perinatal diseases |  | 0.04 | -0.08 | 0.29 | -0.02 | -0.03 |  | 0.01 |  |  |  |  |
|  | External causes |  | -0.01 | -0.01 | -0.09 | -0.06 | 0.08 |  | 0.03 |  |  |  |  |
|  | Other diseases |  | 3.30 | 1.28 | 0.48 | 0.87 | 1.17 |  | 1.30 |  |  |  |  |
|  | Total |  | 4.37 | 1.48 | 0.11 | 1.20 | 2.71 |  | 0.70 |  |  |  |  |
| Yugoslavia, Former | Infectious diseases |  |  |  | 0.02 | 0.36 | 0.15 | 0.04 | 0.06 |  |  |  |  |
|  | Malignant neoplasms |  |  |  | -0.04 | -0.10 | -0.07 | -0.01 | -0.07 |  |  |  |  |
|  | Cardiovascular diseases |  |  |  | -0.17 | -0.42 | -0.43 | 0.11 | 0.49 |  |  |  |  |
|  | Respiratory diseases |  |  |  | 0.01 | 0.32 | 0.14 | 0.17 | 0.30 |  |  |  |  |
|  | Digestive system diseases |  |  |  | 0.46 | 0.04 | -0.09 | 0.04 | 0.07 |  |  |  |  |
|  | Pregnancy |  |  |  | 0.06 | 0.03 | 0.01 | 0.01 | 0.00 |  |  |  |  |
|  | Perinatal diseases |  |  |  | 1.45 | 0.05 | -0.01 | 0.07 | 0.10 |  |  |  |  |
|  | External causes |  |  |  | -0.11 | 0.06 | 0.00 | 0.08 | 0.02 |  |  |  |  |
|  | Other diseases |  |  |  | -0.50 | 2.28 | 1.35 | 0.06 | 0.43 |  |  |  |  |
|  | Total |  |  |  | 1.18 | 2.62 | 1.06 | 0.56 | 1.39 |  |  |  |  |
| Croatia | Infectious diseases |  |  |  |  |  |  |  | 0.03 | 0.02 | -0.04 | 0.05 | 0.01 |
|  | Malignant neoplasms |  |  |  |  |  |  |  | -0.13 | 0.08 | -0.18 | 0.21 | -0.05 |
|  | Cardiovascular diseases |  |  |  |  |  |  |  | **0.41** | 0.39 | -0.17 | 1.37 | 0.69 |
|  | Respiratory diseases |  |  |  |  |  |  |  | 0.23 | -0.07 | -0.01 | 0.00 | 0.17 |
|  | Digestive system diseases |  |  |  |  |  |  |  | 0.01 | 0.06 | 0.01 | 0.11 | 0.04 |
|  | Pregnancy |  |  |  |  |  |  |  | 0.00 | 0.00 | 0.00 | 0.00 | 0.00 |
|  | Perinatal diseases |  |  |  |  |  |  |  | 0.21 | 0.10 | 0.01 | 0.08 | 0.03 |
|  | External causes |  |  |  |  |  |  |  | -0.14 | 0.29 | 0.15 | 0.06 | 0.05 |
|  | Other diseases |  |  |  |  |  |  |  | 0.25 | -0.12 | 0.40 | 0.16 | -0.01 |
|  | Total |  |  |  |  |  |  |  | 0.85 | 0.73 | 0.18 | 2.04 | 0.93 |
| Serbia | Infectious diseases |  |  |  |  |  |  |  |  |  |  | 0.01 | 0.02 |
|  | Malignant neoplasms |  |  |  |  |  |  |  |  |  |  | -0.09 | -0.03 |
|  | Cardiovascular diseases |  |  |  |  |  |  |  |  |  |  | 0.57 | 1.02 |
|  | Respiratory diseases |  |  |  |  |  |  |  |  |  |  | 0.08 | -0.04 |
|  | Digestive system diseases |  |  |  |  |  |  |  |  |  |  | -0.02 | -0.01 |
|  | Pregnancy |  |  |  |  |  |  |  |  |  |  | 0.00 | 0.07 |
|  | Perinatal diseases |  |  |  |  |  |  |  |  |  |  | 0.11 | 0.00 |
|  | External causes |  |  |  |  |  |  |  |  |  |  | 0.11 | 0.07 |
|  | Other diseases |  |  |  |  |  |  |  |  |  |  | 0.29 | 0.21 |
|  | Total |  |  |  |  |  |  |  |  |  |  | 1.06 | 1.24 |

* The most positive contributing cause of death as well as age group of at least 0.10 life years was marked with green for each year. The most decreasing life expectancy of at least -0.10 life-years was marked red, respectively. ‘Other diseases’ were not considered. For each country the first of at least two following years with cardiovascular disease being the most positively contributing cause of death to the improvement in live expectancy was printed with bold letters as an indicator for the epidemiologic transition. Correspondingly, the same procedure was performed for the age group 65 years and older.

**Table S2:** Relative change in life expectancy [years] by five-year interval in men according to cause of death*

| **Country** | **Cause of death** | **1950-55** | **1955-60** | **1960-65** | **1965-70** | **1970-75** | **1975-80** | **1980-85** | **1985-90** | **1990-95** | **1995-2000** | **2000-05** | **2005-10** |
| --- | --- | --- | --- | --- | --- | --- | --- | --- | --- | --- | --- | --- | --- |
| Argentina | Infectious diseases |  |  |  |  |  |  | 0.41 | 0.01 | 0.05 |  |  |  |
|  | Malignant neoplasms |  |  |  |  |  |  | 0.21 | 0.01 | 0.06 |  |  |  |
|  | Cardiovascular diseases |  |  |  |  |  |  | **0.47** | 0.53 | 0.57 |  |  |  |
|  | Respiratory diseases |  |  |  |  |  |  | 0.28 | 0.04 | -0.15 |  |  |  |
|  | Digestive system diseases |  |  |  |  |  |  | 0.10 | 0.08 | 0.08 |  |  |  |
|  | Perinatal diseases |  |  |  |  |  |  | 0.19 | 0.06 | 0.14 |  |  |  |
|  | External causes |  |  |  |  |  |  | 0.34 | -0.02 | -0.10 |  |  |  |
|  | Other diseases |  |  |  |  |  |  | 0.65 | -0.02 | -0.19 |  |  |  |
|  | Total |  |  |  |  |  |  | 2.65 | 0.69 | 0.46 |  |  |  |
| Armenia | Infectious diseases |  |  |  |  |  |  |  | 0.04 | 0.09 | 0.05 |  |  |
|  | Malignant neoplasms |  |  |  |  |  |  |  | -0.25 | 0.39 | -0.05 |  |  |
|  | Cardiovascular diseases |  |  |  |  |  |  |  | -0.76 | 0.09 | 1.41 |  |  |
|  | Respiratory diseases |  |  |  |  |  |  |  | 0.62 | 0.19 | 0.35 |  |  |
|  | Digestive system diseases |  |  |  |  |  |  |  | 0.02 | -0.04 | 0.21 |  |  |
|  | Perinatal diseases |  |  |  |  |  |  |  | -0.06 | -0.02 | -0.04 |  |  |
|  | External causes |  |  |  |  |  |  |  | -0.80 | -0.12 | 1.21 |  |  |
|  | Other diseases |  |  |  |  |  |  |  | 0.03 | -0.16 | 0.25 |  |  |
|  | Total |  |  |  |  |  |  |  | -1.16 | 0.42 | 3.38 |  |  |
| Australia | Infectious diseases | 0.38 | 0.17 | 0.08 | -0.04 | 0.05 | 0.06 | 0.00 | -0.09 | -0.04 | 0.10 |  |  |
|  | Malignant neoplasms | -0.08 | -0.09 | -0.11 | -0.14 | -0.05 | 0.00 | -0.01 | 0.10 | 0.14 | 0.36 |  |  |
|  | Cardiovascular diseases | -0.01 | -0.02 | -0.29 | **0.15** | 0.65 | 1.22 | 0.98 | 1.03 | 0.88 | 1.08 |  |  |
|  | Respiratory diseases | 0.07 | -0.03 | -0.07 | -0.08 | 0.19 | 0.29 | 0.08 | 0.09 | 0.18 | 0.19 |  |  |
|  | Digestive system diseases | 0.11 | 0.08 | 0.05 | 0.10 | -0.05 | 0.02 | 0.06 | 0.06 | 0.09 | 0.04 |  |  |
|  | Perinatal diseases | 0.15 | 0.10 | 0.07 | 0.07 | 0.18 | 0.24 | 0.07 | 0.03 | 0.06 | -0.01 |  |  |
|  | External causes | 0.03 | 0.08 | -0.06 | -0.04 | 0.16 | 0.24 | 0.27 | 0.15 | 0.21 | -0.01 |  |  |
|  | Other diseases | 0.29 | 0.27 | 0.12 | 0.10 | -0.02 | 0.20 | -0.04 | 0.10 | -0.06 | 0.14 |  |  |
|  | Total | 0.94 | 0.55 | -0.20 | 0.12 | 1.12 | 2.26 | 1.41 | 1.47 | 1.47 | 1.88 |  |  |
| Austria | Infectious diseases |  | 0.20 | 0.16 | 0.13 | 0.11 | 0.04 | 0.04 | -0.01 | 0.00 | 0.05 | -0.04 | 0.01 |
|  | Malignant neoplasms |  | -0.02 | -0.01 | 0.05 | 0.07 | 0.05 | 0.11 | 0.08 | 0.25 | 0.24 | 0.20 | 0.28 |
|  | Cardiovascular diseases |  | -0.03 | 0.09 | -0.32 | 0.16 | 0.19 | **0.49** | 0.82 | 0.43 | 0.77 | 1.50 | 0.40 |
|  | Respiratory diseases |  | 0.21 | 0.13 | -0.17 | 0.33 | 0.21 | 0.08 | 0.13 | 0.13 | -0.01 | 0.02 | 0.12 |
|  | Digestive system diseases |  | 0.05 | 0.09 | -0.07 | 0.02 | 0.14 | 0.16 | 0.15 | 0.11 | 0.18 | 0.10 | 0.09 |
|  | Perinatal diseases |  | 0.34 | 0.22 | 0.22 | 0.27 | 0.35 | 0.12 | 0.17 | 0.03 | 0.03 | -0.01 | 0.04 |
|  | External causes |  | -0.01 | 0.18 | -0.31 | 0.18 | 0.19 | 0.30 | 0.39 | 0.22 | 0.30 | 0.27 | 0.23 |
|  | Other diseases |  | 0.21 | 0.15 | 0.17 | 0.13 | 0.15 | 0.16 | 0.09 | 0.21 | 0.10 | -0.51 | -0.17 |
|  | Total |  | 0.95 | 1.00 | -0.30 | 1.26 | 1.31 | 1.45 | 1.82 | 1.38 | 1.65 | 1.53 | 1.00 |
| Azerbaijan | Infectious diseases |  |  |  |  |  |  |  | 0.10 | -0.14 | 0.27 |  |  |
|  | Malignant neoplasms |  |  |  |  |  |  |  | 0.25 | 0.23 | 0.07 |  |  |
|  | Cardiovascular diseases |  |  |  |  |  |  |  | -0.08 | -0.61 | 0.62 |  |  |
|  | Respiratory diseases |  |  |  |  |  |  |  | 0.15 | 0.02 | 1.00 |  |  |
|  | Digestive system diseases |  |  |  |  |  |  |  | 0.06 | -0.19 | 0.16 |  |  |
|  | Perinatal diseases |  |  |  |  |  |  |  | 0.07 | 0.04 | 0.03 |  |  |
|  | External causes |  |  |  |  |  |  |  | -0.21 | -0.78 | 1.50 |  |  |
|  | Other diseases |  |  |  |  |  |  |  | -0.11 | -0.29 | 0.34 |  |  |
|  | Total |  |  |  |  |  |  |  | 0.24 | -1.72 | 3.99 |  |  |
| Belarus | Infectious diseases |  |  |  |  |  |  |  | 0.02 | -0.05 |  |  |  |
|  | Malignant neoplasms |  |  |  |  |  |  |  | -0.26 | -0.14 |  |  |  |
|  | Cardiovascular diseases |  |  |  |  |  |  |  | 0.16 | -1.13 |  |  |  |
|  | Respiratory diseases |  |  |  |  |  |  |  | 0.32 | -0.04 |  |  |  |
|  | Digestive system diseases |  |  |  |  |  |  |  | 0.04 | -0.07 |  |  |  |
|  | Perinatal diseases |  |  |  |  |  |  |  | 0.03 | -0.03 |  |  |  |
|  | External causes |  |  |  |  |  |  |  | -0.75 | -1.07 |  |  |  |
|  | Other diseases |  |  |  |  |  |  |  | -0.29 | -0.50 |  |  |  |
|  | Total |  |  |  |  |  |  |  | -0.73 | -3.03 |  |  |  |
| Belgium | Infectious diseases |  | 0.31 | 0.19 | 0.03 | 0.10 | 0.04 | 0.03 | -0.02 | -0.07 |  |  |  |
|  | Malignant neoplasms |  | -0.22 | -0.12 | -0.11 | -0.08 | -0.12 | 0.08 | 0.19 | 0.13 |  |  |  |
|  | Cardiovascular diseases |  | 0.01 | -0.38 | 0.05 | **0.37** | 0.46 | 0.54 | 0.94 | 0.40 |  |  |  |
|  | Respiratory diseases |  | 0.28 | 0.09 | -0.03 | 0.20 | 0.10 | -0.02 | 0.10 | 0.02 |  |  |  |
|  | Digestive system diseases |  | 0.05 | 0.02 | 0.03 | 0.01 | 0.02 | 0.03 | 0.07 | -0.01 |  |  |  |
|  | Perinatal diseases |  | 0.35 | 0.22 | 0.21 | 0.21 | 0.20 | 0.17 | 0.03 | 0.07 |  |  |  |
|  | External causes |  | -0.05 | -0.08 | -0.14 | 0.18 | -0.07 | 0.25 | 0.12 | -0.01 |  |  |  |
|  | Other diseases |  | 0.43 | 0.40 | 0.02 | 0.09 | 0.47 | 0.10 | 0.11 | 0.41 |  |  |  |
|  | Total |  | 1.16 | 0.34 | 0.06 | 1.07 | 1.10 | 1.17 | 1.54 | 0.93 |  |  |  |

| **Country** | **Cause of death** | **1950-55** | **1955-60** | **1960-65** | **1965-70** | **1970-75** | **1975-80** | **1980-85** | **1985-90** | **1990-95** | **1995-2000** | **2000-05** | **2005-10** |
| --- | --- | --- | --- | --- | --- | --- | --- | --- | --- | --- | --- | --- | --- |
| Bulgaria | Infectious diseases |  |  |  | 0.03 | 0.15 | 0.09 | 0.01 | 0.02 | -0.04 | 0.00 | 0.04 | 0.03 |
|  | Malignant neoplasms |  |  |  | 0.11 | 0.05 | -0.01 | -0.13 | -0.10 | -0.11 | 0.17 | -0.16 | 0.14 |
|  | Cardiovascular diseases |  |  |  | -0.77 | -0.57 | -0.52 | -0.59 | -0.26 | -0.51 | 0.07 | 0.20 | 0.68 |
|  | Respiratory diseases |  |  |  | -0.59 | 0.65 | 0.39 | 0.32 | 0.14 | 0.10 | 0.20 | 0.13 | 0.01 |
|  | Digestive system diseases |  |  |  | 0.08 | -0.03 | -0.04 | -0.09 | 0.00 | -0.12 | 0.15 | -0.02 | 0.00 |
|  | Perinatal diseases |  |  |  | 0.49 | -0.06 | 0.12 | 0.09 | 0.05 | -0.05 | 0.05 | 0.07 | -0.04 |
|  | External causes |  |  |  | -0.19 | 0.07 | -0.11 | -0.02 | 0.00 | -0.15 | 0.41 | 0.17 | 0.29 |
|  | Other diseases |  |  |  | 0.01 | -0.08 | -0.08 | 0.24 | -0.06 | -0.06 | 0.05 | 0.25 | 0.18 |
|  | Total |  |  |  | -0.82 | 0.19 | -0.16 | -0.17 | -0.20 | -0.93 | 1.10 | 0.67 | 1.28 |
| Canada | Infectious diseases | 0.46 | 0.16 | 0.09 | 0.00 | 0.04 | 0.05 | -0.02 | -0.15 | -0.05 | 0.15 | -0.02 |  |
|  | Malignant neoplasms | -0.13 | -0.04 | -0.09 | -0.14 | -0.05 | -0.05 | -0.05 | 0.02 | 0.27 | 0.20 | 0.28 |  |
|  | Cardiovascular diseases | 0.05 | -0.06 | **0.25** | 0.42 | 0.26 | 0.90 | 0.89 | 0.88 | 0.66 | 0.66 | 0.70 |  |
|  | Respiratory diseases | 0.20 | 0.01 | 0.09 | -0.09 | 0.08 | 0.20 | -0.01 | 0.04 | 0.10 | 0.14 | 0.12 |  |
|  | Digestive system diseases | 0.15 | 0.02 | 0.05 | 0.05 | -0.05 | 0.06 | 0.13 | 0.04 | 0.06 | 0.04 | 0.02 |  |
|  | Perinatal diseases | 0.27 | 0.10 | 0.10 | 0.30 | 0.29 | 0.16 | 0.09 | 0.04 | 0.02 | 0.00 | -0.02 |  |
|  | External causes | -0.05 | 0.04 | -0.14 | -0.04 | 0.01 | 0.23 | 0.51 | 0.18 | 0.25 | 0.14 | 0.08 |  |
|  | Other diseases | 0.37 | 0.24 | 0.14 | 0.11 | -0.04 | 0.12 | -0.05 | -0.06 | 0.04 | 0.06 | 0.02 |  |
|  | Total | 1.34 | 0.46 | 0.48 | 0.62 | 0.54 | 1.66 | 1.49 | 0.99 | 1.35 | 1.39 | 1.19 |  |
| Chile | Infectious diseases |  | 0.07 | 0.40 | -0.36 | 0.69 | 0.86 | 0.36 | 0.12 |  |  |  |  |
|  | Malignant neoplasms |  | -0.12 | 0.04 | -0.06 | 0.17 | 0.09 | 0.07 | 0.03 |  |  |  |  |
|  | Cardiovascular diseases |  | 0.22 | 0.00 | 0.01 | 0.83 | -0.07 | 0.15 | 0.38 |  |  |  |  |
|  | Respiratory diseases |  | 0.00 | 1.14 | 0.20 | 1.21 | 1.05 | 0.00 | 0.16 |  |  |  |  |
|  | Digestive system diseases |  | -0.32 | -0.31 | 1.13 | 0.42 | -0.01 | 0.09 | 0.26 |  |  |  |  |
|  | Perinatal diseases |  | 0.26 | 0.51 | 1.38 | -0.16 | 0.54 | 0.35 | 0.10 |  |  |  |  |
|  | External causes |  | 0.03 | -0.02 | -0.18 | 0.78 | -0.04 | 0.11 | 0.11 |  |  |  |  |
|  | Other diseases |  | 0.63 | 0.48 | 0.36 | -0.23 | 0.57 | 0.49 | 0.46 |  |  |  |  |
|  | Total |  | 0.76 | 2.24 | 2.48 | 3.69 | 2.98 | 1.61 | 1.63 |  |  |  |  |
| China,select rural areas | Infectious diseases |  |  |  |  |  |  |  |  | 0.24 | 0.19 |  |  |
|  | Malignant neoplasms |  |  |  |  |  |  |  |  | 0.12 | -0.05 |  |  |
|  | Cardiovascular diseases |  |  |  |  |  |  |  |  | 0.18 | -0.19 |  |  |
|  | Respiratory diseases |  |  |  |  |  |  |  |  | 0.27 | 0.69 |  |  |
|  | Digestive system diseases |  |  |  |  |  |  |  |  | 0.12 | 0.14 |  |  |
|  | Perinatal diseases |  |  |  |  |  |  |  |  | -0.05 | 0.20 |  |  |
|  | External causes |  |  |  |  |  |  |  |  | 0.00 | 0.26 |  |  |
|  | Other diseases |  |  |  |  |  |  |  |  | 0.06 | 0.08 |  |  |
|  | Total |  |  |  |  |  |  |  |  | 0.94 | 1.31 |  |  |
| China,select urban | Infectious diseases |  |  |  |  |  |  |  |  | 0.18 | 0.12 |  |  |
| and rural areas | Malignant neoplasms |  |  |  |  |  |  |  |  | 0.17 | -0.05 |  |  |
|  | Cardiovascular diseases |  |  |  |  |  |  |  |  | 0.15 | 0.07 |  |  |
|  | Respiratory diseases |  |  |  |  |  |  |  |  | 0.27 | 0.51 |  |  |
|  | Digestive system diseases |  |  |  |  |  |  |  |  | 0.10 | 0.10 |  |  |
|  | Perinatal diseases |  |  |  |  |  |  |  |  | 0.02 | 0.19 |  |  |
|  | External causes |  |  |  |  |  |  |  |  | 0.03 | 0.20 |  |  |
|  | Other diseases |  |  |  |  |  |  |  |  | 0.04 | 0.08 |  |  |
|  | Total |  |  |  |  |  |  |  |  | 0.97 | 1.21 |  |  |
| China,select urban areas | Infectious diseases |  |  |  |  |  |  |  |  | 0.13 | 0.06 |  |  |
|  | Malignant neoplasms |  |  |  |  |  |  |  |  | 0.21 | -0.04 |  |  |
|  | Cardiovascular diseases |  |  |  |  |  |  |  |  | 0.14 | 0.24 |  |  |
|  | Respiratory diseases |  |  |  |  |  |  |  |  | 0.25 | 0.37 |  |  |
|  | Digestive system diseases |  |  |  |  |  |  |  |  | 0.09 | 0.07 |  |  |
|  | Perinatal diseases |  |  |  |  |  |  |  |  | 0.11 | 0.19 |  |  |
|  | External causes |  |  |  |  |  |  |  |  | 0.06 | 0.16 |  |  |
|  | Other diseases |  |  |  |  |  |  |  |  | 0.01 | 0.09 |  |  |
|  | Total |  |  |  |  |  |  |  |  | 1.01 | 1.12 |  |  |
| Costa Rica | Infectious diseases |  |  |  | -1.32 | 1.62 | 0.83 | 0.04 | 0.09 | 0.05 |  |  |  |
|  | Malignant neoplasms |  |  |  | 0.28 | 0.01 | -0.04 | -0.47 | 0.39 | 0.22 |  |  |  |
|  | Cardiovascular diseases |  |  |  | -0.39 | 0.63 | 0.04 | -0.58 | 0.60 | -0.04 |  |  |  |
|  | Respiratory diseases |  |  |  | -0.22 | 0.72 | 0.30 | -0.17 | 0.19 | -0.05 |  |  |  |
|  | Digestive system diseases |  |  |  | 1.91 | 0.10 | 0.10 | 0.09 | -0.07 | -0.15 |  |  |  |
|  | Perinatal diseases |  |  |  | 0.72 | 0.24 | 0.14 | -0.04 | 0.20 | -0.02 |  |  |  |
|  | External causes |  |  |  | -0.08 | -0.25 | 0.24 | 0.37 | 0.09 | -0.16 |  |  |  |
|  | Other diseases |  |  |  | 0.26 | 0.77 | 0.30 | 0.79 | 0.03 | 0.11 |  |  |  |
|  | Total |  |  |  | 1.15 | 3.84 | 1.91 | 0.03 | 1.52 | -0.04 |  |  |  |
| Cuba | Infectious diseases |  |  |  |  | 0.75 | 0.20 | 0.07 | 0.06 | -0.05 |  |  |  |
|  | Malignant neoplasms |  |  |  |  | 0.16 | 0.09 | -0.01 | -0.02 | 0.01 |  |  |  |
|  | Cardiovascular diseases |  |  |  |  | 0.27 | 0.09 | 0.16 | **0.25** | 0.14 |  |  |  |
|  | Respiratory diseases |  |  |  |  | 0.19 | 0.18 | 0.14 | 0.23 | -0.22 |  |  |  |
|  | Digestive system diseases |  |  |  |  | 0.10 | -0.01 | 0.01 | -0.06 | 0.01 |  |  |  |
|  | Perinatal diseases |  |  |  |  | 0.24 | 0.20 | 0.22 | 0.18 | 0.07 |  |  |  |
|  | External causes |  |  |  |  | -0.09 | -0.20 | -0.08 | -0.19 | 0.10 |  |  |  |
|  | Other diseases |  |  |  |  | 0.33 | -0.01 | 0.02 | -0.10 | -0.06 |  |  |  |
|  | Total |  |  |  |  | 1.96 | 0.53 | 0.53 | 0.34 | 0.00 |  |  |  |

| **Country** | **Cause of death** | **1950-55** | **1955-60** | **1960-65** | **1965-70** | **1970-75** | **1975-80** | **1980-85** | **1985-90** | **1990-95** | **1995-2000** | **2000-05** | **2005-10** |
| --- | --- | --- | --- | --- | --- | --- | --- | --- | --- | --- | --- | --- | --- |
| Czechoslovakia, Former | Infectious diseases |  | 0.54 | 0.29 | 0.14 | 0.07 | 0.04 | 0.04 | 0.00 |  |  |  |  |
|  | Malignant neoplasms |  | -0.19 | -0.19 | -0.06 | -0.09 | -0.08 | -0.13 | -0.10 |  |  |  |  |
|  | Cardiovascular diseases |  | 0.31 | -0.12 | -0.82 | 0.07 | -0.24 | -0.22 | 0.12 |  |  |  |  |
|  | Respiratory diseases |  | 0.05 | 0.07 | -0.28 | 0.20 | 0.16 | 0.13 | 0.24 |  |  |  |  |
|  | Digestive system diseases |  | 0.13 | -0.02 | -0.08 | -0.05 | -0.04 | 0.04 | -0.08 |  |  |  |  |
|  | Perinatal diseases |  | 0.32 | -0.14 | 0.07 | 0.01 | 0.16 | 0.17 | 0.09 |  |  |  |  |
|  | External causes |  | -0.12 | 0.02 | -0.25 | 0.33 | 0.16 | 0.13 | -0.06 |  |  |  |  |
|  | Other diseases |  | 0.44 | 0.00 | 0.11 | 0.08 | -0.09 | 0.09 | 0.13 |  |  |  |  |
|  | Total |  | 1.48 | -0.08 | -1.17 | 0.63 | 0.07 | 0.26 | 0.34 |  |  |  |  |
| Czech Republic | Infectious diseases |  |  |  |  |  |  |  |  | 0.01 | 0.01 | -0.01 | -0.06 |
|  | Malignant neoplasms |  |  |  |  |  |  |  |  | 0.19 | 0.31 | 0.34 | 0.47 |
|  | Cardiovascular diseases |  |  |  |  |  |  |  |  | **0.90** | 1.08 | 0.80 | 0.72 |
|  | Respiratory diseases |  |  |  |  |  |  |  |  | 0.16 | 0.02 | -0.01 | 0.00 |
|  | Digestive system diseases |  |  |  |  |  |  |  |  | 0.11 | 0.04 | -0.01 | 0.08 |
|  | Perinatal diseases |  |  |  |  |  |  |  |  | 0.17 | 0.11 | 0.02 | 0.04 |
|  | External causes |  |  |  |  |  |  |  |  | 0.00 | 0.23 | 0.18 | 0.22 |
|  | Other diseases |  |  |  |  |  |  |  |  | 0.33 | 0.09 | -0.08 | -0.02 |
|  | Total |  |  |  |  |  |  |  |  | 1.87 | 1.89 | 1.25 | 1.45 |
| Slovakia | Infectious diseases |  |  |  |  |  |  |  |  |  | 0.00 | -0.01 | -0.01 |
|  | Malignant neoplasms |  |  |  |  |  |  |  |  |  | 0.02 | 0.31 | 0.25 |
|  | Cardiovascular diseases |  |  |  |  |  |  |  |  |  | **0.43** | 0.44 | 0.58 |
|  | Respiratory diseases |  |  |  |  |  |  |  |  |  | 0.20 | -0.05 | 0.04 |
|  | Digestive system diseases |  |  |  |  |  |  |  |  |  | -0.08 | 0.11 | 0.02 |
|  | Perinatal diseases |  |  |  |  |  |  |  |  |  | 0.16 | 0.06 | 0.04 |
|  | External causes |  |  |  |  |  |  |  |  |  | 0.14 | 0.15 | 0.19 |
|  | Other diseases |  |  |  |  |  |  |  |  |  | -0.08 | 0.01 | 0.10 |
|  | Total |  |  |  |  |  |  |  |  |  | 0.79 | 1.03 | 1.21 |
| Denmark | Infectious diseases |  | 0.08 | 0.05 | -0.01 | 0.02 | 0.04 | -0.03 | -0.08 | -0.04 | 0.14 | -0.03 |  |
|  | Malignant neoplasms |  | -0.13 | -0.05 | 0.00 | -0.01 | -0.16 | -0.04 | 0.08 | 0.03 | 0.20 | 0.22 |  |
|  | Cardiovascular diseases |  | -0.08 | -0.55 | **0.47** | -0.08 | 0.23 | 0.51 | 0.46 | 0.69 | 0.76 | 0.73 |  |
|  | Respiratory diseases |  | 0.00 | -0.03 | -0.10 | -0.10 | 0.08 | 0.03 | 0.06 | -0.04 | 0.10 | 0.11 |  |
|  | Digestive system diseases |  | 0.10 | 0.00 | 0.09 | -0.04 | 0.01 | -0.03 | -0.02 | -0.07 | -0.01 | 0.04 |  |
|  | Perinatal diseases |  | 0.15 | 0.16 | 0.25 | 0.22 | 0.20 | 0.05 | 0.00 | 0.02 | 0.02 | -0.01 |  |
|  | External causes |  | 0.18 | -0.06 | -0.17 | 0.21 | -0.12 | 0.10 | 0.18 | 0.16 | 0.18 | 0.27 |  |
|  | Other diseases |  | 0.01 | 0.14 | 0.10 | 0.08 | -0.17 | -0.06 | -0.16 | -0.13 | 0.34 | -0.06 |  |
|  | Total |  | 0.32 | -0.35 | 0.62 | 0.31 | 0.11 | 0.53 | 0.51 | 0.62 | 1.73 | 1.29 |  |
| Estonia | Infectious diseases |  |  |  |  |  |  |  | 0.00 | -0.05 | 0.03 | 0.04 | 0.01 |
|  | Malignant neoplasms |  |  |  |  |  |  |  | -0.11 | 0.03 | 0.22 | 0.03 | 0.22 |
|  | Cardiovascular diseases |  |  |  |  |  |  |  | 0.32 | -0.49 | 0.98 | 0.71 | 1.29 |
|  | Respiratory diseases |  |  |  |  |  |  |  | 0.13 | -0.19 | 0.09 | 0.15 | 0.19 |
|  | Digestive system diseases |  |  |  |  |  |  |  | 0.03 | -0.09 | -0.10 | -0.05 | 0.12 |
|  | Perinatal diseases |  |  |  |  |  |  |  | 0.08 | -0.02 | 0.23 | 0.11 | 0.06 |
|  | External causes |  |  |  |  |  |  |  | -0.84 | -1.74 | 1.41 | 1.00 | 1.08 |
|  | Other diseases |  |  |  |  |  |  |  | 0.00 | -0.25 | 0.15 | -0.07 | 0.49 |
|  | Total |  |  |  |  |  |  |  | -0.39 | -2.81 | 3.00 | 1.92 | 3.46 |
| Finland | Infectious diseases |  | 0.47 | 0.37 | 0.11 | 0.06 | 0.06 | 0.04 | 0.01 | 0.01 | 0.02 | 0.01 | 0.01 |
|  | Malignant neoplasms |  | -0.03 | 0.08 | -0.02 | 0.13 | 0.11 | 0.21 | 0.22 | 0.17 | 0.24 | 0.21 | 0.19 |
|  | Cardiovascular diseases |  | 0.11 | -0.76 | 0.08 | **0.60** | 0.82 | 0.59 | 0.89 | 1.04 | 0.85 | 0.79 | 0.54 |
|  | Respiratory diseases |  | 0.15 | 0.15 | -0.18 | 0.19 | 0.18 | 0.12 | 0.07 | 0.07 | 0.05 | 0.37 | 0.17 |
|  | Digestive system diseases |  | 0.11 | 0.12 | 0.07 | -0.01 | 0.03 | 0.04 | -0.14 | 0.03 | 0.02 | -0.14 | 0.01 |
|  | Perinatal diseases |  | 0.24 | 0.10 | 0.25 | 0.20 | 0.17 | 0.06 | -0.02 | 0.04 | 0.03 | 0.01 | 0.03 |
|  | External causes |  | -0.05 | -0.07 | -0.15 | 0.14 | 0.43 | 0.11 | -0.37 | 0.45 | 0.25 | 0.11 | 0.30 |
|  | Other diseases |  | 0.23 | 0.16 | 0.16 | 0.05 | 0.11 | -0.01 | 0.00 | 0.08 | -0.16 | 0.05 | 0.05 |
|  | Total |  | 1.23 | 0.15 | 0.30 | 1.36 | 1.90 | 1.15 | 0.67 | 1.89 | 1.31 | 1.41 | 1.29 |
| France | Infectious diseases |  | 0.29 | 0.23 | 0.11 | 0.04 | 0.10 | 0.03 | -0.17 | -0.08 | 0.23 | 0.03 |  |
|  | Malignant neoplasms |  | -0.16 | -0.17 | -0.03 | -0.21 | -0.08 | -0.06 | 0.12 | 0.26 | 0.18 | 0.37 |  |
|  | Cardiovascular diseases |  | 0.35 | 0.10 | 0.03 | 0.17 | **0.50** | 0.35 | 0.78 | 0.39 | 0.25 | 0.53 |  |
|  | Respiratory diseases |  | 0.28 | 0.22 | -0.12 | 0.16 | 0.13 | 0.06 | 0.09 | 0.03 | 0.09 | 0.16 |  |
|  | Digestive system diseases |  | 0.09 | -0.06 | 0.00 | 0.00 | 0.12 | 0.20 | 0.21 | 0.09 | 0.05 | 0.09 |  |
|  | Perinatal diseases |  | 0.37 | 0.18 | 0.23 | 0.22 | 0.11 | 0.06 | 0.01 | 0.00 | -0.01 | 0.01 |  |
|  | External causes |  | 0.03 | -0.14 | -0.15 | 0.13 | 0.06 | 0.19 | 0.19 | 0.28 | 0.19 | 0.34 |  |
|  | Other diseases |  | 0.61 | 0.21 | 0.52 | 0.32 | 0.26 | 0.14 | 0.32 | 0.26 | -0.19 | 0.10 |  |
|  | Total |  | 1.85 | 0.59 | 0.60 | 0.83 | 1.20 | 0.97 | 1.56 | 1.24 | 0.80 | 1.64 |  |
| Germany, | Infectious diseases |  | 0.16 | 0.14 | 0.04 | 0.05 | 0.08 | 0.03 | -0.05 |  |  |  |  |
| Former Federal Republic | Malignant neoplasms |  | -0.10 | -0.10 | -0.02 | -0.01 | 0.03 | 0.02 | 0.00 |  |  |  |  |
|  | Cardiovascular diseases |  | -0.10 | -0.19 | -0.19 | 0.19 | 0.12 | **0.52** | 0.68 |  |  |  |  |
|  | Respiratory diseases |  | 0.12 | 0.28 | -0.23 | 0.24 | 0.17 | 0.05 | 0.10 |  |  |  |  |
|  | Digestive system diseases |  | -0.04 | -0.01 | 0.02 | -0.04 | 0.14 | 0.20 | 0.08 |  |  |  |  |
|  | Perinatal diseases |  | 0.37 | 0.38 | 0.10 | 0.30 | 0.42 | 0.15 | 0.09 |  |  |  |  |
|  | External causes |  | 0.10 | 0.04 | -0.15 | 0.28 | 0.26 | 0.42 | 0.23 |  |  |  |  |
|  | Other diseases |  | 0.22 | 0.25 | 0.07 | 0.03 | 0.41 | 0.24 | -0.05 |  |  |  |  |
|  | Total |  | 0.73 | 0.79 | -0.36 | 1.04 | 1.63 | 1.64 | 1.08 |  |  |  |  |

| **Country** | **Cause of death** | **1950-55** | **1955-60** | **1960-65** | **1965-70** | **1970-75** | **1975-80** | **1980-85** | **1985-90** | **1990-95** | **1995-2000** | **2000-05** | **2005-10** |
| --- | --- | --- | --- | --- | --- | --- | --- | --- | --- | --- | --- | --- | --- |
| Germany | Infectious diseases |  |  |  |  |  |  |  |  | -0.01 | 0.03 | 0.01 | -0.01 |
|  | Malignant neoplasms |  |  |  |  |  |  |  |  | 0.12 | 0.31 | 0.32 | 0.26 |
|  | Cardiovascular diseases |  |  |  |  |  |  |  |  | **0.62** | 0.82 | 0.84 | 0.80 |
|  | Respiratory diseases |  |  |  |  |  |  |  |  | 0.09 | 0.13 | 0.05 | 0.04 |
|  | Digestive system diseases |  |  |  |  |  |  |  |  | 0.05 | 0.11 | 0.06 | 0.10 |
|  | Perinatal diseases |  |  |  |  |  |  |  |  | 0.03 | 0.02 | 0.00 | 0.02 |
|  | External causes |  |  |  |  |  |  |  |  | 0.19 | 0.22 | 0.20 | 0.13 |
|  | Other diseases |  |  |  |  |  |  |  |  | 0.15 | 0.19 | 0.10 | -0.11 |
|  | Total |  |  |  |  |  |  |  |  | 1.25 | 1.83 | 1.58 | 1.23 |
| Greece | Infectious diseases |  |  |  | -0.05 | 0.26 | 0.19 | 0.06 | 0.04 | -0.04 | 0.03 | 0.02 | -0.02 |
|  | Malignant neoplasms |  |  |  | -0.02 | -0.26 | -0.06 | 0.01 | 0.02 | -0.06 | 0.05 | 0.15 | 0.16 |
|  | Cardiovascular diseases |  |  |  | -0.21 | -0.16 | -0.29 | -0.18 | 0.03 | **0.29** | 0.34 | 0.65 | 0.76 |
|  | Respiratory diseases |  |  |  | -0.13 | 0.34 | 0.36 | 0.19 | 0.07 | 0.00 | -0.11 | 0.09 | -0.05 |
|  | Digestive system diseases |  |  |  | 0.18 | 0.16 | 0.05 | 0.10 | 0.13 | 0.03 | 0.04 | 0.02 | 0.01 |
|  | Perinatal diseases |  |  |  | 0.47 | 0.09 | 0.19 | 0.21 | 0.29 | 0.04 | 0.09 | 0.08 | 0.03 |
|  | External causes |  |  |  | 0.10 | -0.05 | -0.02 | -0.06 | 0.04 | 0.04 | 0.06 | 0.12 | 0.16 |
|  | Other diseases |  |  |  | 0.82 | 0.48 | 0.47 | 0.20 | 0.28 | 0.14 | 0.20 | 0.23 | -0.04 |
|  | Total |  |  |  | 1.16 | 0.85 | 0.89 | 0.53 | 0.89 | 0.45 | 0.70 | 1.36 | 1.00 |
| Hong Kong SAR | Infectious diseases |  |  | 0.90 | 0.42 | 0.72 | 0.30 | 0.17 | 0.00 | 0.08 | 0.14 | -0.01 | 0.04 |
|  | Malignant neoplasms |  |  | -0.44 | -0.18 | -0.09 | -0.06 | 0.20 | 0.03 | 0.32 | 0.14 | 0.42 | 0.44 |
|  | Cardiovascular diseases |  |  | -0.12 | 0.66 | 0.31 | 0.27 | **1.07** | 0.25 | 0.53 | 0.38 | 0.29 | 0.29 |
|  | Respiratory diseases |  |  | 1.08 | -0.57 | 0.22 | 0.30 | 0.55 | 0.00 | 0.17 | 0.50 | 0.12 | 0.06 |
|  | Digestive system diseases |  |  | 0.55 | 0.00 | 0.20 | 0.19 | 0.17 | 0.04 | 0.06 | 0.11 | 0.09 | 0.06 |
|  | Perinatal diseases |  |  | 0.15 | 0.40 | 0.21 | 0.08 | 0.16 | 0.09 | 0.08 | 0.09 | 0.02 | 0.02 |
|  | External causes |  |  | -0.10 | 0.12 | -0.05 | 0.03 | 0.47 | -0.03 | 0.17 | -0.06 | 0.02 | 0.24 |
|  | Other diseases |  |  | 0.80 | 0.23 | 0.40 | 0.50 | 0.76 | 0.11 | 0.23 | -0.08 | 0.03 | 0.01 |
|  | Total |  |  | 2.82 | 1.09 | 1.92 | 1.62 | 3.55 | 0.49 | 1.63 | 1.22 | 0.98 | 1.15 |
| Hungary | Infectious diseases |  | 0.31 | 0.29 | 0.07 | 0.17 | 0.10 | 0.04 | -0.01 | 0.01 | 0.05 | 0.04 | 0.01 |
|  | Malignant neoplasms |  | -0.17 | -0.13 | -0.15 | -0.20 | -0.24 | -0.22 | -0.29 | -0.16 | 0.02 | 0.37 | 0.20 |
|  | Cardiovascular diseases |  | -0.21 | -0.32 | -0.73 | 0.06 | -0.68 | -0.22 | **0.22** | 0.28 | 0.55 | 0.59 | 0.75 |
|  | Respiratory diseases |  | 0.34 | 0.80 | -0.08 | 0.14 | -0.17 | 0.09 | 0.12 | 0.06 | 0.14 | -0.02 | 0.04 |
|  | Digestive system diseases |  | 0.23 | 0.15 | 0.12 | -0.11 | -0.25 | -0.24 | -0.20 | -0.47 | 0.32 | 0.34 | 0.28 |
|  | Perinatal diseases |  | 0.25 | 0.01 | 0.12 | 0.04 | 0.52 | 0.14 | 0.22 | 0.20 | 0.10 | 0.11 | 0.05 |
|  | External causes |  | 0.46 | -0.05 | -0.39 | -0.02 | -0.20 | -0.12 | -0.05 | 0.37 | 0.29 | 0.29 | 0.38 |
|  | Other diseases |  | 0.47 | 0.44 | 0.27 | 0.03 | 0.06 | 0.00 | 0.05 | 0.03 | 0.19 | -0.07 | 0.12 |
|  | Total |  | 1.67 | 1.19 | -0.77 | 0.12 | -0.85 | -0.52 | 0.07 | 0.31 | 1.66 | 1.65 | 1.84 |
| Ireland | Infectious diseases | 1.25 | 0.41 | 0.14 | 0.05 | 0.08 | 0.05 | 0.04 | 0.02 | 0.00 | 0.03 | 0.04 |  |
|  | Malignant neoplasms | -0.09 | -0.06 | -0.07 | -0.17 | -0.07 | 0.06 | -0.08 | 0.05 | 0.09 | 0.22 | 0.33 |  |
|  | Cardiovascular diseases | -0.14 | 0.02 | -0.20 | 0.02 | -0.06 | 0.19 | **0.44** | 0.86 | 0.59 | 0.85 | 1.32 |  |
|  | Respiratory diseases | 0.63 | -0.17 | -0.07 | -0.17 | 0.14 | 0.30 | 0.11 | 0.18 | 0.10 | 0.12 | 0.45 |  |
|  | Digestive system diseases | 0.19 | 0.16 | 0.03 | 0.15 | 0.00 | 0.00 | 0.05 | 0.03 | 0.00 | -0.06 | 0.04 |  |
|  | Perinatal diseases | 0.25 | 0.24 | 0.28 | 0.38 | 0.07 | 0.24 | 0.10 | 0.04 | 0.01 | 0.02 | 0.05 |  |
|  | External causes | -0.13 | 0.03 | -0.08 | -0.26 | -0.15 | -0.09 | 0.32 | -0.04 | 0.04 | -0.03 | 0.25 |  |
|  | Other diseases | 0.85 | 0.46 | 0.29 | 0.51 | 0.17 | -0.03 | 0.09 | 0.03 | 0.13 | -0.11 | 0.22 |  |
|  | Total | 2.80 | 1.09 | 0.33 | 0.52 | 0.17 | 0.74 | 1.07 | 1.19 | 0.95 | 1.05 | 2.69 |  |
| Israel | Infectious diseases |  |  |  |  |  | 0.16 | -0.01 | 0.05 | 0.03 | -0.10 | 0.03 | -0.02 |
|  | Malignant neoplasms |  |  |  |  |  | 0.10 | 0.14 | 0.03 | -0.19 | 0.14 | 0.22 | 0.22 |
|  | Cardiovascular diseases |  |  |  |  |  | **0.55** | 0.82 | 0.72 | 0.44 | 1.37 | 0.73 | 0.70 |
|  | Respiratory diseases |  |  |  |  |  | 0.05 | 0.04 | 0.13 | 0.26 | -0.18 | 0.08 | 0.10 |
|  | Digestive system diseases |  |  |  |  |  | 0.02 | 0.06 | 0.04 | -0.01 | 0.05 | 0.05 | 0.04 |
|  | Perinatal diseases |  |  |  |  |  | 0.18 | 0.20 | 0.04 | 0.08 | 0.05 | 0.06 | 0.01 |
|  | External causes |  |  |  |  |  | 0.45 | -0.10 | 0.15 | 0.11 | -0.11 | 0.26 | 0.22 |
|  | Other diseases |  |  |  |  |  | -0.04 | -0.04 | 0.31 | 0.13 | -0.21 | 0.04 | 0.44 |
|  | Total |  |  |  |  |  | 1.45 | 1.12 | 1.46 | 0.85 | 1.02 | 1.47 | 1.73 |
| Italy | Infectious diseases |  | 0.24 | 0.26 | 0.01 | 0.25 | 0.12 | 0.04 | -0.11 | -0.18 | 0.26 |  |  |
|  | Malignant neoplasms |  | -0.20 | -0.20 | -0.17 | -0.04 | -0.15 | -0.08 | 0.09 | 0.27 | 0.32 |  |  |
|  | Cardiovascular diseases |  | 0.10 | -0.13 | 0.20 | 0.18 | **0.44** | 0.55 | 0.80 | 0.43 | 0.58 |  |  |
|  | Respiratory diseases |  | 0.17 | 0.23 | -0.18 | 0.46 | 0.24 | 0.14 | 0.19 | 0.13 | 0.06 |  |  |
|  | Digestive system diseases |  | 0.16 | 0.09 | 0.18 | 0.04 | 0.11 | 0.14 | 0.21 | 0.15 | 0.13 |  |  |
|  | Perinatal diseases |  | 0.30 | 0.16 | 0.38 | 0.27 | 0.30 | 0.19 | 0.12 | 0.12 | 0.08 |  |  |
|  | External causes |  | -0.02 | 0.02 | -0.03 | 0.16 | 0.06 | 0.20 | 0.00 | 0.17 | 0.11 |  |  |
|  | Other diseases |  | 0.46 | 0.17 | 0.19 | 0.24 | 0.13 | 0.03 | 0.02 | 0.07 | 0.11 |  |  |
|  | Total |  | 1.22 | 0.61 | 0.58 | 1.54 | 1.25 | 1.21 | 1.30 | 1.17 | 1.65 |  |  |
| Japan | Infectious diseases | 2.31 | 0.87 | 0.49 | 0.20 | 0.22 | 0.15 | 0.05 | 0.03 | -0.04 | 0.02 | 0.02 | 0.03 |
|  | Malignant neoplasms | -0.14 | -0.11 | -0.05 | 0.01 | 0.03 | -0.05 | -0.03 | 0.01 | -0.05 | 0.17 | 0.30 | 0.32 |
|  | Cardiovascular diseases | -0.16 | -0.20 | 0.09 | **0.32** | 0.72 | 0.83 | 0.92 | 0.66 | 0.58 | 0.50 | 0.32 | 0.42 |
|  | Respiratory diseases | 0.72 | 0.19 | 0.43 | -0.07 | 0.17 | 0.17 | -0.05 | -0.11 | 0.01 | 0.15 | 0.09 | 0.16 |
|  | Digestive system diseases | 1.05 | 0.47 | 0.33 | 0.25 | 0.11 | 0.13 | 0.14 | 0.11 | 0.08 | 0.07 | 0.05 | 0.04 |
|  | Perinatal diseases | 0.34 | 0.37 | 0.37 | 0.30 | 0.13 | 0.11 | 0.12 | 0.08 | 0.01 | 0.03 | 0.02 | 0.01 |
|  | External causes | -0.19 | -0.03 | 0.32 | 0.10 | 0.38 | 0.23 | 0.03 | 0.22 | -0.01 | -0.07 | 0.12 | 0.06 |
|  | Other diseases | 0.89 | 0.74 | 0.45 | 0.51 | 0.36 | 0.37 | 0.15 | 0.12 | 0.08 | 0.17 | 0.05 | -0.03 |
|  | Total | 4.82 | 2.29 | 2.44 | 1.61 | 2.12 | 1.95 | 1.32 | 1.11 | 0.66 | 1.04 | 0.97 | 1.00 |

| **Country** | **Cause of death** | **1950-55** | **1955-60** | **1960-65** | **1965-70** | **1970-75** | **1975-80** | **1980-85** | **1985-90** | **1990-95** | **1995-2000** | **2000-05** | **2005-10** |
| --- | --- | --- | --- | --- | --- | --- | --- | --- | --- | --- | --- | --- | --- |
| Kazakhstan | Infectious diseases |  |  |  |  |  |  |  | 0.23 | -0.66 | 0.32 | 0.25 | 0.27 |
|  | Malignant neoplasms |  |  |  |  |  |  |  | -0.09 | 0.18 | 0.18 | 0.23 | 0.16 |
|  | Cardiovascular diseases |  |  |  |  |  |  |  | -0.06 | -1.72 | -0.14 | -0.29 | 1.45 |
|  | Respiratory diseases |  |  |  |  |  |  |  | 0.66 | -0.52 | 0.58 | 0.36 | 0.25 |
|  | Digestive system diseases |  |  |  |  |  |  |  | 0.08 | -0.19 | -0.05 | -0.20 | 0.08 |
|  | Perinatal diseases |  |  |  |  |  |  |  | -0.09 | 0.03 | 0.11 | -0.03 | -0.22 |
|  | External causes |  |  |  |  |  |  |  | -0.55 | -0.90 | 0.11 | -0.26 | 1.19 |
|  | Other diseases |  |  |  |  |  |  |  | 0.16 | -0.51 | -0.08 | 0.08 | -0.14 |
|  | Total |  |  |  |  |  |  |  | 0.35 | -4.29 | 1.03 | 0.15 | 3.05 |
| Kuwait | Infectious diseases |  |  |  |  |  | 0.36 | 0.55 |  |  | 0.10 |  |  |
|  | Malignant neoplasms |  |  |  |  |  | 0.31 | 0.00 |  |  | 0.17 |  |  |
|  | Cardiovascular diseases |  |  |  |  |  | -0.21 | 1.00 |  |  | 0.69 |  |  |
|  | Respiratory diseases |  |  |  |  |  | 0.33 | 0.44 |  |  | 0.18 |  |  |
|  | Digestive system diseases |  |  |  |  |  | 0.02 | 0.20 |  |  | 0.03 |  |  |
|  | Perinatal diseases |  |  |  |  |  | 0.07 | -0.02 |  |  | -0.05 |  |  |
|  | External causes |  |  |  |  |  | 0.16 | 0.39 |  |  | 0.09 |  |  |
|  | Other diseases |  |  |  |  |  | 0.70 | 0.22 |  |  | 0.64 |  |  |
|  | Total |  |  |  |  |  | 1.73 | 2.80 |  |  | 1.85 |  |  |
| Kyrgyzstan | Infectious diseases |  |  |  |  |  |  |  | 0.44 | -0.21 | -0.18 | 0.45 |  |
|  | Malignant neoplasms |  |  |  |  |  |  |  | -0.02 | 0.30 | 0.16 | 0.05 |  |
|  | Cardiovascular diseases |  |  |  |  |  |  |  | -0.15 | -1.42 | 0.61 | -0.34 |  |
|  | Respiratory diseases |  |  |  |  |  |  |  | 1.16 | -0.13 | 0.81 | 0.41 |  |
|  | Digestive system diseases |  |  |  |  |  |  |  | -0.01 | -0.26 | 0.02 | -0.26 |  |
|  | Perinatal diseases |  |  |  |  |  |  |  | -0.16 | 0.08 | -0.06 | -0.71 |  |
|  | External causes |  |  |  |  |  |  |  | -1.08 | -0.11 | 0.79 | 0.04 |  |
|  | Other diseases |  |  |  |  |  |  |  | -0.16 | -0.87 | 0.52 | -0.04 |  |
|  | Total |  |  |  |  |  |  |  | 0.00 | -2.62 | 2.68 | -0.40 |  |
| Latvia | Infectious diseases |  |  |  |  |  |  | 0.12 | 0.00 | -0.14 | 0.13 | 0.07 | 0.02 |
|  | Malignant neoplasms |  |  |  |  |  |  | -0.11 | -0.19 | 0.04 | 0.09 | 0.05 | 0.15 |
|  | Cardiovascular diseases |  |  |  |  |  |  | 0.12 | 0.19 | -1.42 | 1.69 | -0.17 | 1.03 |
|  | Respiratory diseases |  |  |  |  |  |  | 0.09 | 0.27 | -0.22 | 0.29 | -0.05 | 0.18 |
|  | Digestive system diseases |  |  |  |  |  |  | 0.05 | 0.04 | -0.16 | 0.04 | -0.05 | 0.04 |
|  | Perinatal diseases |  |  |  |  |  |  | 0.00 | -0.14 | -0.12 | 0.24 | 0.11 | 0.06 |
|  | External causes |  |  |  |  |  |  | 0.93 | -0.64 | -1.31 | 1.31 | 0.78 | 1.19 |
|  | Other diseases |  |  |  |  |  |  | 0.12 | -0.03 | -0.37 | 0.27 | 0.10 | 0.12 |
|  | Total |  |  |  |  |  |  | 1.33 | -0.49 | -3.71 | 4.05 | 0.82 | 2.82 |
| Lithuania | Infectious diseases |  |  |  |  |  |  |  | 0.01 | -0.15 | 0.10 | 0.01 | 0.06 |
|  | Malignant neoplasms |  |  |  |  |  |  |  | -0.18 | -0.13 | 0.13 | 0.09 | 0.08 |
|  | Cardiovascular diseases |  |  |  |  |  |  |  | -0.12 | -0.47 | 0.85 | -0.45 | 0.57 |
|  | Respiratory diseases |  |  |  |  |  |  |  | 0.30 | -0.04 | 0.13 | -0.11 | 0.20 |
|  | Digestive system diseases |  |  |  |  |  |  |  | 0.02 | -0.16 | -0.05 | -0.29 | -0.05 |
|  | Perinatal diseases |  |  |  |  |  |  |  | -0.06 | 0.02 | 0.19 | 0.00 | 0.04 |
|  | External causes |  |  |  |  |  |  |  | -0.62 | -1.28 | 0.86 | 0.26 | 1.02 |
|  | Other diseases |  |  |  |  |  |  |  | 0.10 | -0.44 | 0.64 | -0.17 | 0.16 |
|  | Total |  |  |  |  |  |  |  | -0.53 | -2.64 | 2.86 | -0.67 | 2.08 |
| Mauritius | Infectious diseases |  |  |  | -2.08 | 0.49 | 1.01 | 0.65 | 0.12 | 0.15 | 0.14 | -0.08 |  |
|  | Malignant neoplasms |  |  |  | -0.04 | -0.02 | -0.08 | 0.03 | 0.04 | 0.04 | -0.02 | 0.02 |  |
|  | Cardiovascular diseases |  |  |  | -0.07 | -0.65 | -0.80 | 0.37 | **0.84** | 0.44 | 0.13 | 1.33 |  |
|  | Respiratory diseases |  |  |  | -0.42 | 0.02 | 0.79 | 0.18 | 0.39 | 0.03 | 0.17 | 0.19 |  |
|  | Digestive system diseases |  |  |  | 1.67 | -0.27 | 0.01 | 0.04 | 0.09 | -0.13 | 0.06 | 0.16 |  |
|  | Perinatal diseases |  |  |  | 0.64 | 0.24 | 0.11 | -0.22 | 0.18 | 0.01 | 0.25 | 0.20 |  |
|  | External causes |  |  |  | 0.00 | -0.42 | 0.04 | 0.29 | -0.09 | -0.10 | 0.22 | 0.08 |  |
|  | Other diseases |  |  |  | 1.23 | -0.37 | 1.10 | 1.09 | -0.57 | 0.42 | 0.60 | -0.99 |  |
|  | Total |  |  |  | 0.92 | -0.99 | 2.18 | 2.41 | 1.01 | 0.85 | 1.53 | 0.90 |  |
| Mexico | Infectious diseases |  |  | 0.71 | -1.95 | 1.23 |  |  | 0.29 | 0.57 |  |  |  |
|  | Malignant neoplasms |  |  | -0.09 | 0.02 | -0.06 |  |  | -0.10 | 0.00 |  |  |  |
|  | Cardiovascular diseases |  |  | 0.43 | -0.49 | -0.32 |  |  | -0.02 | -0.01 |  |  |  |
|  | Respiratory diseases |  |  | 0.32 | -0.30 | 0.99 |  |  | 0.15 | 0.14 |  |  |  |
|  | Digestive system diseases |  |  | 0.89 | 1.69 | 0.17 |  |  | 0.13 | 0.02 |  |  |  |
|  | Perinatal diseases |  |  | 0.13 | 0.95 | 0.17 |  |  | -0.15 | 0.10 |  |  |  |
|  | External causes |  |  | 0.11 | -0.03 | -0.58 |  |  | 0.56 | 0.33 |  |  |  |
|  | Other diseases |  |  | -1.05 | 1.17 | 0.99 |  |  | -0.07 | 0.13 |  |  |  |
|  | Total |  |  | 1.47 | 1.05 | 2.58 |  |  | 0.80 | 1.28 |  |  |  |
| Netherlands | Infectious diseases | 0.43 | 0.15 | 0.07 | -0.03 | 0.02 | 0.04 | -0.01 | -0.07 | -0.04 |  | 0.02 | 0.02 |
|  | Malignant neoplasms | -0.08 | -0.19 | -0.19 | -0.13 | -0.11 | -0.01 | 0.01 | 0.17 | 0.17 |  | 0.25 | 0.24 |
|  | Cardiovascular diseases | -0.42 | 0.06 | -0.38 | -0.24 | 0.18 | **0.31** | 0.29 | 0.68 | 0.38 |  | 0.71 | 0.81 |
|  | Respiratory diseases | 0.13 | -0.01 | 0.09 | -0.17 | 0.10 | 0.12 | -0.08 | -0.01 | 0.02 |  | 0.16 | 0.16 |
|  | Digestive system diseases | 0.10 | 0.01 | 0.03 | 0.05 | 0.02 | 0.01 | 0.03 | 0.02 | 0.01 |  | 0.03 | 0.06 |
|  | Perinatal diseases | 0.20 | 0.15 | 0.03 | 0.16 | 0.12 | 0.13 | 0.05 | -0.01 | 0.03 |  | 0.01 | 0.04 |
|  | External causes | -0.06 | -0.09 | -0.09 | -0.09 | 0.25 | 0.22 | 0.13 | 0.13 | 0.05 |  | 0.10 | 0.06 |
|  | Other diseases | 0.35 | 0.34 | 0.09 | 0.19 | 0.04 | 0.22 | 0.04 | -0.09 | 0.10 |  | 0.29 | 0.23 |
|  | Total | 0.65 | 0.43 | -0.35 | -0.25 | 0.61 | 1.03 | 0.47 | 0.82 | 0.74 |  | 1.56 | 1.61 |

| **Country** | **Cause of death** | **1950-55** | **1955-60** | **1960-65** | **1965-70** | **1970-75** | **1975-80** | **1980-85** | **1985-90** | **1990-95** | **1995-2000** | **2000-05** | **2005-10** |
| --- | --- | --- | --- | --- | --- | --- | --- | --- | --- | --- | --- | --- | --- |
| New Zealand | Infectious diseases | 0.46 | 0.25 | 0.10 | -0.03 | 0.05 | 0.04 | 0.01 | -0.04 | 0.02 | 0.03 | 0.03 |  |
|  | Malignant neoplasms | -0.08 | -0.02 | -0.11 | -0.22 | -0.05 | 0.02 | 0.01 | 0.13 | 0.16 | 0.21 | 0.47 |  |
|  | Cardiovascular diseases | 0.29 | -0.17 | -0.42 | 0.08 | **0.44** | 0.50 | 0.78 | 0.96 | 0.71 | 0.96 | 0.89 |  |
|  | Respiratory diseases | -0.11 | -0.18 | -0.08 | -0.06 | 0.21 | 0.13 | 0.08 | 0.33 | 0.13 | 0.32 | 0.19 |  |
|  | Digestive system diseases | 0.04 | 0.07 | 0.08 | 0.15 | -0.08 | 0.06 | 0.03 | 0.04 | 0.05 | 0.06 | 0.02 |  |
|  | Perinatal diseases | 0.28 | 0.00 | 0.17 | 0.15 | 0.10 | 0.22 | 0.09 | -0.03 | 0.07 | 0.00 | 0.00 |  |
|  | External causes | -0.07 | 0.20 | -0.11 | -0.11 | 0.02 | 0.15 | -0.03 | -0.10 | 0.36 | 0.25 | 0.15 |  |
|  | Other diseases | 0.19 | 0.12 | 0.15 | 0.15 | 0.00 | -0.10 | 0.03 | 0.14 | 0.10 | -0.06 | 0.13 |  |
|  | Total | 0.99 | 0.27 | -0.23 | 0.12 | 0.68 | 1.03 | 1.01 | 1.43 | 1.60 | 1.76 | 1.88 |  |
| Norway | Infectious diseases |  | 0.22 | 0.11 | -0.01 | 0.01 | 0.02 | 0.02 | -0.01 | -0.02 | 0.04 | -0.02 | -0.02 |
|  | Malignant neoplasms |  | 0.00 | -0.01 | -0.12 | 0.02 | -0.07 | -0.05 | 0.03 | 0.03 | 0.13 | 0.27 | 0.20 |
|  | Cardiovascular diseases |  | -0.57 | -0.36 | -0.33 | **0.39** | 0.25 | 0.17 | 0.58 | 0.81 | 0.84 | 1.06 | 0.67 |
|  | Respiratory diseases |  | 0.02 | 0.04 | -0.20 | -0.01 | 0.09 | 0.06 | 0.02 | 0.07 | 0.08 | 0.11 | 0.04 |
|  | Digestive system diseases |  | 0.04 | 0.01 | 0.08 | -0.02 | -0.03 | 0.02 | 0.03 | 0.03 | 0.01 | 0.05 | 0.04 |
|  | Perinatal diseases |  | 0.06 | 0.06 | 0.15 | 0.20 | 0.19 | 0.03 | -0.04 | 0.06 | 0.04 | 0.01 | 0.01 |
|  | External causes |  | 0.02 | 0.06 | -0.23 | 0.10 | 0.09 | 0.09 | 0.13 | 0.38 | -0.02 | -0.03 | 0.15 |
|  | Other diseases |  | 0.08 | 0.08 | 0.29 | 0.15 | 0.03 | 0.10 | -0.02 | 0.11 | -0.22 | 0.44 | -0.02 |
|  | Total |  | -0.14 | -0.01 | -0.36 | 0.84 | 0.59 | 0.42 | 0.71 | 1.47 | 0.90 | 1.90 | 1.07 |
| Poland | Infectious diseases |  |  | 0.34 | 0.31 | 0.41 | 0.05 | 0.13 | 0.05 | 0.05 |  | 0.02 | 0.00 |
|  | Malignant neoplasms |  |  | -0.41 | -0.16 | -0.12 | -0.20 | -0.21 | -0.10 | 0.04 |  | 0.13 | 0.34 |
|  | Cardiovascular diseases |  |  | 0.05 | -0.64 | -0.25 | -0.57 | -0.35 | -0.23 | **0.76** |  | 0.76 | 0.49 |
|  | Respiratory diseases |  |  | 0.41 | -0.26 | 0.58 | 0.15 | 0.02 | 0.18 | 0.15 |  | 0.03 | 0.01 |
|  | Digestive system diseases |  |  | 0.19 | 0.25 | 0.04 | -0.01 | 0.06 | 0.02 | -0.02 |  | -0.01 | 0.03 |
|  | Perinatal diseases |  |  | 0.16 | 0.35 | 0.15 | 0.09 | 0.05 | 0.08 | 0.06 |  | 0.06 | 0.06 |
|  | External causes |  |  | -0.07 | -0.30 | -0.11 | -0.13 | 0.12 | -0.19 | 0.24 |  | 0.15 | 0.16 |
|  | Other diseases |  |  | 1.33 | 0.50 | 0.28 | -0.10 | 0.11 | 0.00 | 0.01 |  | 0.15 | 0.19 |
|  | Total |  |  | 2.02 | 0.04 | 0.98 | -0.71 | -0.08 | -0.19 | 1.28 |  | 1.29 | 1.29 |
| Portugal | Infectious diseases |  | 0.49 | 0.53 | 0.18 | 0.38 | 0.29 | 0.21 | -0.01 | -0.30 | 0.01 |  |  |
|  | Malignant neoplasms |  | -0.14 | -0.16 | -0.09 | -0.04 | -0.08 | -0.01 | 0.00 | -0.07 | 0.05 |  |  |
|  | Cardiovascular diseases |  | 0.47 | -0.11 | -0.27 | -0.24 | 0.49 | **0.59** | 0.48 | 0.60 | 0.66 |  |  |
|  | Respiratory diseases |  | -0.08 | 0.11 | -0.02 | 0.70 | 0.68 | 0.32 | 0.17 | 0.08 | -0.02 |  |  |
|  | Digestive system diseases |  | 0.45 | 0.83 | 0.97 | 0.37 | 0.39 | 0.06 | 0.17 | 0.11 | 0.12 |  |  |
|  | Perinatal diseases |  | 0.11 | 0.59 | 0.31 | 0.44 | 0.18 | 0.10 | 0.27 | 0.19 | 0.06 |  |  |
|  | External causes |  | 0.04 | -0.16 | -0.22 | -0.38 | 0.05 | 0.23 | 0.09 | 0.47 | 0.42 |  |  |
|  | Other diseases |  | 0.80 | 0.11 | 0.14 | 0.17 | 0.40 | 0.36 | -0.07 | 0.12 | 0.06 |  |  |
|  | Total |  | 2.14 | 1.75 | 1.01 | 1.39 | 2.40 | 1.85 | 1.10 | 1.19 | 1.36 |  |  |
| Puerto Rico | Infectious diseases |  |  |  |  | 0.31 |  | 0.00 | -0.10 |  |  |  |  |
|  | Malignant neoplasms |  |  |  |  | 0.12 |  | 0.02 | -0.08 |  |  |  |  |
|  | Cardiovascular diseases |  |  |  |  | -0.13 |  | 0.20 | 0.18 |  |  |  |  |
|  | Respiratory diseases |  |  |  |  | 0.12 |  | -0.29 | 0.21 |  |  |  |  |
|  | Digestive system diseases |  |  |  |  | -0.06 |  | 0.19 | 0.09 |  |  |  |  |
|  | Perinatal diseases |  |  |  |  | 0.27 |  | 0.25 | 0.06 |  |  |  |  |
|  | External causes |  |  |  |  | -0.24 |  | 0.18 | -0.12 |  |  |  |  |
|  | Other diseases |  |  |  |  | 0.58 |  | -0.20 | -1.85 |  |  |  |  |
|  | Total |  |  |  |  | 0.98 |  | 0.35 | -1.61 |  |  |  |  |
| Republic of Korea | Infectious diseases |  |  |  |  |  |  |  | 0.24 | 0.07 | 0.05 | 0.10 | 0.04 |
|  | Malignant neoplasms |  |  |  |  |  |  |  | -0.13 | -0.32 | 0.20 | 0.30 | 0.54 |
|  | Cardiovascular diseases |  |  |  |  |  |  |  | **1.17** | 0.44 | 0.81 | 0.62 | 0.59 |
|  | Respiratory diseases |  |  |  |  |  |  |  | 0.15 | -0.08 | -0.06 | 0.20 | 0.05 |
|  | Digestive system diseases |  |  |  |  |  |  |  | 0.34 | 0.11 | 0.37 | 0.36 | 0.19 |
|  | Perinatal diseases |  |  |  |  |  |  |  | -0.01 | -0.01 | -0.15 | 0.02 | 0.03 |
|  | External causes |  |  |  |  |  |  |  | -0.35 | 0.00 | 0.66 | 0.29 | 0.14 |
|  | Other diseases |  |  |  |  |  |  |  | 0.90 | 0.01 | 0.19 | 0.65 | 0.48 |
|  | Total |  |  |  |  |  |  |  | 2.31 | 0.22 | 2.08 | 2.53 | 2.07 |
| Republic of Moldova | Infectious diseases |  |  |  |  |  |  |  | 0.22 | -0.16 | -0.05 | -0.02 | 0.09 |
|  | Malignant neoplasms |  |  |  |  |  |  |  | -0.29 | -0.01 | 0.20 | -0.09 | 0.02 |
|  | Cardiovascular diseases |  |  |  |  |  |  |  | 0.72 | -0.99 | 0.00 | -0.01 | 0.55 |
|  | Respiratory diseases |  |  |  |  |  |  |  | 0.67 | -0.28 | 0.30 | 0.11 | 0.22 |
|  | Digestive system diseases |  |  |  |  |  |  |  | 0.23 | -0.35 | 0.17 | -0.14 | 0.17 |
|  | Perinatal diseases |  |  |  |  |  |  |  | 0.18 | -0.07 | 0.08 | 0.14 | -0.07 |
|  | External causes |  |  |  |  |  |  |  | -0.29 | -0.13 | 0.50 | 0.08 | 0.35 |
|  | Other diseases |  |  |  |  |  |  |  | -0.37 | -0.57 | 0.50 | 0.17 | 0.00 |
|  | Total |  |  |  |  |  |  |  | 1.07 | -2.55 | 1.70 | 0.25 | 1.33 |
| Romania | Infectious diseases |  |  |  |  | 0.60 |  | 0.02 | -0.11 | -0.11 | 0.10 | 0.13 | 0.09 |
|  | Malignant neoplasms |  |  |  |  | -0.03 |  | -0.07 | -0.06 | -0.20 | -0.03 | -0.09 | -0.03 |
|  | Cardiovascular diseases |  |  |  |  | 0.15 |  | -0.11 | 0.06 | -0.93 | **0.80** | 0.52 | 0.70 |
|  | Respiratory diseases |  |  |  |  | 0.69 |  | 0.23 | 0.20 | 0.23 | 0.38 | 0.24 | 0.13 |
|  | Digestive system diseases |  |  |  |  | 0.05 |  | -0.07 | 0.03 | -0.26 | 0.14 | 0.11 | -0.09 |
|  | Perinatal diseases |  |  |  |  | 0.30 |  | 0.12 | -0.01 | -0.08 | 0.01 | 0.01 | 0.19 |
|  | External causes |  |  |  |  | 0.07 |  | 0.08 | -0.20 | -0.01 | 0.46 | 0.23 | 0.17 |
|  | Other diseases |  |  |  |  | 0.06 |  | -0.12 | 0.04 | 0.07 | 0.24 | 0.11 | 0.02 |
|  | Total |  |  |  |  | 1.89 |  | 0.08 | -0.06 | -1.29 | 2.11 | 1.27 | 1.19 |

| **Country** | **Cause of death** | **1950-55** | **1955-60** | **1960-65** | **1965-70** | **1970-75** | **1975-80** | **1980-85** | **1985-90** | **1990-95** | **1995-2000** | **2000-05** | **2005-10** |
| --- | --- | --- | --- | --- | --- | --- | --- | --- | --- | --- | --- | --- | --- |
| Russian Federation | Infectious diseases |  |  |  |  |  |  | 0.10 | 0.15 | -0.20 | -0.08 | 0.02 | 0.06 |
|  | Malignant neoplasms |  |  |  |  |  |  | -0.08 | -0.10 | 0.04 | 0.17 | 0.15 | 0.09 |
|  | Cardiovascular diseases |  |  |  |  |  |  | 0.10 | 0.33 | -1.69 | 0.08 | -0.42 | 1.21 |
|  | Respiratory diseases |  |  |  |  |  |  | 0.35 | 0.46 | -0.29 | 0.13 | 0.07 | 0.21 |
|  | Digestive system diseases |  |  |  |  |  |  | 0.05 | 0.08 | -0.25 | 0.02 | -0.21 | 0.04 |
|  | Perinatal diseases |  |  |  |  |  |  | -0.14 | -0.04 | 0.00 | 0.09 | 0.13 | 0.10 |
|  | External causes |  |  |  |  |  |  | 1.33 | -0.74 | -2.23 | 0.43 | 0.35 | 1.46 |
|  | Other diseases |  |  |  |  |  |  | -0.29 | 0.64 | -0.56 | 0.03 | 0.01 | 0.29 |
|  | Total |  |  |  |  |  |  | 1.42 | 0.79 | -5.18 | 0.86 | 0.10 | 3.47 |
| Singapore | Infectious diseases |  |  |  | 0.46 | 0.25 | 0.37 | 0.18 | 0.15 | 0.07 | 0.11 | 0.04 | 0.06 |
|  | Malignant neoplasms |  |  |  | 0.05 | -0.24 | 0.05 | 0.12 | 0.21 | 0.22 | 0.34 | 0.69 | 0.16 |
|  | Cardiovascular diseases |  |  |  | -0.25 | 0.09 | 0.11 | **0.44** | 0.65 | 0.69 | 0.61 | 0.98 | 0.47 |
|  | Respiratory diseases |  |  |  | -0.16 | 0.04 | 0.18 | 0.26 | 0.64 | 0.15 | 0.62 | -0.03 | 0.16 |
|  | Digestive system diseases |  |  |  | 0.22 | 0.12 | 0.12 | 0.10 | 0.07 | 0.06 | 0.14 | 0.04 | 0.04 |
|  | Perinatal diseases |  |  |  | 0.36 | 0.29 | 0.11 | 0.15 | 0.08 | 0.11 | 0.04 | 0.00 | -0.02 |
|  | External causes |  |  |  | -0.04 | 0.22 | 0.18 | -0.09 | 0.26 | 0.28 | 0.12 | 0.13 | 0.14 |
|  | Other diseases |  |  |  | 1.39 | 0.54 | 0.61 | 0.30 | 0.41 | 0.30 | -0.01 | 0.14 | 0.16 |
|  | Total |  |  |  | 2.03 | 1.31 | 1.74 | 1.47 | 2.47 | 1.89 | 1.97 | 1.98 | 1.18 |
| Slovenia | Infectious diseases |  |  |  |  |  |  |  | -0.01 | 0.06 | 0.00 | 0.00 | 0.05 |
|  | Malignant neoplasms |  |  |  |  |  |  |  | -0.01 | 0.03 | 0.20 | 0.24 | 0.12 |
|  | Cardiovascular diseases |  |  |  |  |  |  |  | **0.79** | 0.63 | 0.53 | 0.63 | 0.89 |
|  | Respiratory diseases |  |  |  |  |  |  |  | -0.06 | 0.05 | 0.09 | 0.18 | 0.32 |
|  | Digestive system diseases |  |  |  |  |  |  |  | 0.17 | 0.09 | 0.02 | 0.24 | 0.07 |
|  | Perinatal diseases |  |  |  |  |  |  |  | 0.28 | 0.13 | 0.01 | 0.00 | 0.05 |
|  | External causes |  |  |  |  |  |  |  | 0.27 | 0.19 | 0.45 | 0.28 | 0.35 |
|  | Other diseases |  |  |  |  |  |  |  | -0.07 | -0.07 | 0.10 | 0.29 | 0.37 |
|  | Total |  |  |  |  |  |  |  | 1.36 | 1.12 | 1.39 | 1.87 | 2.21 |
| Spain | Infectious diseases |  | 0.35 | 0.32 |  |  | 0.15 | 0.10 | -0.22 | -0.33 | 0.45 | 0.04 | 0.09 |
|  | Malignant neoplasms |  | -0.23 | -0.16 |  |  | -0.04 | -0.12 | -0.19 | -0.06 | 0.14 | 0.26 | 0.28 |
|  | Cardiovascular diseases |  | 0.39 | -0.03 |  |  | **0.59** | 0.60 | 0.63 | 0.55 | 0.56 | 0.46 | 0.49 |
|  | Respiratory diseases |  | 0.39 | 0.34 |  |  | 0.47 | 0.12 | 0.01 | 0.11 | -0.03 | 0.15 | 0.18 |
|  | Digestive system diseases |  | 0.39 | 0.16 |  |  | 0.08 | 0.07 | 0.08 | 0.14 | 0.11 | 0.06 | 0.09 |
|  | Perinatal diseases |  | 0.29 | 0.24 |  |  | 0.06 | 0.17 | 0.05 | 0.07 | 0.03 | -0.01 | 0.03 |
|  | External causes |  | -0.04 | -0.03 |  |  | 0.03 | 0.02 | -0.25 | 0.38 | 0.09 | 0.18 | 0.36 |
|  | Other diseases |  | 0.88 | 0.42 |  |  | 0.37 | 0.02 | 0.05 | 0.11 | -0.02 | 0.08 | 0.02 |
|  | Total |  | 2.42 | 1.26 |  |  | 1.71 | 0.97 | 0.17 | 0.96 | 1.35 | 1.24 | 1.53 |
| Sweden | Infectious diseases |  | 0.12 | 0.06 | 0.00 | 0.02 | 0.04 | 0.01 | -0.02 | -0.02 |  | -0.01 | -0.04 |
|  | Malignant neoplasms |  | -0.17 | 0.03 | -0.09 | -0.17 | 0.12 | 0.18 | 0.05 | 0.11 |  | 0.12 | 0.25 |
|  | Cardiovascular diseases |  | 0.09 | -0.02 | -0.02 | 0.02 | 0.00 | **0.56** | 0.83 | 0.72 |  | 0.83 | 0.60 |
|  | Respiratory diseases |  | 0.09 | -0.01 | -0.01 | 0.12 | -0.05 | -0.06 | 0.12 | 0.08 |  | 0.11 | 0.06 |
|  | Digestive system diseases |  | 0.11 | 0.00 | 0.01 | -0.06 | 0.11 | 0.15 | -0.02 | 0.05 |  | 0.00 | 0.04 |
|  | Perinatal diseases |  | 0.01 | 0.20 | 0.13 | 0.20 | 0.12 | 0.05 | 0.01 | 0.05 |  | 0.03 | 0.01 |
|  | External causes |  | 0.09 | -0.11 | -0.02 | 0.02 | 0.20 | 0.19 | 0.13 | 0.31 |  | -0.01 | 0.13 |
|  | Other diseases |  | 0.19 | 0.13 | 0.22 | 0.03 | 0.06 | 0.03 | -0.11 | 0.15 |  | 0.08 | -0.06 |
|  | Total |  | 0.53 | 0.27 | 0.23 | 0.17 | 0.60 | 1.10 | 0.99 | 1.46 |  | 1.15 | 0.99 |
| Switzerland | Infectious diseases |  | 0.26 | 0.13 | 0.02 | 0.05 | 0.06 | 0.01 | -0.17 | -0.04 | 0.24 | 0.06 | 0.01 |
|  | Malignant neoplasms |  | -0.01 | 0.03 | -0.07 | -0.02 | 0.07 | 0.01 | 0.15 | 0.40 | 0.29 | 0.35 | 0.25 |
|  | Cardiovascular diseases |  | **0.30** | 0.04 | 0.45 | 0.21 | 0.26 | 0.62 | 0.54 | 0.69 | 0.63 | 0.72 | 0.47 |
|  | Respiratory diseases |  | 0.10 | 0.09 | -0.23 | 0.20 | 0.14 | 0.08 | -0.05 | 0.15 | 0.05 | 0.16 | 0.09 |
|  | Digestive system diseases |  | 0.09 | 0.01 | 0.08 | 0.13 | 0.09 | 0.10 | 0.09 | 0.00 | 0.02 | 0.04 | 0.04 |
|  | Perinatal diseases |  | 0.19 | 0.21 | 0.25 | 0.21 | 0.19 | 0.07 | 0.01 | 0.01 | -0.03 | 0.01 | -0.02 |
|  | External causes |  | 0.01 | 0.13 | -0.07 | 0.31 | 0.06 | 0.19 | -0.01 | 0.55 | 0.27 | 0.24 | 0.14 |
|  | Other diseases |  | 0.15 | 0.04 | 0.14 | 0.36 | 0.04 | 0.09 | 0.00 | -0.33 | 0.07 | 0.14 | 0.19 |
|  | Total |  | 1.10 | 0.68 | 0.58 | 1.45 | 0.92 | 1.16 | 0.54 | 1.44 | 1.55 | 1.71 | 1.16 |
| TFYR Macedonia | Infectious diseases |  |  |  |  |  |  |  |  |  | 0.13 | 0.07 | 0.05 |
|  | Malignant neoplasms |  |  |  |  |  |  |  |  |  | -0.03 | 0.01 | -0.08 |
|  | Cardiovascular diseases |  |  |  |  |  |  |  |  |  | 0.20 | 0.18 | 0.47 |
|  | Respiratory diseases |  |  |  |  |  |  |  |  |  | 0.19 | -0.01 | 0.06 |
|  | Digestive system diseases |  |  |  |  |  |  |  |  |  | 0.05 | 0.02 | 0.02 |
|  | Perinatal diseases |  |  |  |  |  |  |  |  |  | 0.13 | 0.04 | 0.07 |
|  | External causes |  |  |  |  |  |  |  |  |  | -0.20 | 0.31 | 0.14 |
|  | Other diseases |  |  |  |  |  |  |  |  |  | 0.27 | 0.25 | 0.22 |
|  | Total |  |  |  |  |  |  |  |  |  | 0.72 | 0.87 | 0.95 |
| Tajikistan | Infectious diseases |  |  |  |  |  |  |  | -0.18 | 0.24 | 1.07 |  |  |
|  | Malignant neoplasms |  |  |  |  |  |  |  | 0.02 | 0.60 | 0.09 |  |  |
|  | Cardiovascular diseases |  |  |  |  |  |  |  | -0.67 | -0.98 | 0.71 |  |  |
|  | Respiratory diseases |  |  |  |  |  |  |  | 0.47 | -0.07 | 1.84 |  |  |
|  | Digestive system diseases |  |  |  |  |  |  |  | 0.48 | -0.15 | 0.14 |  |  |
|  | Perinatal diseases |  |  |  |  |  |  |  | -0.07 | 0.08 | 0.26 |  |  |
|  | External causes |  |  |  |  |  |  |  | -0.17 | -0.61 | 1.11 |  |  |
|  | Other diseases |  |  |  |  |  |  |  | -0.14 | -0.66 | 0.55 |  |  |
|  | Total |  |  |  |  |  |  |  | -0.26 | -1.54 | 5.77 |  |  |

| **Country** | **Cause of death** | **1950-55** | **1955-60** | **1960-65** | **1965-70** | **1970-75** | **1975-80** | **1980-85** | **1985-90** | **1990-95** | **1995-2000** | **2000-05** | **2005-10** |
| --- | --- | --- | --- | --- | --- | --- | --- | --- | --- | --- | --- | --- | --- |
| Trinidad and Tobago | Infectious diseases |  |  |  |  | 0.37 | 1.05 | 0.47 | 0.00 |  |  |  |  |
|  | Malignant neoplasms |  |  |  |  | 0.10 | -0.03 | -0.14 | -0.15 |  |  |  |  |
|  | Cardiovascular diseases |  |  |  |  | **0.40** | 0.42 | 0.65 | 0.42 |  |  |  |  |
|  | Respiratory diseases |  |  |  |  | 0.32 | 0.33 | 0.19 | 0.11 |  |  |  |  |
|  | Digestive system diseases |  |  |  |  | 0.06 | 0.02 | 0.07 | 0.11 |  |  |  |  |
|  | Perinatal diseases |  |  |  |  | 0.17 | 0.15 | 0.08 | 0.01 |  |  |  |  |
|  | External causes |  |  |  |  | 0.06 | 0.19 | -0.06 | 0.24 |  |  |  |  |
|  | Other diseases |  |  |  |  | 0.27 | 0.72 | -0.48 | -0.73 |  |  |  |  |
|  | Total |  |  |  |  | 1.75 | 2.85 | 0.76 | 0.01 |  |  |  |  |
| Turkmenistan | Infectious diseases |  |  |  |  |  |  |  | 0.18 | -0.25 |  |  |  |
|  | Malignant neoplasms |  |  |  |  |  |  |  | 0.21 | 0.33 |  |  |  |
|  | Cardiovascular diseases |  |  |  |  |  |  |  | -0.48 | -1.03 |  |  |  |
|  | Respiratory diseases |  |  |  |  |  |  |  | 1.72 | -0.18 |  |  |  |
|  | Digestive system diseases |  |  |  |  |  |  |  | -0.07 | -0.03 |  |  |  |
|  | Perinatal diseases |  |  |  |  |  |  |  | -0.17 | 0.15 |  |  |  |
|  | External causes |  |  |  |  |  |  |  | -0.24 | 0.44 |  |  |  |
|  | Other diseases |  |  |  |  |  |  |  | 0.04 | -0.11 |  |  |  |
|  | Total |  |  |  |  |  |  |  | 1.18 | -0.68 |  |  |  |
| Ukraine | Infectious diseases |  |  |  |  |  |  |  | 0.04 | -0.20 | -0.17 | -0.16 |  |
|  | Malignant neoplasms |  |  |  |  |  |  |  | -0.24 | 0.04 | 0.17 | 0.14 |  |
|  | Cardiovascular diseases |  |  |  |  |  |  |  | 0.31 | -1.53 | 0.06 | -0.52 |  |
|  | Respiratory diseases |  |  |  |  |  |  |  | 0.18 | -0.25 | 0.17 | 0.15 |  |
|  | Digestive system diseases |  |  |  |  |  |  |  | 0.03 | -0.24 | -0.03 | -0.32 |  |
|  | Perinatal diseases |  |  |  |  |  |  |  | 0.02 | 0.00 | 0.02 | 0.05 |  |
|  | External causes |  |  |  |  |  |  |  | -0.76 | -1.09 | 0.32 | 0.19 |  |
|  | Other diseases |  |  |  |  |  |  |  | -0.21 | -0.36 | 0.22 | -0.14 |  |
|  | Total |  |  |  |  |  |  |  | -0.63 | -3.63 | 0.75 | -0.60 |  |
| United Kingdom | Infectious diseases | 0.60 | 0.22 | 0.08 | -0.01 | 0.03 | 0.07 | 0.01 | -0.02 | -0.05 |  |  | 0.02 |
|  | Malignant neoplasms | -0.09 | -0.07 | -0.04 | -0.02 | 0.04 | 0.09 | 0.05 | 0.11 | 0.25 |  |  | 0.24 |
|  | Cardiovascular diseases | 0.06 | 0.04 | -0.06 | 0.03 | 0.16 | **0.39** | 0.66 | 0.85 | 0.76 |  |  | 0.82 |
|  | Respiratory diseases | 0.40 | -0.09 | 0.08 | -0.11 | 0.27 | 0.29 | 0.44 | 0.15 | -0.10 |  |  | 0.16 |
|  | Digestive system diseases | 0.15 | 0.09 | 0.05 | 0.09 | 0.00 | 0.02 | 0.03 | -0.01 | -0.03 |  |  | 0.03 |
|  | Perinatal diseases | 0.16 | 0.12 | 0.18 | 0.15 | 0.11 | 0.17 | 0.10 | 0.02 | 0.02 |  |  | 0.04 |
|  | External causes | 0.01 | -0.05 | -0.03 | 0.11 | 0.03 | 0.05 | 0.11 | 0.01 | 0.13 |  |  | 0.08 |
|  | Other diseases | 0.23 | 0.20 | 0.09 | 0.13 | -0.06 | 0.10 | -0.10 | 0.09 | 0.25 |  |  | 0.03 |
|  | Total | 1.53 | 0.46 | 0.35 | 0.36 | 0.59 | 1.18 | 1.31 | 1.21 | 1.23 |  |  | 1.42 |
| United States of America | Infectious diseases | 0.38 | 0.12 | 0.05 | 0.02 | 0.02 | 0.04 | -0.07 | -0.39 | -0.12 | 0.37 | 0.03 |  |
|  | Malignant neoplasms | -0.09 | -0.06 | -0.08 | -0.08 | -0.02 | -0.02 | 0.01 | 0.01 | 0.15 | 0.27 | 0.28 |  |
|  | Cardiovascular diseases | 0.32 | -0.02 | 0.09 | **0.33** | 0.81 | 0.91 | 0.69 | 0.82 | 0.44 | 0.67 | 0.69 |  |
|  | Respiratory diseases | 0.05 | -0.14 | -0.06 | 0.00 | 0.17 | 0.11 | -0.05 | 0.01 | 0.05 | 0.09 | 0.10 |  |
|  | Digestive system diseases | 0.04 | 0.00 | 0.00 | 0.02 | 0.06 | 0.09 | 0.08 | 0.05 | 0.04 | 0.02 | 0.03 |  |
|  | Perinatal diseases | 0.10 | 0.00 | 0.07 | 0.24 | 0.24 | 0.17 | 0.10 | 0.06 | 0.07 | 0.00 | 0.00 |  |
|  | External causes | 0.11 | 0.11 | -0.20 | -0.22 | 0.23 | 0.10 | 0.34 | 0.04 | 0.13 | 0.20 | -0.05 |  |
|  | Other diseases | 0.22 | 0.06 | 0.00 | 0.08 | 0.04 | 0.10 | -0.05 | 0.06 | 0.01 | -0.09 | -0.04 |  |
|  | Total | 1.12 | 0.07 | -0.13 | 0.38 | 1.57 | 1.50 | 1.06 | 0.65 | 0.78 | 1.54 | 1.03 |  |
| Uruguay | Infectious diseases |  | 0.12 |  | -0.33 | 0.05 |  | 0.12 | 0.20 |  |  |  |  |
|  | Malignant neoplasms |  | -0.18 |  | -0.12 | 0.09 |  | -0.06 | -0.02 |  |  |  |  |
|  | Cardiovascular diseases |  | -0.54 |  | -0.38 | 0.17 |  | **0.36** | 0.25 |  |  |  |  |
|  | Respiratory diseases |  | -0.14 |  | -0.30 | 0.23 |  | 0.01 | -0.05 |  |  |  |  |
|  | Digestive system diseases |  | 0.08 |  | 0.32 | 0.04 |  | 0.04 | -0.04 |  |  |  |  |
|  | Perinatal diseases |  | -0.43 |  | 0.78 | -0.23 |  | 0.36 | 0.22 |  |  |  |  |
|  | External causes |  | 0.05 |  | -0.10 | 0.09 |  | 0.28 | -0.34 |  |  |  |  |
|  | Other diseases |  | -0.05 |  | -0.45 | 0.19 |  | 0.32 | 0.31 |  |  |  |  |
|  | Total |  | -1.08 |  | -0.58 | 0.61 |  | 1.44 | 0.53 |  |  |  |  |
| Uzbekistan | Infectious diseases |  |  |  |  |  |  |  | 0.36 | 0.11 | 0.26 | 0.21 |  |
|  | Malignant neoplasms |  |  |  |  |  |  |  | 0.12 | 0.32 | 0.21 | 0.12 |  |
|  | Cardiovascular diseases |  |  |  |  |  |  |  | -0.38 | -1.27 | 0.42 | 0.00 |  |
|  | Respiratory diseases |  |  |  |  |  |  |  | 0.57 | 0.15 | 0.74 | 0.59 |  |
|  | Digestive system diseases |  |  |  |  |  |  |  | -0.05 | -0.23 | 0.10 | -0.02 |  |
|  | Perinatal diseases |  |  |  |  |  |  |  | -0.03 | 0.23 | -0.01 | 0.03 |  |
|  | External causes |  |  |  |  |  |  |  | -0.30 | 0.55 | 0.11 | 0.19 |  |
|  | Other diseases |  |  |  |  |  |  |  | 0.17 | -0.51 | 0.37 | 0.07 |  |
|  | Total |  |  |  |  |  |  |  | 0.45 | -0.65 | 2.21 | 1.18 |  |
| Venezuela | Infectious diseases |  | 0.46 | 0.10 | -0.78 | 0.41 | 0.52 |  | -0.12 |  |  |  |  |
|  | Malignant neoplasms |  | -0.11 | -0.13 | -0.04 | 0.04 | 0.20 |  | -0.13 |  |  |  |  |
|  | Cardiovascular diseases |  | -0.05 | -0.15 | -0.29 | -0.12 | 0.29 |  | -0.61 |  |  |  |  |
|  | Respiratory diseases |  | 0.08 | 0.05 | -0.38 | 0.05 | 0.41 |  | -0.11 |  |  |  |  |
|  | Digestive system diseases |  | 0.52 | 0.21 | 0.70 | 0.06 | -0.07 |  | -0.10 |  |  |  |  |
|  | Perinatal diseases |  | 0.01 | -0.09 | 0.32 | -0.05 | 0.01 |  | -0.01 |  |  |  |  |
|  | External causes |  | -0.30 | -0.22 | -0.11 | -0.38 | -0.03 |  | 0.01 |  |  |  |  |
|  | Other diseases |  | 2.96 | 0.85 | 0.38 | 0.77 | 1.12 |  | 1.15 |  |  |  |  |
|  | Total |  | 3.58 | 0.62 | -0.19 | 0.78 | 2.44 |  | 0.07 |  |  |  |  |

| **Country** | **Cause of death** | **1950-55** | **1955-60** | **1960-65** | **1965-70** | **1970-75** | **1975-80** | **1980-85** | **1985-90** | **1990-95** | **1995-2000** | **2000-05** | **2005-10** |
| --- | --- | --- | --- | --- | --- | --- | --- | --- | --- | --- | --- | --- | --- |
| Yugoslavia, Former | Infectious diseases |  |  |  | 0.05 | 0.41 | 0.17 | 0.08 | 0.07 |  |  |  |  |
|  | Malignant neoplasms |  |  |  | -0.17 | -0.18 | -0.21 | -0.08 | -0.14 |  |  |  |  |
|  | Cardiovascular diseases |  |  |  | -0.39 | -0.40 | -0.50 | -0.15 | 0.31 |  |  |  |  |
|  | Respiratory diseases |  |  |  | -0.06 | 0.30 | 0.13 | 0.12 | 0.29 |  |  |  |  |
|  | Digestive system diseases |  |  |  | 0.35 | 0.04 | -0.17 | 0.08 | 0.09 |  |  |  |  |
|  | Perinatal diseases |  |  |  | 1.40 | 0.01 | 0.00 | 0.08 | 0.18 |  |  |  |  |
|  | External causes |  |  |  | -0.39 | 0.20 | 0.00 | 0.24 | 0.07 |  |  |  |  |
|  | Other diseases |  |  |  | -0.62 | 1.99 | 1.01 | -0.09 | 0.35 |  |  |  |  |
|  | Total |  |  |  | 0.16 | 2.36 | 0.43 | 0.29 | 1.22 |  |  |  |  |
| Croatia | Infectious diseases |  |  |  |  |  |  |  | 0.03 | 0.05 | 0.00 | 0.08 | 0.01 |
|  | Malignant neoplasms |  |  |  |  |  |  |  | -0.13 | 0.00 | -0.28 | 0.47 | 0.15 |
|  | Cardiovascular diseases |  |  |  |  |  |  |  | 0.34 | 0.26 | -0.20 | 1.28 | 0.71 |
|  | Respiratory diseases |  |  |  |  |  |  |  | 0.24 | -0.07 | -0.01 | 0.00 | 0.22 |
|  | Digestive system diseases |  |  |  |  |  |  |  | 0.01 | 0.10 | -0.06 | 0.22 | 0.05 |
|  | Perinatal diseases |  |  |  |  |  |  |  | 0.27 | 0.07 | 0.07 | 0.11 | 0.00 |
|  | External causes |  |  |  |  |  |  |  | -0.97 | 0.98 | 0.49 | 0.24 | 0.22 |
|  | Other diseases |  |  |  |  |  |  |  | 0.14 | -0.11 | 0.53 | 0.18 | -0.02 |
|  | Total |  |  |  |  |  |  |  | -0.05 | 1.27 | 0.54 | 2.58 | 1.34 |
| Serbia | Infectious diseases |  |  |  |  |  |  |  |  |  |  | 0.03 | 0.03 |
|  | Malignant neoplasms |  |  |  |  |  |  |  |  |  |  | -0.11 | -0.04 |
|  | Cardiovascular diseases |  |  |  |  |  |  |  |  |  |  | 0.43 | 0.87 |
|  | Respiratory diseases |  |  |  |  |  |  |  |  |  |  | 0.06 | -0.02 |
|  | Digestive system diseases |  |  |  |  |  |  |  |  |  |  | -0.01 | 0.03 |
|  | Perinatal diseases |  |  |  |  |  |  |  |  |  |  | 0.14 | 0.06 |
|  | External causes |  |  |  |  |  |  |  |  |  |  | 0.24 | 0.14 |
|  | Other diseases |  |  |  |  |  |  |  |  |  |  | 0.31 | 0.30 |
|  | Total |  |  |  |  |  |  |  |  |  |  | 1.09 | 1.40 |

* The most positive contributing cause of death as well as age group of at least 0.10 life years was marked with green for each year. The most decreasing life expectancy of at least -0.10 life-years was marked red, respectively. ‘Other diseases’ were not considered. For each country the first of at least two following years with cardiovascular disease being the most positively contributing cause of death to the improvement in live expectancy was printed with bold letters as an indicator for the epidemiologic transition. Correspondingly, the same procedure was performed for the age group 65 years and older.

**Table S3:** Relative change in life expectancy [years] by five-year interval in women according to age of death*

| **Country** | **Age groups** | **1950-55** | **1955-60** | **1960-65** | **1965-70** | **1970-75** | **1975-80** | **1980-85** | **1985-90** | **1990-95** | **1995-2000** | **2000-05** | **2005-10** |
| --- | --- | --- | --- | --- | --- | --- | --- | --- | --- | --- | --- | --- | --- |
| Argentina | 0-4 |  |  |  |  |  |  | 1.60 | 0.14 | 0.31 |  |  |  |
|  | 5-14 |  |  |  |  |  |  | 0.06 | 0.03 | 0.02 |  |  |  |
|  | 15-39 |  |  |  |  |  |  | 0.25 | 0.11 | 0.07 |  |  |  |
|  | 40-64 |  |  |  |  |  |  | 0.21 | 0.23 | 0.21 |  |  |  |
|  | 65+ |  |  |  |  |  |  | 0.42 | **0.58** | 0.34 |  |  |  |
| Armenia | 0-4 |  |  |  |  |  |  |  | 0.82 | 0.32 | 0.12 |  |  |
|  | 5-14 |  |  |  |  |  |  |  | 0.12 | 0.06 | 0.02 |  |  |
|  | 15-39 |  |  |  |  |  |  |  | -0.09 | 0.22 | 0.14 |  |  |
|  | 40-64 |  |  |  |  |  |  |  | -0.19 | 0.20 | 0.38 |  |  |
|  | 65+ |  |  |  |  |  |  |  | -0.45 | -0.05 | 0.80 |  |  |
| Australia | 0-4 | 0.24 | 0.19 | 0.18 | 0.08 | 0.24 | 0.30 | 0.13 | 0.12 | 0.16 | 0.05 |  |  |
|  | 5-14 | 0.09 | 0.04 | 0.02 | 0.01 | 0.02 | 0.04 | 0.02 | 0.02 | 0.00 | 0.03 |  |  |
|  | 15-39 | 0.29 | 0.10 | -0.05 | 0.05 | 0.15 | 0.12 | 0.04 | 0.06 | 0.07 | 0.00 |  |  |
|  | 40-64 | 0.41 | 0.28 | -0.10 | 0.08 | 0.33 | 0.67 | 0.29 | 0.34 | 0.28 | 0.31 |  |  |
|  | 65+ | 0.30 | **0.45** | 0.00 | 0.20 | 0.65 | 1.19 | 0.34 | 0.58 | 0.60 | 1.15 |  |  |
| Austria | 0-4 |  | 0.49 | 0.59 | 0.27 | 0.36 | 0.47 | 0.25 | 0.20 | 0.18 | 0.10 | 0.04 | 0.04 |
|  | 5-14 |  | 0.06 | 0.02 | 0.02 | 0.03 | 0.04 | 0.03 | 0.03 | 0.00 | 0.00 | 0.01 | 0.01 |
|  | 15-39 |  | 0.20 | 0.16 | 0.01 | 0.09 | 0.03 | 0.13 | 0.11 | 0.05 | 0.08 | 0.07 | 0.06 |
|  | 40-64 |  | 0.31 | 0.17 | 0.01 | 0.31 | 0.29 | 0.22 | 0.35 | 0.18 | 0.25 | 0.27 | 0.11 |
|  | 65+ |  | 0.45 | 0.16 | -0.13 | **0.59** | 0.55 | 0.66 | 0.82 | 0.67 | 0.76 | 0.86 | 0.56 |
| Azerbaijan | 0-4 |  |  |  |  |  |  |  | 0.39 | 0.43 | 1.05 |  |  |
|  | 5-14 |  |  |  |  |  |  |  | 0.03 | -0.06 | 0.04 |  |  |
|  | 15-39 |  |  |  |  |  |  |  | 0.03 | -0.28 | 0.32 |  |  |
|  | 40-64 |  |  |  |  |  |  |  | 0.14 | -0.39 | 0.41 |  |  |
|  | 65+ |  |  |  |  |  |  |  | 0.65 | -0.75 | -0.50 |  |  |
| Belarus | 0-4 |  |  |  |  |  |  |  | 0.16 | -0.09 |  |  |  |
|  | 5-14 |  |  |  |  |  |  |  | 0.04 | 0.03 |  |  |  |
|  | 15-39 |  |  |  |  |  |  |  | -0.06 | -0.12 |  |  |  |
|  | 40-64 |  |  |  |  |  |  |  | -0.04 | -0.65 |  |  |  |
|  | 65+ |  |  |  |  |  |  |  | 0.06 | -0.60 |  |  |  |
| Belgium | 0-4 |  | 0.75 | 0.33 | 0.31 | 0.32 | 0.25 | 0.22 | 0.14 | 0.16 |  |  |  |
|  | 5-14 |  | 0.05 | 0.01 | 0.01 | 0.05 | 0.00 | 0.03 | 0.02 | 0.02 |  |  |  |
|  | 15-39 |  | 0.13 | 0.03 | 0.04 | 0.04 | 0.01 | 0.11 | 0.05 | 0.05 |  |  |  |
|  | 40-64 |  | 0.34 | 0.03 | 0.06 | 0.24 | 0.29 | 0.24 | 0.35 | 0.13 |  |  |  |
|  | 65+ |  | 0.42 | 0.15 | 0.02 | **0.49** | 0.84 | 0.64 | 0.86 | 0.60 |  |  |  |
| Bulgaria | 0-4 |  |  |  | 0.45 | 0.25 | 0.28 | 0.24 | 0.07 | -0.02 | 0.15 | 0.26 | 0.17 |
|  | 5-14 |  |  |  | 0.02 | 0.01 | 0.01 | 0.01 | 0.00 | 0.01 | 0.03 | -0.01 | 0.04 |
|  | 15-39 |  |  |  | 0.03 | 0.10 | -0.01 | 0.00 | 0.04 | -0.06 | 0.06 | 0.10 | 0.04 |
|  | 40-64 |  |  |  | -0.07 | 0.09 | 0.02 | 0.05 | 0.01 | -0.07 | 0.06 | 0.14 | 0.23 |
|  | 65+ |  |  |  | -0.54 | 0.01 | 0.13 | 0.04 | 0.28 | 0.06 | 0.15 | **0.64** | 0.76 |
| Canada | 0-4 | 0.62 | 0.36 | 0.27 | 0.36 | 0.36 | 0.30 | 0.18 | 0.08 | 0.11 | 0.09 | -0.02 |  |
|  | 5-14 | 0.10 | 0.04 | 0.02 | 0.01 | 0.03 | 0.04 | 0.04 | 0.01 | 0.02 | 0.02 | 0.02 |  |
|  | 15-39 | 0.41 | 0.12 | 0.05 | 0.00 | 0.04 | 0.10 | 0.16 | 0.01 | 0.08 | 0.03 | 0.04 |  |
|  | 40-64 | 0.52 | 0.31 | 0.17 | 0.16 | 0.16 | 0.31 | 0.31 | 0.24 | 0.17 | 0.24 | 0.12 |  |
|  | 65+ | 0.53 | 0.19 | **0.64** | 0.62 | 0.53 | 0.79 | 0.29 | 0.37 | 0.30 | 0.37 | 0.48 |  |
| Chile | 0-4 |  | -0.04 | 1.63 | 1.92 | 1.55 | 1.90 | 0.93 | 0.30 |  |  |  |  |
|  | 5-14 |  | 0.01 | 0.14 | 0.12 | 0.05 | 0.08 | 0.09 | 0.04 |  |  |  |  |
|  | 15-39 |  | 0.32 | 0.43 | 0.63 | 0.42 | 0.41 | 0.33 | 0.13 |  |  |  |  |
|  | 40-64 |  | 0.23 | 0.43 | 0.21 | 0.90 | 0.49 | 0.56 | 0.29 |  |  |  |  |
|  | 65+ |  | 0.07 | -0.12 | 0.20 | 0.92 | 0.36 | 0.17 | 0.85 |  |  |  |  |
| China,select rural areas | 0-4 |  |  |  |  |  |  |  |  | 0.39 | 0.54 |  |  |
|  | 5-14 |  |  |  |  |  |  |  |  | 0.01 | 0.06 |  |  |
|  | 15-39 |  |  |  |  |  |  |  |  | 0.21 | 0.19 |  |  |
|  | 40-64 |  |  |  |  |  |  |  |  | -0.07 | 0.21 |  |  |
|  | 65+ |  |  |  |  |  |  |  |  | 0.63 | 0.04 |  |  |
| China,select urban and rural areas | 0-4 |  |  |  |  |  |  |  |  | 0.30 | 0.43 |  |  |
|  | 5-14 |  |  |  |  |  |  |  |  | 0.02 | 0.05 |  |  |
|  | 15-39 |  |  |  |  |  |  |  |  | 0.19 | 0.12 |  |  |
|  | 40-64 |  |  |  |  |  |  |  |  | 0.13 | 0.25 |  |  |
|  | 65+ |  |  |  |  |  |  |  |  | 0.53 | 0.33 |  |  |
| China,select urban areas | 0-4 |  |  |  |  |  |  |  |  | 0.26 | 0.32 |  |  |
|  | 5-14 |  |  |  |  |  |  |  |  | 0.03 | 0.04 |  |  |
|  | 15-39 |  |  |  |  |  |  |  |  | 0.17 | 0.07 |  |  |
|  | 40-64 |  |  |  |  |  |  |  |  | 0.25 | 0.28 |  |  |
|  | 65+ |  |  |  |  |  |  |  |  | **0.45** | 0.53 |  |  |

| **Country** | **Age groups** | **1950-55** | **1955-60** | **1960-65** | **1965-70** | **1970-75** | **1975-80** | **1980-85** | **1985-90** | **1990-95** | **1995-2000** | **2000-05** | **2005-10** |
| --- | --- | --- | --- | --- | --- | --- | --- | --- | --- | --- | --- | --- | --- |
| Costa Rica | 0-4 |  |  |  | 1.18 | 2.38 | 1.34 | 0.17 | 0.27 | 0.11 |  |  |  |
|  | 5-14 |  |  |  | 0.05 | 0.19 | 0.05 | 0.05 | 0.02 | 0.02 |  |  |  |
|  | 15-39 |  |  |  | 0.21 | 0.28 | 0.32 | 0.19 | 0.06 | 0.01 |  |  |  |
|  | 40-64 |  |  |  | 0.25 | 0.76 | 0.56 | 0.12 | 0.24 | 0.02 |  |  |  |
|  | 65+ |  |  |  | -0.12 | 1.01 | -0.08 | -0.74 | 1.12 | -0.08 |  |  |  |
| Cuba | 0-4 |  |  |  |  | 1.03 | 0.54 | 0.36 | 0.33 | 0.09 |  |  |  |
|  | 5-14 |  |  |  |  | 0.04 | -0.05 | 0.03 | 0.04 | 0.02 |  |  |  |
|  | 15-39 |  |  |  |  | 0.11 | -0.11 | 0.05 | 0.19 | 0.12 |  |  |  |
|  | 40-64 |  |  |  |  | 0.19 | 0.01 | -0.04 | 0.05 | 0.07 |  |  |  |
|  | 65+ |  |  |  |  | 0.32 | 0.43 | 0.14 | 0.41 | -0.31 |  |  |  |
| Czechoslovakia, Former | 0-4 |  | 0.79 | 0.10 | 0.11 | 0.19 | 0.22 | 0.23 | 0.19 |  |  |  |  |
|  | 5-14 |  | 0.06 | 0.00 | 0.00 | 0.02 | 0.02 | 0.02 | 0.01 |  |  |  |  |
|  | 15-39 |  | 0.30 | 0.10 | -0.02 | 0.11 | 0.04 | 0.04 | 0.00 |  |  |  |  |
|  | 40-64 |  | 0.43 | 0.16 | -0.19 | 0.19 | -0.02 | 0.04 | 0.15 |  |  |  |  |
|  | 65+ |  | 0.55 | 0.22 | -0.18 | 0.17 | 0.05 | 0.10 | 0.44 |  |  |  |  |
| Czech Republic | 0-4 |  |  |  |  |  |  |  |  | 0.16 | 0.25 | 0.08 | 0.05 |
|  | 5-14 |  |  |  |  |  |  |  |  | -0.01 | 0.03 | 0.02 | 0.01 |
|  | 15-39 |  |  |  |  |  |  |  |  | 0.06 | 0.10 | 0.06 | 0.04 |
|  | 40-64 |  |  |  |  |  |  |  |  | 0.39 | 0.33 | 0.23 | 0.29 |
|  | 65+ |  |  |  |  |  |  |  |  | **0.77** | 0.81 | 0.65 | 0.78 |
| Slovakia | 0-4 |  |  |  |  |  |  |  |  |  | 0.23 | 0.02 | 0.13 |
|  | 5-14 |  |  |  |  |  |  |  |  |  | 0.01 | 0.02 | 0.00 |
|  | 15-39 |  |  |  |  |  |  |  |  |  | 0.05 | 0.07 | 0.04 |
|  | 40-64 |  |  |  |  |  |  |  |  |  | 0.23 | 0.20 | 0.17 |
|  | 65+ |  |  |  |  |  |  |  |  |  | **0.35** | 0.43 | 0.56 |
| Denmark | 0-4 |  | 0.23 | 0.27 | 0.33 | 0.27 | 0.10 | 0.04 | 0.05 | 0.15 | 0.06 | 0.07 |  |
|  | 5-14 |  | -0.02 | 0.00 | -0.02 | 0.04 | 0.04 | 0.02 | 0.00 | 0.01 | 0.02 | 0.03 |  |
|  | 15-39 |  | 0.07 | 0.06 | 0.00 | 0.15 | -0.08 | 0.07 | 0.08 | 0.02 | 0.10 | 0.11 |  |
|  | 40-64 |  | 0.26 | 0.11 | -0.09 | 0.06 | -0.01 | -0.04 | 0.09 | 0.18 | 0.41 | 0.42 |  |
|  | 65+ |  | 0.20 | 0.22 | 0.99 | 0.44 | 0.42 | 0.19 | 0.04 | -0.21 | **0.57** | 0.65 |  |
| Estonia | 0-4 |  |  |  |  |  |  |  | 0.10 | 0.06 | 0.27 | 0.28 | 0.21 |
|  | 5-14 |  |  |  |  |  |  |  | 0.03 | 0.00 | 0.03 | 0.06 | 0.01 |
|  | 15-39 |  |  |  |  |  |  |  | -0.09 | -0.31 | 0.38 | 0.24 | 0.14 |
|  | 40-64 |  |  |  |  |  |  |  | -0.03 | -0.54 | 0.47 | 0.59 | 0.91 |
|  | 65+ |  |  |  |  |  |  |  | **0.31** | 0.11 | 0.83 | 0.78 | 1.33 |
| Finland | 0-4 |  | 0.51 | 0.41 | 0.20 | 0.32 | 0.19 | 0.13 | -0.02 | 0.17 | 0.09 | 0.02 | 0.03 |
|  | 5-14 |  | 0.07 | 0.00 | 0.02 | 0.04 | 0.05 | 0.02 | 0.01 | 0.00 | 0.01 | 0.00 | 0.02 |
|  | 15-39 |  | 0.33 | 0.11 | 0.09 | 0.09 | 0.15 | 0.03 | -0.05 | 0.11 | 0.00 | 0.06 | 0.04 |
|  | 40-64 |  | 0.30 | 0.20 | 0.23 | 0.43 | 0.46 | 0.23 | 0.07 | 0.26 | 0.08 | 0.05 | 0.22 |
|  | 65+ |  | 0.27 | -0.04 | **0.55** | 1.21 | 0.94 | 0.47 | 0.34 | 0.76 | 0.85 | 1.15 | 0.56 |
| France | 0-4 |  | 0.72 | 0.48 | 0.24 | 0.22 | 0.24 | 0.12 | 0.11 | 0.17 | 0.06 | 0.06 |  |
|  | 5-14 |  | 0.02 | 0.02 | -0.03 | 0.03 | 0.01 | 0.04 | 0.02 | 0.02 | 0.01 | 0.02 |  |
|  | 15-39 |  | 0.24 | 0.07 | 0.03 | 0.09 | 0.05 | 0.08 | 0.07 | 0.03 | 0.11 | 0.12 |  |
|  | 40-64 |  | 0.54 | 0.19 | 0.16 | 0.30 | 0.34 | 0.26 | 0.26 | 0.15 | 0.03 | 0.12 |  |
|  | 65+ |  | 0.69 | 0.46 | **0.58** | 0.57 | 0.78 | 0.50 | 1.12 | 0.62 | 0.03 | 0.72 |  |
| Germany, Former Federal Republic | 0-4 |  | 0.55 | 0.65 | 0.09 | 0.29 | 0.49 | 0.28 | 0.15 |  |  |  |  |
|  | 5-14 |  | 0.03 | 0.01 | -0.01 | 0.05 | 0.04 | 0.04 | 0.02 |  |  |  |  |
|  | 15-39 |  | 0.15 | 0.12 | 0.00 | 0.08 | 0.09 | 0.16 | 0.05 |  |  |  |  |
|  | 40-64 |  | 0.22 | 0.09 | 0.01 | 0.28 | 0.37 | 0.32 | 0.21 |  |  |  |  |
|  | 65+ |  | 0.44 | 0.52 | -0.06 | **0.55** | 0.84 | 0.73 | 0.48 |  |  |  |  |
| Germany | 0-4 |  |  |  |  |  |  |  |  | 0.14 | 0.06 | 0.05 | 0.04 |
|  | 5-14 |  |  |  |  |  |  |  |  | 0.03 | 0.02 | 0.01 | 0.00 |
|  | 15-39 |  |  |  |  |  |  |  |  | 0.08 | 0.10 | 0.11 | 0.03 |
|  | 40-64 |  |  |  |  |  |  |  |  | 0.23 | 0.29 | 0.18 | 0.12 |
|  | 65+ |  |  |  |  |  |  |  |  | **0.70** | 0.82 | 0.51 | 0.61 |
| Greece | 0-4 |  |  |  | 0.53 | 0.51 | 0.45 | 0.34 | 0.27 | 0.15 | 0.19 | 0.09 | 0.05 |
|  | 5-14 |  |  |  | 0.05 | 0.02 | 0.04 | 0.03 | 0.01 | 0.01 | 0.01 | 0.01 | 0.00 |
|  | 15-39 |  |  |  | 0.12 | 0.08 | 0.07 | 0.02 | 0.04 | 0.04 | 0.04 | 0.05 | 0.01 |
|  | 40-64 |  |  |  | 0.19 | 0.20 | 0.20 | 0.22 | 0.21 | 0.17 | 0.13 | 0.13 | 0.06 |
|  | 65+ |  |  |  | 0.49 | 0.35 | 0.21 | 0.25 | **0.43** | 0.31 | 0.29 | 0.66 | 0.83 |
| Hong Kong SAR | 0-4 |  |  | 1.83 | 0.62 | 0.32 | 0.29 | 0.33 | 0.15 | 0.15 | 0.15 | 0.06 | 0.04 |
|  | 5-14 |  |  | 0.16 | 0.10 | 0.01 | 0.04 | 0.02 | 0.04 | 0.02 | 0.03 | 0.01 | 0.01 |
|  | 15-39 |  |  | 0.33 | 0.21 | 0.08 | 0.25 | 0.24 | 0.01 | 0.11 | 0.06 | 0.00 | 0.07 |
|  | 40-64 |  |  | 0.38 | 0.24 | 0.26 | 0.42 | 0.63 | 0.44 | 0.42 | 0.31 | 0.21 | 0.10 |
|  | 65+ |  |  | 0.59 | -0.05 | **0.50** | 0.34 | 1.56 | -0.16 | 0.79 | 0.80 | 0.85 | 0.92 |
| Hungary | 0-4 |  | 0.86 | 0.63 | 0.26 | 0.25 | 0.72 | 0.16 | 0.29 | 0.30 | 0.18 | 0.19 | 0.06 |
|  | 5-14 |  | 0.09 | 0.05 | 0.00 | 0.00 | 0.03 | 0.01 | -0.01 | 0.03 | 0.02 | 0.02 | 0.01 |
|  | 15-39 |  | 0.27 | 0.24 | 0.04 | -0.01 | -0.06 | -0.06 | -0.02 | 0.13 | 0.22 | 0.14 | 0.11 |
|  | 40-64 |  | 0.32 | 0.31 | -0.11 | -0.11 | -0.29 | -0.04 | 0.02 | 0.06 | 0.29 | 0.35 | 0.30 |
|  | 65+ |  | 0.21 | 0.39 | -0.04 | 0.25 | 0.02 | **0.23** | 0.35 | 0.31 | 0.39 | 0.78 | 0.69 |
| Ireland | 0-4 | 0.76 | 0.45 | 0.31 | 0.46 | 0.23 | 0.29 | 0.25 | 0.14 | 0.08 | 0.02 | 0.14 |  |
|  | 5-14 | 0.16 | 0.06 | 0.03 | 0.03 | 0.03 | 0.01 | 0.02 | 0.02 | 0.03 | -0.03 | 0.02 |  |
|  | 15-39 | 1.27 | 0.31 | 0.22 | 0.05 | 0.14 | 0.12 | 0.08 | -0.02 | 0.03 | -0.02 | 0.09 |  |
|  | 40-64 | 0.72 | 0.41 | 0.16 | 0.09 | 0.26 | 0.29 | 0.35 | 0.41 | 0.32 | 0.21 | 0.33 |  |
|  | 65+ | 0.58 | 0.35 | 0.27 | 0.23 | 0.00 | **0.53** | 0.46 | 0.56 | 0.43 | 0.58 | 1.66 |  |

| **Country** | **Age groups** | **1950-55** | **1955-60** | **1960-65** | **1965-70** | **1970-75** | **1975-80** | **1980-85** | **1985-90** | **1990-95** | **1995-2000** | **2000-05** | **2005-10** |
| --- | --- | --- | --- | --- | --- | --- | --- | --- | --- | --- | --- | --- | --- |
| Israel | 0-4 |  |  |  |  |  | 0.40 | 0.29 | 0.21 | 0.24 | 0.13 | 0.09 | 0.07 |
|  | 5-14 |  |  |  |  |  | 0.06 | 0.03 | 0.00 | 0.05 | 0.00 | 0.02 | 0.00 |
|  | 15-39 |  |  |  |  |  | 0.17 | 0.06 | 0.04 | 0.07 | 0.03 | 0.05 | 0.08 |
|  | 40-64 |  |  |  |  |  | 0.40 | 0.32 | 0.25 | 0.33 | 0.33 | 0.27 | 0.20 |
|  | 65+ |  |  |  |  |  | 0.15 | **0.51** | 0.90 | 0.65 | 0.85 | 0.68 | 1.19 |
| Italy | 0-4 |  | 0.68 | 0.75 | 0.50 | 0.70 | 0.45 | 0.31 | 0.16 | 0.13 | 0.14 |  |  |
|  | 5-14 |  | 0.06 | 0.06 | 0.03 | 0.04 | 0.02 | 0.04 | 0.02 | -0.01 | 0.03 |  |  |
|  | 15-39 |  | 0.21 | 0.16 | 0.09 | 0.13 | 0.09 | 0.09 | 0.00 | -0.02 | 0.13 |  |  |
|  | 40-64 |  | 0.31 | 0.14 | 0.13 | 0.33 | 0.31 | 0.28 | 0.29 | 0.20 | 0.22 |  |  |
|  | 65+ |  | 0.73 | 0.17 | 0.31 | 0.47 | **0.79** | 0.42 | 0.93 | 0.59 | 0.70 |  |  |
| Japan | 0-4 | 2.12 | 1.29 | 1.06 | 0.49 | 0.27 | 0.22 | 0.16 | 0.09 | 0.06 | 0.08 | 0.05 | 0.00 |
|  | 5-14 | 0.32 | 0.18 | 0.13 | 0.05 | 0.05 | 0.04 | 0.02 | 0.01 | 0.01 | 0.02 | 0.01 | -0.01 |
|  | 15-39 | 1.70 | 0.67 | 0.55 | 0.20 | 0.22 | 0.19 | 0.08 | 0.07 | 0.04 | 0.01 | 0.01 | 0.00 |
|  | 40-64 | 1.00 | 0.60 | 0.60 | 0.46 | 0.56 | 0.50 | 0.31 | 0.25 | 0.12 | 0.16 | 0.15 | 0.09 |
|  | 65+ | 0.57 | 0.24 | 0.44 | **0.63** | 0.80 | 1.24 | 1.18 | 0.99 | 0.94 | 1.14 | 0.67 | 1.17 |
| Kazakhstan | 0-4 |  |  |  |  |  |  |  | 0.57 | -0.26 | 0.83 | 0.41 | -0.19 |
|  | 5-14 |  |  |  |  |  |  |  | 0.02 | -0.01 | 0.03 | 0.01 | 0.03 |
|  | 15-39 |  |  |  |  |  |  |  | -0.06 | -0.37 | -0.04 | -0.07 | 0.36 |
|  | 40-64 |  |  |  |  |  |  |  | 0.22 | -1.08 | 0.12 | 0.08 | 0.82 |
|  | 65+ |  |  |  |  |  |  |  | -0.08 | -1.15 | 0.15 | 0.01 | 0.60 |
| Kuwait | 0-4 |  |  |  |  |  | 0.71 | 1.02 |  |  | 0.19 |  |  |
|  | 5-14 |  |  |  |  |  | 0.05 | 0.06 |  |  | 0.00 |  |  |
|  | 15-39 |  |  |  |  |  | 0.23 | 0.22 |  |  | 0.10 |  |  |
|  | 40-64 |  |  |  |  |  | 0.08 | 0.44 |  |  | 0.69 |  |  |
|  | 65+ |  |  |  |  |  | 0.17 | 0.64 |  |  | 1.84 |  |  |
| Kyrgyzstan | 0-4 |  |  |  |  |  |  |  | 1.26 | 0.47 | 0.53 | -0.21 |  |
|  | 5-14 |  |  |  |  |  |  |  | 0.05 | 0.01 | 0.05 | 0.04 |  |
|  | 15-39 |  |  |  |  |  |  |  | -0.07 | -0.20 | 0.29 | 0.00 |  |
|  | 40-64 |  |  |  |  |  |  |  | 0.00 | -1.08 | 0.76 | -0.15 |  |
|  | 65+ |  |  |  |  |  |  |  | -0.31 | -1.35 | 0.54 | -0.18 |  |
| Latvia | 0-4 |  |  |  |  |  |  | 0.09 | 0.11 | -0.34 | 0.50 | 0.23 | 0.13 |
|  | 5-14 |  |  |  |  |  |  | 0.06 | -0.04 | 0.04 | 0.06 | 0.01 | 0.00 |
|  | 15-39 |  |  |  |  |  |  | 0.21 | -0.09 | -0.15 | 0.19 | 0.12 | 0.22 |
|  | 40-64 |  |  |  |  |  |  | 0.03 | 0.00 | -0.96 | 0.97 | 0.02 | 0.61 |
|  | 65+ |  |  |  |  |  |  | -0.19 | 0.37 | -0.09 | 0.70 | 0.05 | 1.07 |
| Lithuania | 0-4 |  |  |  |  |  |  |  | 0.08 | -0.02 | 0.30 | 0.15 | 0.23 |
|  | 5-14 |  |  |  |  |  |  |  | 0.00 | 0.03 | 0.06 | 0.01 | 0.02 |
|  | 15-39 |  |  |  |  |  |  |  | -0.02 | -0.12 | 0.17 | 0.09 | 0.18 |
|  | 40-64 |  |  |  |  |  |  |  | -0.09 | -0.62 | 0.85 | -0.17 | 0.41 |
|  | 65+ |  |  |  |  |  |  |  | 0.36 | -0.16 | 0.68 | 0.02 | 0.44 |
| Mauritius | 0-4 |  |  |  | 0.22 | 0.79 | 1.70 | 0.78 | 0.47 | 0.10 | 0.22 | 0.10 |  |
|  | 5-14 |  |  |  | 0.08 | 0.18 | 0.08 | 0.04 | 0.07 | 0.00 | 0.05 | 0.02 |  |
|  | 15-39 |  |  |  | 0.71 | 0.27 | 0.50 | 0.09 | 0.34 | 0.19 | 0.08 | 0.06 |  |
|  | 40-64 |  |  |  | 0.13 | 0.39 | 0.32 | 0.28 | 0.37 | -0.10 | 0.18 | 0.27 |  |
|  | 65+ |  |  |  | 0.08 | -0.18 | 0.65 | 0.35 | 0.42 | **0.60** | 0.27 | 0.17 |  |
| Mexico | 0-4 |  |  | 1.22 | 0.33 | 1.98 |  |  | 0.13 | 0.64 |  |  |  |
|  | 5-14 |  |  | 0.14 | 0.15 | 0.31 |  |  | 0.01 | 0.09 |  |  |  |
|  | 15-39 |  |  | 0.36 | 0.35 | 0.62 |  |  | 0.22 | 0.15 |  |  |  |
|  | 40-64 |  |  | 0.27 | 0.15 | 0.63 |  |  | 0.31 | 0.05 |  |  |  |
|  | 65+ |  |  | -0.41 | 1.03 | 0.12 |  |  | 0.26 | -0.08 |  |  |  |
| Netherlands | 0-4 | 0.45 | 0.30 | 0.15 | 0.16 | 0.15 | 0.20 | 0.06 | 0.12 | 0.11 |  | 0.06 | 0.08 |
|  | 5-14 | 0.04 | 0.03 | 0.00 | 0.02 | 0.03 | 0.04 | 0.03 | -0.01 | 0.02 |  | 0.01 | 0.01 |
|  | 15-39 | 0.22 | 0.13 | 0.02 | -0.03 | 0.08 | 0.06 | 0.04 | 0.00 | 0.01 |  | 0.09 | 0.06 |
|  | 40-64 | 0.32 | 0.32 | 0.10 | -0.01 | 0.27 | 0.17 | 0.10 | 0.07 | 0.03 |  | 0.18 | 0.21 |
|  | 65+ | 0.31 | **0.65** | 0.42 | 0.19 | 0.72 | 0.98 | 0.25 | 0.21 | 0.08 |  | 0.66 | 0.76 |
| New Zealand | 0-4 | 0.23 | 0.14 | 0.39 | 0.15 | 0.13 | 0.23 | 0.17 | 0.21 | 0.07 | 0.14 | 0.05 |  |
|  | 5-14 | 0.06 | 0.06 | 0.02 | 0.01 | 0.01 | 0.03 | 0.00 | 0.05 | 0.01 | 0.01 | 0.03 |  |
|  | 15-39 | 0.40 | 0.14 | 0.07 | -0.04 | 0.05 | 0.03 | 0.09 | 0.03 | 0.04 | 0.14 | 0.06 |  |
|  | 40-64 | 0.53 | 0.16 | 0.01 | 0.01 | 0.10 | 0.30 | 0.30 | 0.19 | 0.39 | 0.37 | 0.31 |  |
|  | 65+ | 0.55 | 0.11 | 0.07 | **0.31** | 0.36 | 0.44 | 0.53 | 0.68 | 0.53 | 0.77 | 0.52 |  |
| Norway | 0-4 |  | 0.22 | 0.14 | 0.23 | 0.19 | 0.23 | -0.05 | 0.07 | 0.25 | 0.05 | 0.06 | 0.06 |
|  | 5-14 |  | 0.03 | 0.03 | 0.00 | 0.02 | 0.05 | 0.00 | 0.01 | 0.02 | 0.01 | 0.01 | 0.01 |
|  | 15-39 |  | 0.12 | 0.08 | -0.03 | 0.07 | 0.04 | 0.01 | -0.02 | 0.08 | -0.05 | 0.06 | 0.04 |
|  | 40-64 |  | 0.16 | 0.12 | 0.08 | 0.13 | 0.16 | 0.03 | 0.10 | 0.10 | 0.14 | 0.21 | 0.19 |
|  | 65+ |  | 0.05 | **0.20** | 0.40 | 0.56 | 0.58 | 0.53 | 0.08 | 0.49 | 0.42 | 0.84 | 0.42 |
| Poland | 0-4 |  |  | 1.28 | 0.75 | 0.60 | 0.23 | 0.15 | 0.20 | 0.12 |  | 0.14 | 0.10 |
|  | 5-14 |  |  | 0.06 | 0.01 | 0.03 | 0.01 | 0.03 | 0.00 | 0.03 |  | 0.00 | 0.01 |
|  | 15-39 |  |  | 0.27 | 0.10 | 0.10 | 0.00 | 0.04 | 0.02 | 0.09 |  | 0.09 | 0.05 |
|  | 40-64 |  |  | 0.34 | 0.05 | 0.19 | -0.08 | -0.10 | 0.03 | 0.30 |  | 0.25 | 0.22 |
|  | 65+ |  |  | 0.17 | 0.00 | 0.65 | 0.02 | -0.27 | **0.29** | 0.33 |  | 0.94 | 0.64 |
| Portugal | 0-4 |  | 0.70 | 2.09 | 1.24 | 1.47 | 0.94 | 0.72 | 0.37 | 0.34 | 0.20 |  |  |
|  | 5-14 |  | 0.12 | 0.05 | 0.11 | 0.05 | 0.06 | 0.06 | 0.00 | 0.02 | 0.05 |  |  |
|  | 15-39 |  | 0.31 | 0.24 | 0.07 | 0.11 | 0.13 | 0.03 | 0.05 | 0.03 | 0.12 |  |  |
|  | 40-64 |  | 0.60 | 0.01 | 0.06 | 0.22 | 0.39 | 0.16 | 0.25 | 0.27 | 0.16 |  |  |
|  | 65+ |  | 0.69 | -0.04 | -0.21 | 0.33 | 0.75 | **0.97** | 0.49 | 0.68 | 0.62 |  |  |

| **Country** | **Age groups** | **1950-55** | **1955-60** | **1960-65** | **1965-70** | **1970-75** | **1975-80** | **1980-85** | **1985-90** | **1990-95** | **1995-2000** | **2000-05** | **2005-10** |
| --- | --- | --- | --- | --- | --- | --- | --- | --- | --- | --- | --- | --- | --- |
| Puerto Rico | 0-4 |  |  |  |  | 0.50 |  | 0.23 | 0.13 |  |  |  |  |
|  | 5-14 |  |  |  |  | 0.06 |  | 0.02 | 0.00 |  |  |  |  |
|  | 15-39 |  |  |  |  | 0.23 |  | -0.04 | -0.20 |  |  |  |  |
|  | 40-64 |  |  |  |  | 0.54 |  | 0.16 | -0.03 |  |  |  |  |
|  | 65+ |  |  |  |  | 0.41 |  | 0.34 | 0.10 |  |  |  |  |
| Republic of Korea | 0-4 |  |  |  |  |  |  |  | 0.17 | 0.03 | -0.09 | 0.09 | 0.12 |
|  | 5-14 |  |  |  |  |  |  |  | 0.13 | 0.06 | 0.06 | 0.04 | 0.03 |
|  | 15-39 |  |  |  |  |  |  |  | 0.28 | 0.12 | 0.21 | 0.12 | -0.04 |
|  | 40-64 |  |  |  |  |  |  |  | 0.48 | 0.41 | 0.50 | 0.45 | 0.32 |
|  | 65+ |  |  |  |  |  |  |  | **0.89** | -1.26 | 0.79 | 1.33 | 1.64 |
| Republic of Moldova | 0-4 |  |  |  |  |  |  |  | 0.62 | -0.14 | 0.35 | 0.37 | 0.18 |
|  | 5-14 |  |  |  |  |  |  |  | 0.01 | 0.00 | 0.04 | 0.04 | 0.02 |
|  | 15-39 |  |  |  |  |  |  |  | 0.06 | -0.06 | 0.16 | 0.09 | 0.14 |
|  | 40-64 |  |  |  |  |  |  |  | 0.52 | -0.60 | 0.66 | -0.04 | 0.74 |
|  | 65+ |  |  |  |  |  |  |  | 0.27 | -1.02 | 0.45 | 0.16 | 0.75 |
| Romania | 0-4 |  |  |  |  | 1.04 |  | 0.32 | -0.11 | 0.32 | 0.36 | 0.29 | 0.41 |
|  | 5-14 |  |  |  |  | 0.03 |  | -0.01 | 0.02 | -0.02 | 0.03 | 0.10 | 0.03 |
|  | 15-39 |  |  |  |  | 0.11 |  | 0.02 | 0.04 | 0.08 | 0.18 | 0.11 | 0.09 |
|  | 40-64 |  |  |  |  | 0.25 |  | 0.01 | 0.07 | -0.30 | 0.38 | 0.29 | 0.31 |
|  | 65+ |  |  |  |  | 0.44 |  | 0.14 | **0.41** | 0.06 | 0.56 | 0.44 | 0.69 |
| Russian Federation | 0-4 |  |  |  |  |  |  | 0.05 | 0.28 | 0.00 | 0.17 | 0.36 | 0.22 |
|  | 5-14 |  |  |  |  |  |  | 0.05 | 0.01 | -0.02 | 0.02 | 0.03 | 0.03 |
|  | 15-39 |  |  |  |  |  |  | 0.17 | 0.02 | -0.53 | -0.03 | -0.13 | 0.26 |
|  | 40-64 |  |  |  |  |  |  | 0.14 | 0.25 | -1.35 | 0.33 | -0.16 | 0.88 |
|  | 65+ |  |  |  |  |  |  | -0.11 | 0.34 | -0.68 | 0.10 | 0.19 | 0.80 |
| Singapore | 0-4 |  |  |  | 0.60 | 0.56 | 0.09 | 0.26 | 0.27 | 0.19 | 0.08 | 0.08 | 0.02 |
|  | 5-14 |  |  |  | 0.07 | 0.06 | 0.00 | 0.07 | 0.00 | 0.04 | 0.01 | 0.02 | 0.01 |
|  | 15-39 |  |  |  | 0.21 | 0.16 | 0.10 | 0.10 | 0.12 | 0.19 | 0.07 | 0.09 | 0.06 |
|  | 40-64 |  |  |  | 0.32 | 0.44 | 0.13 | 0.52 | 0.48 | 0.53 | 0.57 | 0.30 | 0.40 |
|  | 65+ |  |  |  | 0.56 | -0.01 | **0.36** | 0.76 | 1.21 | 0.78 | 0.82 | 1.57 | 0.78 |
| Slovenia | 0-4 |  |  |  |  |  |  |  | 0.30 | 0.16 | 0.13 | 0.01 | 0.06 |
|  | 5-14 |  |  |  |  |  |  |  | -0.01 | 0.02 | 0.04 | -0.02 | 0.02 |
|  | 15-39 |  |  |  |  |  |  |  | 0.05 | -0.01 | 0.18 | 0.07 | 0.13 |
|  | 40-64 |  |  |  |  |  |  |  | 0.23 | 0.16 | 0.28 | 0.40 | 0.40 |
|  | 65+ |  |  |  |  |  |  |  | **0.71** | 0.49 | 0.81 | 0.75 | 0.99 |
| Spain | 0-4 |  | 1.05 | 0.78 |  |  | 0.30 | 0.23 | 0.17 | 0.14 | 0.11 | 0.06 | 0.03 |
|  | 5-14 |  | 0.14 | 0.09 |  |  | 0.03 | 0.03 | 0.01 | 0.02 | 0.02 | 0.02 | 0.01 |
|  | 15-39 |  | 0.43 | 0.24 |  |  | 0.11 | 0.06 | -0.08 | 0.01 | 0.17 | 0.09 | 0.10 |
|  | 40-64 |  | 0.47 | 0.28 |  |  | 0.49 | 0.30 | 0.18 | 0.20 | 0.17 | 0.13 | 0.09 |
|  | 65+ |  | 0.41 | 0.50 |  |  | **1.14** | 0.68 | 0.53 | 0.80 | 0.53 | 0.69 | 0.75 |
| Sweden | 0-4 |  | 0.15 | 0.22 | 0.21 | 0.14 | 0.13 | 0.07 | 0.03 | 0.17 |  | 0.01 | 0.04 |
|  | 5-14 |  | 0.04 | 0.01 | 0.01 | 0.02 | 0.04 | 0.03 | 0.00 | 0.02 |  | 0.00 | 0.02 |
|  | 15-39 |  | 0.13 | 0.00 | 0.03 | 0.03 | 0.10 | 0.05 | 0.04 | 0.10 |  | -0.03 | 0.07 |
|  | 40-64 |  | 0.34 | 0.21 | 0.09 | 0.19 | 0.15 | 0.25 | 0.11 | 0.15 |  | 0.18 | 0.21 |
|  | 65+ |  | **0.39** | 0.59 | 0.60 | 0.47 | 0.55 | 0.61 | 0.43 | 0.54 |  | 0.61 | 0.37 |
| Switzerland | 0-4 |  | 0.44 | 0.27 | 0.25 | 0.30 | 0.24 | 0.08 | 0.03 | 0.14 | 0.02 | 0.06 | 0.02 |
|  | 5-14 |  | 0.02 | 0.04 | -0.02 | 0.05 | 0.02 | 0.03 | 0.03 | 0.01 | 0.01 | 0.00 | 0.02 |
|  | 15-39 |  | 0.15 | 0.11 | 0.00 | 0.07 | -0.02 | 0.07 | -0.02 | 0.05 | 0.16 | 0.09 | 0.10 |
|  | 40-64 |  | 0.51 | 0.23 | 0.18 | 0.44 | 0.13 | 0.28 | 0.18 | 0.10 | 0.19 | 0.21 | 0.12 |
|  | 65+ |  | **0.74** | 0.26 | 0.45 | 1.07 | 0.70 | 0.82 | 0.51 | 0.53 | 0.48 | 0.82 | 0.34 |
| TFYR Macedonia | 0-4 |  |  |  |  |  |  |  |  |  | 0.70 | 0.01 | 0.23 |
|  | 5-14 |  |  |  |  |  |  |  |  |  | 0.05 | 0.02 | 0.02 |
|  | 15-39 |  |  |  |  |  |  |  |  |  | 0.09 | 0.09 | 0.06 |
|  | 40-64 |  |  |  |  |  |  |  |  |  | 0.12 | 0.18 | 0.23 |
|  | 65+ |  |  |  |  |  |  |  |  |  | -0.08 | 0.17 | 0.43 |
| Tajikistan | 0-4 |  |  |  |  |  |  |  | 0.30 | 1.49 | 2.51 |  |  |
|  | 5-14 |  |  |  |  |  |  |  | 0.03 | -0.10 | 0.18 |  |  |
|  | 15-39 |  |  |  |  |  |  |  | 0.18 | -0.35 | 0.48 |  |  |
|  | 40-64 |  |  |  |  |  |  |  | -0.07 | -0.77 | 0.79 |  |  |
|  | 65+ |  |  |  |  |  |  |  | -0.31 | -1.30 | 0.17 |  |  |
| Trinidad and Tobago | 0-4 |  |  |  |  | 1.20 | 1.42 | 0.41 | 0.17 |  |  |  |  |
|  | 5-14 |  |  |  |  | 0.04 | 0.02 | 0.05 | -0.04 |  |  |  |  |
|  | 15-39 |  |  |  |  | 0.24 | 0.13 | 0.05 | 0.03 |  |  |  |  |
|  | 40-64 |  |  |  |  | 0.34 | 0.31 | -0.38 | 0.25 |  |  |  |  |
|  | 65+ |  |  |  |  | -0.09 | 0.32 | **1.19** | 0.30 |  |  |  |  |
| Turkmenistan | 0-4 |  |  |  |  |  |  |  | 1.66 | 0.29 |  |  |  |
|  | 5-14 |  |  |  |  |  |  |  | 0.03 | -0.02 |  |  |  |
|  | 15-39 |  |  |  |  |  |  |  | 0.04 | -0.07 |  |  |  |
|  | 40-64 |  |  |  |  |  |  |  | 0.06 | -0.39 |  |  |  |
|  | 65+ |  |  |  |  |  |  |  | -0.18 | -1.30 |  |  |  |
| Ukraine | 0-4 |  |  |  |  |  |  |  | 0.12 | -0.08 | 0.21 | 0.20 |  |
|  | 5-14 |  |  |  |  |  |  |  | 0.02 | -0.01 | 0.04 | 0.01 |  |
|  | 15-39 |  |  |  |  |  |  |  | -0.04 | -0.32 | 0.06 | -0.20 |  |
|  | 40-64 |  |  |  |  |  |  |  | -0.03 | -0.87 | 0.27 | -0.21 |  |
|  | 65+ |  |  |  |  |  |  |  | 0.18 | -0.74 | 0.31 | 0.11 |  |

| **Country** | **Age groups** | **1950-55** | **1955-60** | **1960-65** | **1965-70** | **1970-75** | **1975-80** | **1980-85** | **1985-90** | **1990-95** | **1995-2000** | **2000-05** | **2005-10** |
| --- | --- | --- | --- | --- | --- | --- | --- | --- | --- | --- | --- | --- | --- |
| United Kingdom | 0-4 | 0.47 | 0.19 | 0.17 | 0.14 | 0.14 | 0.34 | 0.19 | 0.13 | 0.14 |  |  | 0.04 |
|  | 5-14 | 0.08 | 0.02 | 0.01 | 0.02 | 0.02 | 0.02 | 0.02 | 0.02 | 0.02 |  |  | 0.00 |
|  | 15-39 | 0.51 | 0.16 | 0.04 | 0.07 | 0.04 | 0.07 | 0.08 | 0.01 | 0.02 |  |  | 0.03 |
|  | 40-64 | 0.46 | 0.20 | 0.10 | 0.00 | 0.09 | 0.20 | 0.29 | 0.31 | 0.30 |  |  | 0.18 |
|  | 65+ | **0.62** | 0.27 | 0.46 | 0.22 | 0.20 | 0.45 | 0.47 | 0.46 | 0.44 |  |  | 0.87 |
| United States of America | 0-4 | 0.22 | 0.05 | 0.07 | 0.34 | 0.28 | 0.26 | 0.16 | 0.07 | 0.16 | 0.07 | 0.02 |  |
|  | 5-14 | 0.06 | 0.01 | 0.02 | 0.01 | 0.03 | 0.02 | 0.02 | 0.01 | 0.01 | 0.02 | 0.01 |  |
|  | 15-39 | 0.31 | 0.07 | -0.03 | -0.01 | 0.20 | 0.12 | 0.09 | -0.02 | -0.01 | 0.09 | 0.02 |  |
|  | 40-64 | 0.56 | 0.19 | 0.06 | 0.09 | 0.39 | 0.37 | 0.18 | 0.18 | 0.08 | 0.16 | 0.13 |  |
|  | 65+ | 0.50 | 0.16 | **0.19** | 0.47 | 0.90 | 0.57 | 0.07 | 0.29 | 0.03 | 0.06 | 0.64 |  |
| Uruguay | 0-4 |  | -0.63 |  | 0.24 | -0.10 |  | 0.49 | 0.64 |  |  |  |  |
|  | 5-14 |  | 0.00 |  | 0.04 | -0.01 |  | 0.01 | 0.05 |  |  |  |  |
|  | 15-39 |  | 0.29 |  | 0.09 | -0.02 |  | 0.21 | 0.03 |  |  |  |  |
|  | 40-64 |  | 0.11 |  | -0.06 | 0.28 |  | 0.32 | 0.06 |  |  |  |  |
|  | 65+ |  | -0.56 |  | -0.53 | 0.70 |  | 0.17 | 0.25 |  |  |  |  |
| Uzbekistan | 0-4 |  |  |  |  |  |  |  | 0.85 | 0.94 | 0.81 | 0.53 |  |
|  | 5-14 |  |  |  |  |  |  |  | 0.05 | -0.01 | 0.06 | 0.06 |  |
|  | 15-39 |  |  |  |  |  |  |  | 0.20 | -0.13 | 0.13 | 0.16 |  |
|  | 40-64 |  |  |  |  |  |  |  | -0.09 | -0.63 | 0.52 | 0.09 |  |
|  | 65+ |  |  |  |  |  |  |  | -0.16 | -1.50 | 0.23 | 0.04 |  |
| Venezuela | 0-4 |  | 2.30 | 0.59 | 0.05 | 0.71 | 1.09 |  | 0.16 |  |  |  |  |
|  | 5-14 |  | 0.36 | 0.07 | 0.08 | 0.06 | 0.13 |  | 0.02 |  |  |  |  |
|  | 15-39 |  | 0.55 | 0.37 | 0.25 | 0.24 | 0.30 |  | 0.03 |  |  |  |  |
|  | 40-64 |  | 0.58 | 0.36 | 0.16 | 0.35 | 0.80 |  | 0.22 |  |  |  |  |
|  | 65+ |  | 0.58 | 0.08 | -0.43 | -0.16 | 0.38 |  | 0.29 |  |  |  |  |
| Yugoslavia, Former | 0-4 |  |  |  | 1.10 | 1.66 | 0.65 | 0.31 | 0.50 |  |  |  |  |
|  | 5-14 |  |  |  | 0.03 | 0.04 | 0.02 | 0.03 | 0.03 |  |  |  |  |
|  | 15-39 |  |  |  | 0.24 | 0.16 | 0.10 | 0.05 | 0.06 |  |  |  |  |
|  | 40-64 |  |  |  | 0.06 | 0.35 | 0.12 | 0.04 | 0.22 |  |  |  |  |
|  | 65+ |  |  |  | -0.25 | 0.41 | 0.18 | 0.13 | 0.58 |  |  |  |  |
| Croatia | 0-4 |  |  |  |  |  |  |  | 0.33 | 0.20 | 0.01 | 0.16 | 0.10 |
|  | 5-14 |  |  |  |  |  |  |  | 0.04 | 0.02 | 0.04 | 0.02 | 0.00 |
|  | 15-39 |  |  |  |  |  |  |  | -0.02 | 0.10 | 0.16 | 0.01 | 0.08 |
|  | 40-64 |  |  |  |  |  |  |  | 0.13 | 0.23 | 0.25 | 0.40 | 0.16 |
|  | 65+ |  |  |  |  |  |  |  | 0.37 | 0.18 | -0.28 | 1.44 | 0.58 |
| Serbia | 0-4 |  |  |  |  |  |  |  |  |  |  | 0.23 | 0.06 |
|  | 5-14 |  |  |  |  |  |  |  |  |  |  | 0.04 | 0.00 |
|  | 15-39 |  |  |  |  |  |  |  |  |  |  | 0.15 | 0.05 |
|  | 40-64 |  |  |  |  |  |  |  |  |  |  | 0.25 | 0.39 |
|  | 65+ |  |  |  |  |  |  |  |  |  |  | 0.39 | 0.75 |

* The most positive contributing cause of death as well as age group of at least 0.10 life years was marked with green for each year. The most decreasing life expectancy of at least -0.10 life-years was marked red, respectively. ‘Other diseases’ were not considered. For each country the first of at least two following years with cardiovascular disease being the most positively contributing cause of death to the improvement in live expectancy was printed with bold letters as an indicator for the epidemiologic transition. Correspondingly, the same procedure was performed for the age group 65 years and older.

**Table S4:** Relative change in life expectancy [years] by five-year interval in men according to age of death*

| **Country** | **Age groups** | **1950-55** | **1955-60** | **1960-65** | **1965-70** | **1970-75** | **1975-80** | **1980-85** | **1985-90** | **1990-95** | **1995-2000** | **2000-05** | **2005-10** |
| --- | --- | --- | --- | --- | --- | --- | --- | --- | --- | --- | --- | --- | --- |
| Argentina | 0-4 |  |  |  |  |  |  | 1.55 | 0.14 | 0.33 |  |  |  |
|  | 5-14 |  |  |  |  |  |  | 0.06 | 0.03 | 0.03 |  |  |  |
|  | 15-39 |  |  |  |  |  |  | 0.22 | 0.06 | -0.21 |  |  |  |
|  | 40-64 |  |  |  |  |  |  | 0.38 | 0.32 | 0.25 |  |  |  |
|  | 65+ |  |  |  |  |  |  | 0.43 | 0.14 | 0.06 |  |  |  |
| Armenia | 0-4 |  |  |  |  |  |  |  | 0.56 | 0.38 | -0.05 |  |  |
|  | 5-14 |  |  |  |  |  |  |  | 0.10 | 0.07 | 0.06 |  |  |
|  | 15-39 |  |  |  |  |  |  |  | -0.40 | -0.42 | 1.10 |  |  |
|  | 40-64 |  |  |  |  |  |  |  | -0.78 | -0.05 | 1.16 |  |  |
|  | 65+ |  |  |  |  |  |  |  | -0.64 | 0.44 | 1.11 |  |  |
| Australia | 0-4 | 0.26 | 0.21 | 0.18 | 0.08 | 0.26 | 0.39 | 0.12 | 0.18 | 0.19 | 0.05 |  |  |
|  | 5-14 | 0.13 | 0.04 | 0.02 | 0.02 | 0.04 | 0.04 | 0.03 | 0.04 | 0.02 | 0.02 |  |  |
|  | 15-39 | 0.18 | 0.12 | 0.00 | -0.06 | 0.11 | 0.18 | 0.14 | 0.04 | 0.09 | 0.10 |  |  |
|  | 40-64 | 0.31 | 0.06 | -0.21 | 0.07 | 0.34 | 0.83 | 0.64 | 0.61 | 0.53 | 0.52 |  |  |
|  | 65+ | 0.06 | 0.11 | -0.19 | 0.01 | 0.37 | 0.83 | 0.49 | 0.60 | **0.64** | 1.19 |  |  |
| Austria | 0-4 |  | 0.68 | 0.68 | 0.22 | 0.44 | 0.59 | 0.25 | 0.30 | 0.22 | 0.08 | 0.09 | 0.01 |
|  | 5-14 |  | 0.05 | 0.01 | -0.01 | 0.08 | 0.04 | 0.05 | 0.03 | 0.02 | 0.04 | 0.00 | 0.02 |
|  | 15-39 |  | 0.08 | 0.22 | -0.18 | 0.14 | 0.11 | 0.21 | 0.27 | 0.12 | 0.27 | 0.16 | 0.17 |
|  | 40-64 |  | 0.06 | 0.15 | -0.08 | 0.24 | 0.13 | 0.40 | 0.60 | 0.43 | 0.60 | 0.40 | 0.26 |
|  | 65+ |  | 0.08 | -0.06 | -0.26 | 0.37 | 0.43 | **0.54** | 0.61 | 0.59 | 0.67 | 0.88 | 0.54 |
| Azerbaijan | 0-4 |  |  |  |  |  |  |  | 0.17 | 0.55 | 1.15 |  |  |
|  | 5-14 |  |  |  |  |  |  |  | 0.04 | -0.05 | 0.07 |  |  |
|  | 15-39 |  |  |  |  |  |  |  | -0.21 | -1.28 | 1.41 |  |  |
|  | 40-64 |  |  |  |  |  |  |  | -0.23 | -0.57 | 1.16 |  |  |
|  | 65+ |  |  |  |  |  |  |  | 0.46 | -0.37 | 0.20 |  |  |
| Belarus | 0-4 |  |  |  |  |  |  |  | 0.14 | -0.06 |  |  |  |
|  | 5-14 |  |  |  |  |  |  |  | 0.01 | 0.02 |  |  |  |
|  | 15-39 |  |  |  |  |  |  |  | -0.35 | -0.70 |  |  |  |
|  | 40-64 |  |  |  |  |  |  |  | -0.53 | -1.67 |  |  |  |
|  | 65+ |  |  |  |  |  |  |  | 0.01 | -0.63 |  |  |  |
| Belgium | 0-4 |  | 0.90 | 0.50 | 0.21 | 0.43 | 0.38 | 0.19 | 0.16 | 0.17 |  |  |  |
|  | 5-14 |  | 0.02 | 0.02 | 0.00 | 0.04 | 0.05 | 0.02 | 0.05 | 0.02 |  |  |  |
|  | 15-39 |  | -0.01 | 0.00 | 0.04 | 0.06 | -0.05 | 0.16 | 0.06 | 0.05 |  |  |  |
|  | 40-64 |  | 0.11 | -0.04 | 0.06 | 0.40 | 0.28 | 0.44 | 0.63 | 0.19 |  |  |  |
|  | 65+ |  | 0.13 | -0.14 | -0.24 | 0.14 | **0.45** | 0.36 | 0.64 | 0.49 |  |  |  |
| Bulgaria | 0-4 |  |  |  | 0.34 | 0.31 | 0.34 | 0.39 | 0.00 | 0.01 | 0.17 | 0.32 | 0.15 |
|  | 5-14 |  |  |  | 0.02 | 0.03 | 0.01 | 0.02 | 0.01 | 0.02 | 0.06 | 0.01 | 0.03 |
|  | 15-39 |  |  |  | -0.08 | 0.03 | -0.05 | -0.08 | -0.02 | -0.15 | 0.32 | 0.13 | 0.13 |
|  | 40-64 |  |  |  | -0.42 | -0.09 | -0.44 | -0.40 | -0.28 | -0.73 | 0.41 | 0.05 | 0.52 |
|  | 65+ |  |  |  | -0.68 | -0.09 | -0.02 | -0.10 | 0.09 | -0.08 | 0.14 | 0.16 | 0.45 |
| Canada | 0-4 | 0.72 | 0.38 | 0.33 | 0.40 | 0.44 | 0.33 | 0.24 | 0.08 | 0.11 | 0.09 | 0.01 |  |
|  | 5-14 | 0.10 | 0.05 | 0.04 | 0.02 | 0.04 | 0.04 | 0.07 | 0.03 | 0.03 | 0.02 | 0.01 |  |
|  | 15-39 | 0.14 | 0.08 | -0.05 | 0.00 | -0.08 | 0.19 | 0.30 | 0.00 | 0.20 | 0.25 | 0.08 |  |
|  | 40-64 | 0.26 | 0.01 | -0.01 | 0.13 | 0.11 | 0.52 | 0.61 | 0.44 | 0.47 | 0.43 | 0.24 |  |
|  | 65+ | 0.10 | -0.06 | 0.16 | 0.08 | 0.03 | 0.58 | 0.27 | 0.43 | **0.53** | 0.59 | 0.84 |  |
| Chile | 0-4 |  | -0.07 | 1.54 | 1.89 | 1.42 | 2.03 | 0.95 | 0.33 |  |  |  |  |
|  | 5-14 |  | 0.01 | 0.14 | 0.11 | 0.06 | 0.10 | 0.10 | 0.05 |  |  |  |  |
|  | 15-39 |  | 0.36 | 0.35 | 0.32 | 0.80 | 0.25 | 0.26 | 0.12 |  |  |  |  |
|  | 40-64 |  | 0.41 | 0.30 | 0.11 | 1.20 | 0.39 | 0.27 | 0.45 |  |  |  |  |
|  | 65+ |  | 0.06 | -0.08 | 0.05 | 0.20 | 0.22 | 0.03 | 0.67 |  |  |  |  |
| China,select rural areas | 0-4 |  |  |  |  |  |  |  |  | 0.39 | 0.56 |  |  |
|  | 5-14 |  |  |  |  |  |  |  |  | -0.01 | 0.08 |  |  |
|  | 15-39 |  |  |  |  |  |  |  |  | 0.03 | 0.18 |  |  |
|  | 40-64 |  |  |  |  |  |  |  |  | 0.03 | 0.30 |  |  |
|  | 65+ |  |  |  |  |  |  |  |  | 0.50 | 0.20 |  |  |
| China,select urban and rural areas | 0-4 |  |  |  |  |  |  |  |  | 0.32 | 0.45 |  |  |
|  | 5-14 |  |  |  |  |  |  |  |  | 0.01 | 0.06 |  |  |
|  | 15-39 |  |  |  |  |  |  |  |  | 0.07 | 0.11 |  |  |
|  | 40-64 |  |  |  |  |  |  |  |  | 0.11 | 0.25 |  |  |
|  | 65+ |  |  |  |  |  |  |  |  | 0.46 | 0.34 |  |  |
| China,select urban areas | 0-4 |  |  |  |  |  |  |  |  | 0.31 | 0.33 |  |  |
|  | 5-14 |  |  |  |  |  |  |  |  | 0.03 | 0.05 |  |  |
|  | 15-39 |  |  |  |  |  |  |  |  | 0.09 | 0.08 |  |  |
|  | 40-64 |  |  |  |  |  |  |  |  | 0.17 | 0.23 |  |  |
|  | 65+ |  |  |  |  |  |  |  |  | **0.42** | 0.43 |  |  |

| **Country** | **Age groups** | **1950-55** | **1955-60** | **1960-65** | **1965-70** | **1970-75** | **1975-80** | **1980-85** | **1985-90** | **1990-95** | **1995-2000** | **2000-05** | **2005-10** |
| --- | --- | --- | --- | --- | --- | --- | --- | --- | --- | --- | --- | --- | --- |
| Costa Rica | 0-4 |  |  |  | 0.99 | 2.43 | 1.41 | 0.33 | 0.31 | 0.12 |  |  |  |
|  | 5-14 |  |  |  | 0.11 | 0.17 | 0.02 | 0.08 | 0.06 | 0.00 |  |  |  |
|  | 15-39 |  |  |  | 0.00 | -0.01 | 0.48 | 0.40 | 0.05 | -0.22 |  |  |  |
|  | 40-64 |  |  |  | 0.14 | 0.26 | 0.56 | 0.33 | 0.14 | -0.11 |  |  |  |
|  | 65+ |  |  |  | -0.09 | 0.98 | -0.57 | -1.11 | **0.96** | 0.18 |  |  |  |
| Cuba | 0-4 |  |  |  |  | 1.17 | 0.58 | 0.36 | 0.38 | 0.17 |  |  |  |
|  | 5-14 |  |  |  |  | 0.03 | -0.06 | 0.01 | 0.05 | 0.03 |  |  |  |
|  | 15-39 |  |  |  |  | 0.11 | -0.18 | 0.01 | -0.16 | 0.09 |  |  |  |
|  | 40-64 |  |  |  |  | 0.12 | -0.19 | -0.05 | -0.06 | 0.02 |  |  |  |
|  | 65+ |  |  |  |  | 0.52 | 0.38 | 0.20 | 0.13 | -0.30 |  |  |  |
| Czechoslovakia, Former | 0-4 |  | 0.86 | 0.05 | 0.14 | 0.20 | 0.23 | 0.30 | 0.25 |  |  |  |  |
|  | 5-14 |  | 0.06 | 0.01 | 0.00 | 0.03 | 0.03 | 0.05 | 0.02 |  |  |  |  |
|  | 15-39 |  | 0.06 | 0.04 | -0.21 | 0.29 | 0.08 | 0.09 | -0.02 |  |  |  |  |
|  | 40-64 |  | 0.36 | 0.04 | -0.68 | 0.03 | -0.30 | -0.23 | -0.04 |  |  |  |  |
|  | 65+ |  | 0.13 | -0.23 | -0.42 | 0.08 | 0.02 | 0.05 | 0.14 |  |  |  |  |
| Czech Republic | 0-4 |  |  |  |  |  |  |  |  | 0.26 | 0.27 | 0.07 | 0.08 |
|  | 5-14 |  |  |  |  |  |  |  |  | 0.02 | 0.04 | 0.02 | 0.03 |
|  | 15-39 |  |  |  |  |  |  |  |  | 0.08 | 0.23 | 0.13 | 0.16 |
|  | 40-64 |  |  |  |  |  |  |  |  | 0.78 | 0.66 | 0.49 | 0.54 |
|  | 65+ |  |  |  |  |  |  |  |  | 0.73 | **0.70** | 0.54 | 0.65 |
| Slovakia | 0-4 |  |  |  |  |  |  |  |  |  | 0.24 | 0.08 | 0.12 |
|  | 5-14 |  |  |  |  |  |  |  |  |  | 0.00 | 0.05 | 0.01 |
|  | 15-39 |  |  |  |  |  |  |  |  |  | 0.10 | 0.16 | 0.17 |
|  | 40-64 |  |  |  |  |  |  |  |  |  | 0.28 | 0.52 | 0.44 |
|  | 65+ |  |  |  |  |  |  |  |  |  | 0.18 | 0.22 | 0.46 |
| Denmark | 0-4 |  | 0.36 | 0.30 | 0.34 | 0.36 | 0.21 | 0.09 | 0.01 | 0.22 | 0.12 | 0.05 |  |
|  | 5-14 |  | -0.03 | -0.02 | -0.01 | 0.06 | 0.05 | 0.02 | 0.02 | 0.03 | 0.00 | 0.03 |  |
|  | 15-39 |  | 0.08 | 0.02 | -0.02 | 0.00 | -0.12 | 0.06 | 0.11 | 0.04 | 0.20 | 0.17 |  |
|  | 40-64 |  | 0.00 | -0.30 | 0.02 | -0.08 | -0.03 | 0.14 | 0.20 | 0.27 | 0.57 | 0.33 |  |
|  | 65+ |  | -0.09 | -0.35 | 0.30 | -0.04 | 0.01 | 0.21 | 0.16 | 0.07 | **0.83** | 0.70 |  |
| Estonia | 0-4 |  |  |  |  |  |  |  | 0.08 | 0.11 | 0.35 | 0.34 | 0.14 |
|  | 5-14 |  |  |  |  |  |  |  | -0.06 | 0.05 | 0.10 | 0.08 | 0.03 |
|  | 15-39 |  |  |  |  |  |  |  | -0.42 | -0.92 | 1.02 | 0.41 | 0.60 |
|  | 40-64 |  |  |  |  |  |  |  | -0.25 | -1.83 | 1.14 | 0.86 | 1.89 |
|  | 65+ |  |  |  |  |  |  |  | 0.26 | -0.22 | 0.40 | 0.24 | 0.80 |
| Finland | 0-4 |  | 0.63 | 0.46 | 0.32 | 0.23 | 0.24 | 0.14 | 0.03 | 0.18 | 0.04 | 0.03 | 0.08 |
|  | 5-14 |  | 0.08 | 0.00 | 0.02 | 0.08 | 0.05 | 0.05 | 0.01 | 0.03 | 0.02 | 0.00 | 0.03 |
|  | 15-39 |  | 0.17 | 0.13 | -0.03 | 0.07 | 0.30 | 0.12 | -0.24 | 0.40 | 0.14 | 0.18 | 0.06 |
|  | 40-64 |  | 0.16 | -0.24 | -0.05 | 0.57 | 0.82 | 0.48 | 0.43 | 0.72 | 0.42 | 0.20 | 0.50 |
|  | 65+ |  | 0.19 | -0.19 | 0.03 | 0.41 | 0.48 | 0.36 | **0.44** | 0.58 | 0.69 | 0.99 | 0.62 |
| France | 0-4 |  | 0.82 | 0.53 | 0.28 | 0.22 | 0.25 | 0.17 | 0.10 | 0.23 | 0.08 | 0.08 |  |
|  | 5-14 |  | 0.02 | 0.01 | -0.03 | 0.03 | 0.03 | 0.05 | 0.04 | 0.02 | 0.02 | 0.03 |  |
|  | 15-39 |  | 0.16 | -0.03 | -0.03 | 0.09 | 0.04 | 0.09 | -0.02 | 0.16 | 0.32 | 0.28 |  |
|  | 40-64 |  | 0.48 | 0.06 | 0.12 | 0.17 | 0.39 | 0.30 | 0.54 | 0.36 | 0.24 | 0.42 |  |
|  | 65+ |  | 0.37 | 0.01 | 0.26 | **0.31** | 0.50 | 0.37 | 0.91 | 0.46 | 0.14 | 0.84 |  |
| Germany, Former Federal Republic | 0-4 |  | 0.61 | 0.74 | 0.06 | 0.36 | 0.60 | 0.33 | 0.15 |  |  |  |  |
|  | 5-14 |  | 0.04 | 0.01 | 0.00 | 0.07 | 0.04 | 0.07 | 0.03 |  |  |  |  |
|  | 15-39 |  | 0.08 | 0.12 | -0.11 | 0.15 | 0.17 | 0.34 | 0.06 |  |  |  |  |
|  | 40-64 |  | 0.01 | -0.03 | -0.03 | 0.21 | 0.31 | 0.40 | 0.39 |  |  |  |  |
|  | 65+ |  | -0.01 | -0.05 | -0.29 | 0.25 | 0.51 | **0.50** | 0.45 |  |  |  |  |
| Germany | 0-4 |  |  |  |  |  |  |  |  | 0.18 | 0.08 | 0.07 | 0.05 |
|  | 5-14 |  |  |  |  |  |  |  |  | 0.03 | 0.02 | 0.02 | 0.01 |
|  | 15-39 |  |  |  |  |  |  |  |  | 0.16 | 0.27 | 0.25 | 0.11 |
|  | 40-64 |  |  |  |  |  |  |  |  | 0.36 | 0.61 | 0.40 | 0.34 |
|  | 65+ |  |  |  |  |  |  |  |  | **0.51** | 0.85 | 0.84 | 0.72 |
| Greece | 0-4 |  |  |  | 0.47 | 0.48 | 0.52 | 0.37 | 0.40 | 0.15 | 0.18 | 0.18 | 0.02 |
|  | 5-14 |  |  |  | 0.06 | 0.03 | 0.04 | 0.04 | 0.03 | 0.02 | 0.02 | 0.00 | 0.03 |
|  | 15-39 |  |  |  | 0.17 | -0.03 | 0.07 | -0.07 | -0.06 | 0.00 | 0.08 | 0.09 | 0.05 |
|  | 40-64 |  |  |  | 0.23 | 0.09 | 0.11 | 0.12 | 0.22 | 0.09 | 0.06 | 0.26 | 0.15 |
|  | 65+ |  |  |  | 0.24 | 0.28 | 0.14 | 0.06 | 0.30 | **0.20** | 0.36 | 0.83 | 0.75 |
| Hong Kong SAR | 0-4 |  |  | 1.60 | 0.63 | 0.38 | 0.37 | 0.39 | 0.17 | 0.20 | 0.10 | 0.09 | 0.03 |
|  | 5-14 |  |  | 0.17 | 0.10 | 0.01 | 0.05 | 0.07 | 0.04 | 0.02 | 0.00 | 0.02 | 0.01 |
|  | 15-39 |  |  | 0.08 | 0.21 | 0.19 | 0.13 | 0.53 | -0.03 | 0.08 | 0.09 | 0.02 | 0.13 |
|  | 40-64 |  |  | 0.56 | 0.37 | 0.77 | 0.56 | 0.92 | 0.48 | 0.63 | 0.53 | 0.35 | 0.26 |
|  | 65+ |  |  | 0.41 | -0.22 | 0.57 | 0.51 | 1.63 | -0.17 | **0.71** | 0.51 | 0.51 | 0.71 |
| Hungary | 0-4 |  | 0.98 | 0.71 | 0.32 | 0.30 | 0.70 | 0.23 | 0.34 | 0.34 | 0.19 | 0.19 | 0.14 |
|  | 5-14 |  | 0.07 | 0.08 | -0.02 | 0.05 | -0.01 | 0.06 | -0.01 | 0.04 | 0.02 | 0.02 | 0.02 |
|  | 15-39 |  | 0.52 | 0.12 | -0.14 | 0.03 | -0.24 | -0.14 | -0.14 | 0.22 | 0.52 | 0.30 | 0.33 |
|  | 40-64 |  | 0.20 | 0.20 | -0.52 | -0.32 | -1.08 | -0.75 | -0.29 | -0.35 | 0.69 | 0.63 | 0.88 |
|  | 65+ |  | -0.10 | 0.09 | -0.41 | 0.06 | -0.23 | 0.08 | 0.16 | 0.07 | 0.24 | 0.51 | 0.47 |
| Ireland | 0-4 | 0.86 | 0.57 | 0.47 | 0.57 | 0.15 | 0.41 | 0.30 | 0.07 | 0.21 | 0.03 | 0.20 |  |
|  | 5-14 | 0.14 | 0.05 | 0.03 | -0.01 | 0.01 | 0.01 | 0.04 | 0.05 | 0.01 | 0.03 | 0.03 |  |
|  | 15-39 | 0.83 | 0.21 | 0.07 | 0.05 | -0.08 | 0.06 | 0.13 | 0.00 | -0.07 | -0.05 | 0.24 |  |
|  | 40-64 | 0.58 | 0.24 | -0.17 | -0.03 | 0.13 | 0.14 | 0.49 | 0.58 | 0.45 | 0.36 | 0.64 |  |
|  | 65+ | 0.39 | 0.01 | -0.07 | -0.05 | -0.05 | 0.11 | 0.11 | 0.49 | 0.36 | **0.69** | 1.58 |  |

| **Country** | **Age groups** | **1950-55** | **1955-60** | **1960-65** | **1965-70** | **1970-75** | **1975-80** | **1980-85** | **1985-90** | **1990-95** | **1995-2000** | **2000-05** | **2005-10** |
| --- | --- | --- | --- | --- | --- | --- | --- | --- | --- | --- | --- | --- | --- |
| Israel | 0-4 |  |  |  |  |  | 0.54 | 0.36 | 0.20 | 0.26 | 0.12 | 0.11 | 0.10 |
|  | 5-14 |  |  |  |  |  | 0.07 | 0.05 | 0.04 | 0.01 | 0.01 | 0.01 | 0.02 |
|  | 15-39 |  |  |  |  |  | 0.40 | 0.12 | 0.08 | -0.07 | -0.02 | 0.22 | 0.14 |
|  | 40-64 |  |  |  |  |  | 0.24 | 0.16 | 0.60 | 0.25 | 0.29 | 0.34 | 0.46 |
|  | 65+ |  |  |  |  |  | 0.20 | **0.42** | 0.53 | 0.41 | 0.61 | 0.80 | 1.00 |
| Italy | 0-4 |  | 0.69 | 0.70 | 0.53 | 0.78 | 0.51 | 0.36 | 0.21 | 0.17 | 0.16 |  |  |
|  | 5-14 |  | 0.06 | 0.06 | 0.02 | 0.06 | 0.04 | 0.06 | 0.02 | 0.00 | 0.04 |  |  |
|  | 15-39 |  | 0.10 | 0.12 | 0.12 | 0.16 | 0.04 | 0.13 | -0.22 | 0.00 | 0.34 |  |  |
|  | 40-64 |  | 0.11 | -0.08 | 0.06 | 0.21 | 0.26 | 0.47 | 0.61 | 0.48 | 0.45 |  |  |
|  | 65+ |  | 0.26 | -0.20 | -0.16 | 0.34 | 0.41 | 0.19 | **0.68** | 0.52 | 0.67 |  |  |
| Japan | 0-4 | 2.05 | 1.12 | 1.12 | 0.54 | 0.32 | 0.28 | 0.21 | 0.11 | 0.04 | 0.11 | 0.06 | 0.01 |
|  | 5-14 | 0.25 | 0.15 | 0.13 | 0.07 | 0.08 | 0.05 | 0.04 | 0.02 | 0.01 | 0.03 | 0.01 | 0.00 |
|  | 15-39 | 1.37 | 0.51 | 0.45 | 0.15 | 0.37 | 0.25 | 0.10 | 0.10 | 0.07 | 0.02 | 0.07 | 0.04 |
|  | 40-64 | 0.81 | 0.44 | 0.47 | 0.47 | 0.62 | 0.50 | 0.25 | 0.33 | 0.17 | 0.19 | 0.25 | 0.28 |
|  | 65+ | 0.34 | 0.08 | 0.27 | 0.39 | **0.74** | 0.87 | 0.71 | 0.55 | 0.37 | 0.69 | 0.57 | 0.67 |
| Kazakhstan | 0-4 |  |  |  |  |  |  |  | 0.48 | -0.28 | 0.83 | 0.50 | -0.15 |
|  | 5-14 |  |  |  |  |  |  |  | -0.02 | 0.03 | 0.05 | 0.01 | 0.05 |
|  | 15-39 |  |  |  |  |  |  |  | -0.19 | -1.13 | -0.03 | -0.37 | 1.13 |
|  | 40-64 |  |  |  |  |  |  |  | 0.12 | -2.10 | 0.21 | -0.23 | 1.65 |
|  | 65+ |  |  |  |  |  |  |  | -0.03 | -0.81 | -0.02 | 0.25 | 0.37 |
| Kuwait | 0-4 |  |  |  |  |  | 0.63 | 0.97 |  |  | 0.14 |  |  |
|  | 5-14 |  |  |  |  |  | -0.02 | 0.13 |  |  | 0.06 |  |  |
|  | 15-39 |  |  |  |  |  | 0.36 | 0.43 |  |  | 0.03 |  |  |
|  | 40-64 |  |  |  |  |  | 0.43 | 0.72 |  |  | 0.59 |  |  |
|  | 65+ |  |  |  |  |  | 0.34 | 0.54 |  |  | 1.03 |  |  |
| Kyrgyzstan | 0-4 |  |  |  |  |  |  |  | 1.08 | 0.42 | 0.49 | -0.13 |  |
|  | 5-14 |  |  |  |  |  |  |  | 0.03 | 0.03 | 0.04 | 0.08 |  |
|  | 15-39 |  |  |  |  |  |  |  | -0.46 | -0.47 | 0.37 | 0.11 |  |
|  | 40-64 |  |  |  |  |  |  |  | -0.47 | -1.84 | 1.10 | -0.19 |  |
|  | 65+ |  |  |  |  |  |  |  | -0.18 | -0.76 | 0.68 | -0.28 |  |
| Latvia | 0-4 |  |  |  |  |  |  | 0.18 | 0.06 | -0.14 | 0.57 | 0.22 | 0.15 |
|  | 5-14 |  |  |  |  |  |  | -0.03 | -0.02 | 0.13 | 0.04 | 0.03 | 0.04 |
|  | 15-39 |  |  |  |  |  |  | 0.78 | -0.25 | -0.91 | 1.01 | 0.45 | 0.69 |
|  | 40-64 |  |  |  |  |  |  | 0.44 | -0.40 | -2.37 | 1.92 | 0.13 | 1.44 |
|  | 65+ |  |  |  |  |  |  | -0.04 | 0.11 | -0.42 | 0.51 | -0.02 | 0.50 |
| Lithuania | 0-4 |  |  |  |  |  |  |  | 0.13 | 0.04 | 0.29 | 0.14 | 0.22 |
|  | 5-14 |  |  |  |  |  |  |  | -0.01 | 0.06 | 0.04 | 0.02 | 0.06 |
|  | 15-39 |  |  |  |  |  |  |  | -0.27 | -0.62 | 0.58 | 0.03 | 0.60 |
|  | 40-64 |  |  |  |  |  |  |  | -0.51 | -1.78 | 1.61 | -0.70 | 1.05 |
|  | 65+ |  |  |  |  |  |  |  | 0.12 | -0.35 | 0.34 | -0.16 | 0.15 |
| Mauritius | 0-4 |  |  |  | 0.34 | 0.51 | 1.97 | 0.77 | 0.42 | 0.01 | 0.30 | 0.29 |  |
|  | 5-14 |  |  |  | 0.17 | 0.13 | 0.03 | 0.09 | 0.04 | 0.05 | -0.02 | 0.06 |  |
|  | 15-39 |  |  |  | 0.09 | -0.19 | -0.02 | 0.22 | 0.00 | -0.08 | 0.33 | 0.01 |  |
|  | 40-64 |  |  |  | 0.34 | -0.93 | 0.27 | 0.62 | 0.19 | 0.35 | 0.54 | 0.51 |  |
|  | 65+ |  |  |  | -0.02 | -0.51 | -0.06 | 0.72 | 0.36 | 0.51 | 0.38 | 0.04 |  |
| Mexico | 0-4 |  |  | 1.11 | 0.11 | 1.87 |  |  | 0.03 | 0.68 |  |  |  |
|  | 5-14 |  |  | 0.14 | 0.11 | 0.26 |  |  | 0.02 | 0.11 |  |  |  |
|  | 15-39 |  |  | 0.40 | 0.16 | 0.28 |  |  | 0.34 | 0.09 |  |  |  |
|  | 40-64 |  |  | 0.09 | 0.16 | 0.23 |  |  | 0.42 | 0.21 |  |  |  |
|  | 65+ |  |  | -0.27 | 0.51 | -0.05 |  |  | -0.02 | 0.19 |  |  |  |
| Netherlands | 0-4 | 0.50 | 0.31 | 0.20 | 0.18 | 0.21 | 0.23 | 0.08 | 0.11 | 0.13 |  | 0.07 | 0.09 |
|  | 5-14 | 0.07 | 0.01 | 0.01 | 0.02 | 0.07 | 0.04 | 0.04 | 0.02 | 0.01 |  | 0.03 | 0.01 |
|  | 15-39 | 0.23 | -0.02 | -0.04 | -0.02 | 0.12 | 0.09 | 0.10 | 0.04 | 0.03 |  | 0.16 | 0.10 |
|  | 40-64 | -0.12 | -0.04 | -0.32 | -0.14 | 0.20 | 0.37 | 0.23 | 0.36 | 0.28 |  | 0.44 | 0.37 |
|  | 65+ | -0.03 | 0.17 | -0.20 | -0.29 | 0.01 | 0.29 | 0.03 | 0.28 | 0.28 |  | **0.88** | 1.03 |
| New Zealand | 0-4 | 0.34 | 0.13 | 0.33 | 0.22 | 0.15 | 0.28 | 0.07 | 0.13 | 0.28 | 0.06 | 0.11 |  |
|  | 5-14 | 0.09 | 0.07 | 0.02 | -0.02 | 0.02 | 0.06 | 0.01 | 0.01 | 0.05 | 0.02 | 0.03 |  |
|  | 15-39 | 0.16 | 0.23 | -0.02 | -0.06 | 0.03 | 0.05 | 0.01 | -0.11 | 0.26 | 0.22 | 0.21 |  |
|  | 40-64 | 0.21 | -0.07 | -0.25 | -0.01 | 0.21 | 0.38 | 0.47 | 0.63 | 0.50 | 0.55 | 0.44 |  |
|  | 65+ | 0.18 | -0.09 | -0.31 | -0.01 | 0.26 | 0.25 | 0.44 | **0.77** | 0.51 | 0.91 | 1.10 |  |
| Norway | 0-4 |  | 0.27 | 0.24 | 0.13 | 0.33 | 0.27 | 0.01 | 0.06 | 0.31 | 0.04 | 0.10 | 0.06 |
|  | 5-14 |  | 0.03 | 0.02 | 0.03 | 0.04 | 0.05 | 0.04 | 0.03 | 0.02 | 0.04 | 0.01 | 0.00 |
|  | 15-39 |  | -0.01 | 0.12 | -0.05 | 0.09 | 0.06 | 0.01 | 0.07 | 0.15 | -0.17 | 0.26 | 0.14 |
|  | 40-64 |  | -0.16 | -0.20 | -0.18 | 0.15 | 0.10 | 0.24 | 0.34 | 0.54 | 0.42 | 0.44 | 0.25 |
|  | 65+ |  | -0.27 | -0.19 | -0.29 | 0.24 | 0.12 | 0.12 | 0.21 | 0.45 | **0.57** | 1.09 | 0.62 |
| Poland | 0-4 |  |  | 1.30 | 0.82 | 0.67 | 0.23 | 0.26 | 0.22 | 0.22 |  | 0.15 | 0.13 |
|  | 5-14 |  |  | 0.09 | 0.00 | 0.03 | 0.00 | 0.05 | 0.02 | 0.04 |  | 0.02 | 0.02 |
|  | 15-39 |  |  | 0.25 | -0.07 | -0.01 | -0.15 | 0.16 | -0.18 | 0.28 |  | 0.19 | 0.11 |
|  | 40-64 |  |  | 0.32 | -0.41 | -0.09 | -0.60 | -0.42 | -0.33 | 0.46 |  | 0.38 | 0.56 |
|  | 65+ |  |  | 0.06 | -0.31 | 0.39 | -0.20 | -0.14 | 0.09 | 0.29 |  | 0.55 | 0.47 |
| Portugal | 0-4 |  | 0.57 | 2.00 | 1.23 | 1.45 | 1.09 | 0.67 | 0.44 | 0.46 | 0.19 |  |  |
|  | 5-14 |  | 0.08 | 0.06 | 0.03 | 0.06 | 0.08 | 0.05 | 0.04 | 0.06 | 0.07 |  |  |
|  | 15-39 |  | 0.23 | 0.08 | -0.04 | -0.18 | 0.18 | 0.22 | -0.27 | -0.01 | 0.26 |  |  |
|  | 40-64 |  | 0.73 | -0.26 | 0.02 | 0.02 | 0.48 | 0.33 | 0.46 | 0.26 | 0.33 |  |  |
|  | 65+ |  | 0.54 | -0.15 | -0.23 | 0.04 | 0.56 | 0.59 | 0.43 | 0.42 | 0.52 |  |  |

| **Country** | **Age groups** | **1950-55** | **1955-60** | **1960-65** | **1965-70** | **1970-75** | **1975-80** | **1980-85** | **1985-90** | **1990-95** | **1995-2000** | **2000-05** | **2005-10** |
| --- | --- | --- | --- | --- | --- | --- | --- | --- | --- | --- | --- | --- | --- |
| Puerto Rico | 0-4 |  |  |  |  | 0.64 |  | 0.47 | 0.05 |  |  |  |  |
|  | 5-14 |  |  |  |  | 0.05 |  | 0.04 | 0.00 |  |  |  |  |
|  | 15-39 |  |  |  |  | -0.14 |  | 0.01 | -0.90 |  |  |  |  |
|  | 40-64 |  |  |  |  | 0.28 |  | -0.01 | -0.51 |  |  |  |  |
|  | 65+ |  |  |  |  | 0.15 |  | -0.16 | -0.26 |  |  |  |  |
| Republic of Korea | 0-4 |  |  |  |  |  |  |  | 0.17 | 0.01 | -0.09 | 0.09 | 0.11 |
|  | 5-14 |  |  |  |  |  |  |  | 0.10 | 0.07 | 0.09 | 0.04 | 0.03 |
|  | 15-39 |  |  |  |  |  |  |  | 0.22 | 0.23 | 0.57 | 0.39 | 0.05 |
|  | 40-64 |  |  |  |  |  |  |  | 0.83 | 0.48 | 0.80 | 1.03 | 0.75 |
|  | 65+ |  |  |  |  |  |  |  | 1.00 | -0.56 | 0.70 | 0.98 | 1.13 |
| Republic of Moldova | 0-4 |  |  |  |  |  |  |  | 0.83 | -0.13 | 0.31 | 0.52 | 0.00 |
|  | 5-14 |  |  |  |  |  |  |  | 0.06 | 0.01 | 0.06 | 0.06 | 0.04 |
|  | 15-39 |  |  |  |  |  |  |  | -0.19 | -0.26 | 0.47 | 0.19 | 0.19 |
|  | 40-64 |  |  |  |  |  |  |  | 0.21 | -1.39 | 0.67 | -0.48 | 0.58 |
|  | 65+ |  |  |  |  |  |  |  | 0.17 | -0.78 | 0.19 | -0.05 | 0.51 |
| Romania | 0-4 |  |  |  |  | 1.21 |  | 0.31 | -0.11 | 0.40 | 0.38 | 0.30 | 0.46 |
|  | 5-14 |  |  |  |  | 0.04 |  | 0.02 | 0.03 | -0.06 | 0.07 | 0.14 | 0.04 |
|  | 15-39 |  |  |  |  | 0.15 |  | 0.02 | -0.11 | -0.15 | 0.48 | 0.28 | 0.09 |
|  | 40-64 |  |  |  |  | 0.13 |  | -0.35 | -0.12 | -1.18 | 0.80 | 0.38 | 0.26 |
|  | 65+ |  |  |  |  | 0.36 |  | 0.08 | 0.25 | -0.30 | 0.38 | 0.16 | 0.33 |
| Russian Federation | 0-4 |  |  |  |  |  |  | 0.05 | 0.31 | 0.02 | 0.20 | 0.39 | 0.26 |
|  | 5-14 |  |  |  |  |  |  | 0.05 | 0.00 | 0.01 | 0.03 | 0.05 | 0.06 |
|  | 15-39 |  |  |  |  |  |  | 0.82 | -0.02 | -1.74 | 0.05 | -0.07 | 0.99 |
|  | 40-64 |  |  |  |  |  |  | 0.47 | 0.27 | -2.95 | 0.53 | -0.30 | 1.79 |
|  | 65+ |  |  |  |  |  |  | 0.03 | 0.23 | -0.52 | 0.06 | 0.02 | 0.37 |
| Singapore | 0-4 |  |  |  | 0.72 | 0.52 | 0.38 | 0.24 | 0.26 | 0.20 | 0.13 | 0.05 | 0.02 |
|  | 5-14 |  |  |  | 0.11 | 0.03 | 0.08 | 0.00 | 0.03 | 0.02 | 0.05 | 0.02 | 0.00 |
|  | 15-39 |  |  |  | 0.16 | 0.15 | 0.16 | 0.05 | 0.28 | 0.27 | 0.10 | 0.16 | 0.12 |
|  | 40-64 |  |  |  | 0.44 | 0.45 | 0.33 | 0.59 | 0.77 | 0.90 | 0.77 | 0.51 | 0.46 |
|  | 65+ |  |  |  | 0.61 | 0.17 | **0.80** | 0.59 | 1.14 | 0.50 | 0.92 | 1.25 | 0.58 |
| Slovenia | 0-4 |  |  |  |  |  |  |  | 0.38 | 0.27 | 0.15 | 0.07 | 0.15 |
|  | 5-14 |  |  |  |  |  |  |  | 0.04 | 0.02 | 0.03 | 0.04 | 0.02 |
|  | 15-39 |  |  |  |  |  |  |  | 0.10 | 0.09 | 0.26 | 0.28 | 0.34 |
|  | 40-64 |  |  |  |  |  |  |  | 0.47 | 0.45 | 0.58 | 0.68 | 0.70 |
|  | 65+ |  |  |  |  |  |  |  | 0.37 | 0.29 | 0.37 | **0.81** | 1.00 |
| Spain | 0-4 |  | 1.05 | 0.80 |  |  | 0.34 | 0.30 | 0.20 | 0.16 | 0.16 | 0.04 | 0.07 |
|  | 5-14 |  | 0.14 | 0.08 |  |  | 0.03 | 0.05 | 0.02 | 0.03 | 0.02 | 0.02 | 0.02 |
|  | 15-39 |  | 0.37 | 0.06 |  |  | 0.11 | -0.01 | -0.47 | 0.07 | 0.53 | 0.27 | 0.35 |
|  | 40-64 |  | 0.45 | 0.10 |  |  | 0.45 | 0.20 | 0.17 | 0.21 | 0.29 | 0.31 | 0.39 |
|  | 65+ |  | 0.42 | 0.22 |  |  | **0.79** | 0.42 | 0.24 | 0.48 | 0.35 | 0.59 | 0.70 |
| Sweden | 0-4 |  | 0.15 | 0.31 | 0.20 | 0.26 | 0.14 | 0.10 | 0.02 | 0.18 |  | 0.07 | 0.02 |
|  | 5-14 |  | 0.03 | 0.01 | 0.03 | 0.04 | 0.05 | 0.03 | 0.03 | 0.02 |  | -0.01 | 0.03 |
|  | 15-39 |  | 0.12 | -0.04 | -0.01 | 0.02 | 0.10 | 0.13 | 0.06 | 0.26 |  | 0.08 | 0.05 |
|  | 40-64 |  | 0.20 | -0.05 | -0.09 | -0.10 | 0.10 | 0.45 | 0.38 | 0.44 |  | 0.29 | 0.26 |
|  | 65+ |  | 0.02 | 0.03 | 0.09 | -0.05 | **0.21** | 0.40 | 0.49 | 0.56 |  | 0.72 | 0.64 |
| Switzerland | 0-4 |  | 0.36 | 0.41 | 0.27 | 0.33 | 0.30 | 0.14 | 0.05 | 0.17 | 0.03 | 0.07 | 0.10 |
|  | 5-14 |  | 0.08 | 0.00 | -0.02 | 0.09 | 0.04 | 0.04 | 0.03 | 0.02 | 0.03 | 0.02 | 0.02 |
|  | 15-39 |  | 0.11 | 0.19 | -0.04 | 0.11 | -0.03 | 0.10 | -0.21 | 0.23 | 0.46 | 0.24 | 0.20 |
|  | 40-64 |  | 0.19 | 0.15 | 0.21 | 0.44 | 0.24 | 0.38 | 0.33 | 0.39 | 0.37 | 0.41 | 0.28 |
|  | 65+ |  | 0.36 | -0.06 | 0.16 | **0.47** | 0.37 | 0.51 | 0.35 | 0.62 | 0.65 | 0.97 | 0.56 |
| TFYR Macedonia | 0-4 |  |  |  |  |  |  |  |  |  | 0.56 | 0.10 | 0.23 |
|  | 5-14 |  |  |  |  |  |  |  |  |  | 0.01 | 0.07 | 0.00 |
|  | 15-39 |  |  |  |  |  |  |  |  |  | -0.02 | 0.23 | 0.09 |
|  | 40-64 |  |  |  |  |  |  |  |  |  | 0.20 | 0.26 | 0.36 |
|  | 65+ |  |  |  |  |  |  |  |  |  | -0.03 | 0.21 | 0.27 |
| Tajikistan | 0-4 |  |  |  |  |  |  |  | 0.33 | 1.44 | 2.67 |  |  |
|  | 5-14 |  |  |  |  |  |  |  | 0.01 | -0.05 | 0.20 |  |  |
|  | 15-39 |  |  |  |  |  |  |  | -0.02 | -1.13 | 0.94 |  |  |
|  | 40-64 |  |  |  |  |  |  |  | -0.26 | -1.09 | 1.60 |  |  |
|  | 65+ |  |  |  |  |  |  |  | -0.32 | -0.71 | 0.36 |  |  |
| Trinidad and Tobago | 0-4 |  |  |  |  | 1.35 | 1.55 | 0.82 | 0.07 |  |  |  |  |
|  | 5-14 |  |  |  |  | 0.07 | 0.01 | 0.07 | -0.01 |  |  |  |  |
|  | 15-39 |  |  |  |  | 0.09 | -0.03 | -0.30 | -0.04 |  |  |  |  |
|  | 40-64 |  |  |  |  | 0.18 | 0.21 | -0.72 | 0.35 |  |  |  |  |
|  | 65+ |  |  |  |  | 0.06 | 1.11 | 0.89 | -0.36 |  |  |  |  |
| Turkmenistan | 0-4 |  |  |  |  |  |  |  | 1.52 | 0.25 |  |  |  |
|  | 5-14 |  |  |  |  |  |  |  | -0.04 | 0.02 |  |  |  |
|  | 15-39 |  |  |  |  |  |  |  | 0.04 | -0.13 |  |  |  |
|  | 40-64 |  |  |  |  |  |  |  | -0.23 | -0.40 |  |  |  |
|  | 65+ |  |  |  |  |  |  |  | -0.11 | -0.42 |  |  |  |
| Ukraine | 0-4 |  |  |  |  |  |  |  | 0.13 | -0.06 | 0.22 | 0.23 |  |
|  | 5-14 |  |  |  |  |  |  |  | -0.01 | 0.02 | 0.04 | 0.03 |  |
|  | 15-39 |  |  |  |  |  |  |  | -0.42 | -0.95 | 0.11 | -0.22 |  |
|  | 40-64 |  |  |  |  |  |  |  | -0.41 | -2.08 | 0.26 | -0.56 |  |
|  | 65+ |  |  |  |  |  |  |  | 0.08 | -0.56 | 0.14 | -0.08 |  |

| **Country** | **Age groups** | **1950-55** | **1955-60** | **1960-65** | **1965-70** | **1970-75** | **1975-80** | **1980-85** | **1985-90** | **1990-95** | **1995-2000** | **2000-05** | **2005-10** |
| --- | --- | --- | --- | --- | --- | --- | --- | --- | --- | --- | --- | --- | --- |
| United Kingdom | 0-4 | 0.53 | 0.25 | 0.19 | 0.14 | 0.17 | 0.40 | 0.22 | 0.14 | 0.18 |  |  | 0.07 |
|  | 5-14 | 0.11 | 0.01 | 0.00 | 0.02 | 0.03 | 0.03 | 0.03 | 0.02 | 0.03 |  |  | 0.02 |
|  | 15-39 | 0.31 | 0.04 | 0.02 | 0.11 | 0.01 | 0.07 | 0.11 | -0.06 | 0.04 |  |  | 0.11 |
|  | 40-64 | 0.38 | 0.10 | 0.07 | 0.10 | 0.22 | 0.32 | 0.50 | 0.56 | 0.45 |  |  | 0.28 |
|  | 65+ | 0.19 | 0.07 | 0.06 | -0.01 | 0.16 | 0.36 | 0.45 | 0.55 | 0.54 |  |  | 0.96 |
| United States of America | 0-4 | 0.25 | 0.05 | 0.09 | 0.40 | 0.33 | 0.30 | 0.17 | 0.09 | 0.19 | 0.08 | 0.02 |  |
|  | 5-14 | 0.07 | 0.02 | 0.02 | 0.01 | 0.04 | 0.04 | 0.03 | 0.02 | 0.02 | 0.03 | 0.01 |  |
|  | 15-39 | 0.18 | 0.09 | -0.13 | -0.21 | 0.22 | 0.11 | 0.18 | -0.18 | 0.07 | 0.47 | 0.05 |  |
|  | 40-64 | 0.40 | 0.00 | -0.04 | 0.06 | 0.59 | 0.60 | 0.44 | 0.30 | 0.18 | 0.50 | 0.19 |  |
|  | 65+ | 0.22 | -0.10 | -0.07 | 0.12 | 0.39 | 0.44 | 0.25 | **0.43** | 0.32 | 0.46 | 0.76 |  |
| Uruguay | 0-4 |  | -0.43 |  | 0.16 | -0.11 |  | 0.54 | 0.68 |  |  |  |  |
|  | 5-14 |  | 0.03 |  | -0.01 | 0.04 |  | 0.03 | 0.03 |  |  |  |  |
|  | 15-39 |  | 0.01 |  | -0.05 | 0.05 |  | 0.31 | -0.22 |  |  |  |  |
|  | 40-64 |  | -0.14 |  | -0.24 | 0.21 |  | 0.46 | 0.02 |  |  |  |  |
|  | 65+ |  | -0.56 |  | -0.45 | 0.42 |  | 0.11 | 0.03 |  |  |  |  |
| Uzbekistan | 0-4 |  |  |  |  |  |  |  | 0.79 | 1.03 | 0.90 | 0.59 |  |
|  | 5-14 |  |  |  |  |  |  |  | 0.04 | 0.02 | 0.08 | 0.07 |  |
|  | 15-39 |  |  |  |  |  |  |  | 0.00 | -0.09 | 0.03 | 0.32 |  |
|  | 40-64 |  |  |  |  |  |  |  | -0.29 | -0.77 | 0.84 | 0.21 |  |
|  | 65+ |  |  |  |  |  |  |  | -0.09 | -0.85 | 0.37 | -0.02 |  |
| Venezuela | 0-4 |  | 2.05 | 0.57 | -0.05 | 0.55 | 1.14 |  | 0.10 |  |  |  |  |
|  | 5-14 |  | 0.32 | 0.04 | 0.08 | 0.09 | 0.12 |  | 0.01 |  |  |  |  |
|  | 15-39 |  | 0.23 | 0.12 | 0.13 | -0.25 | 0.04 |  | -0.15 |  |  |  |  |
|  | 40-64 |  | 0.54 | 0.32 | 0.01 | 0.05 | 0.69 |  | 0.07 |  |  |  |  |
|  | 65+ |  | 0.44 | -0.44 | -0.37 | 0.34 | 0.46 |  | 0.04 |  |  |  |  |
| Yugoslavia, Former | 0-4 |  |  |  | 1.18 | 1.37 | 0.62 | 0.24 | 0.58 |  |  |  |  |
|  | 5-14 |  |  |  | 0.01 | 0.05 | 0.02 | 0.03 | 0.04 |  |  |  |  |
|  | 15-39 |  |  |  | -0.12 | 0.16 | 0.03 | 0.12 | 0.07 |  |  |  |  |
|  | 40-64 |  |  |  | -0.47 | 0.37 | -0.25 | -0.16 | 0.18 |  |  |  |  |
|  | 65+ |  |  |  | -0.44 | 0.41 | 0.00 | 0.05 | 0.34 |  |  |  |  |
| Croatia | 0-4 |  |  |  |  |  |  |  | 0.44 | 0.15 | 0.18 | 0.18 | 0.06 |
|  | 5-14 |  |  |  |  |  |  |  | 0.04 | 0.06 | 0.03 | 0.02 | 0.01 |
|  | 15-39 |  |  |  |  |  |  |  | -0.84 | 0.79 | 0.48 | 0.14 | 0.24 |
|  | 40-64 |  |  |  |  |  |  |  | 0.01 | 0.47 | 0.27 | 0.80 | 0.48 |
|  | 65+ |  |  |  |  |  |  |  | 0.31 | -0.19 | -0.42 | 1.44 | 0.56 |
| Serbia | 0-4 |  |  |  |  |  |  |  |  |  |  | 0.25 | 0.13 |
|  | 5-14 |  |  |  |  |  |  |  |  |  |  | 0.05 | 0.03 |
|  | 15-39 |  |  |  |  |  |  |  |  |  |  | 0.21 | 0.17 |
|  | 40-64 |  |  |  |  |  |  |  |  |  |  | 0.24 | 0.50 |
|  | 65+ |  |  |  |  |  |  |  |  |  |  | 0.33 | 0.55 |

* The most positive contributing cause of death as well as age group of at least 0.10 life years was marked with green for each year. The most decreasing life expectancy of at least -0.10 life-years was marked red, respectively. ‘Other diseases’ were not considered. For each country the first of at least two following years with cardiovascular disease being the most positively contributing cause of death to the improvement in live expectancy was printed with bold letters as an indicator for the epidemiologic transition. Correspondingly, the same procedure was performed for the age group 65 years and older.
